# Multiscale connectivity framework for working memory network in paediatric acute lymphoblastic leukaemia survivors

**DOI:** 10.1093/braincomms/fcag137

**Published:** 2026-04-17

**Authors:** Rajikha Raja, John O Glass, Ruitian Song, Lisa M Jacola, Tushar Patni, Yimei Li, Wilburn E Reddick

**Affiliations:** Department of Radiology, St.Jude Children’s Research Hospital, Memphis, TN 38105, USA; Department of Radiology, St.Jude Children’s Research Hospital, Memphis, TN 38105, USA; Department of Radiology, St.Jude Children’s Research Hospital, Memphis, TN 38105, USA; Department of Psychology and Biobehavioral Sciences, St.Jude Children’s Research Hospital, Memphis, TN 38105, USA; Department of Biostatistics, St.Jude Children’s Research Hospital, Memphis, TN 38105, USA; Department of Biostatistics, St.Jude Children’s Research Hospital, Memphis, TN 38105, USA; Department of Radiology, St.Jude Children’s Research Hospital, Memphis, TN 38105, USA

**Keywords:** acute lymphoblastic leukaemia, structural connectivity, working memory, paediatric cancer survivors, white matter

## Abstract

Working memory impairments are a common late effect in survivors of childhood acute lymphoblastic leukaemia, yet the structural network substrates of these difficulties remain poorly defined. Existing connectomic studies often rely on whole-brain parcellations, overlooking working memory-associated circuitry and multiscale organization. We developed a multiscale structural connectivity framework to investigate working memory-associated networks using diffusion MRI and performed a cross-sectional study with 70 acute lymphoblastic leukaemia survivors and 70 age and sex matched healthy controls. Working memory-relevant regions were identified based on functional activation patterns, and structural connectomes were constructed at two spatial scales: a fine-scale 76-node network and a coarser 24-node network derived from spatially contiguous, architecturally and functionally coherent regional groupings, as defined in the multimodal parcellation atlas of Human Connectome Project. Graph theoretical metrics, clustering coefficient, Eigenvector centrality, local assortativity and participation coefficient were computed to assess local network topology. Group comparisons were conducted with false discovery rate correction for multiple comparisons. Compared to healthy controls, survivors exhibited marked topological shifts. Specifically, clustering and assortativity were increased in the caudate, putamen and thalamus but decreased in the frontoparietal cortex. In contrast, centrality and participation showed the opposite pattern, signalling subcortical segregation and cortical hyperintegration. These effects were consistent across both spatial scales. Additional findings included scale-specific effects unique to the fine scale, as well as heterogeneous fine-scale patterns that resolved into consistent regional changes at the coarse scale. All effects remained significant after false discovery rate correction, highlighting the robustness of the network reorganization. Our framework combining a targeted working memory network with multiscale connectomic analysis proves its worth by revealing structural changes of working memory circuitry in survivors compared to healthy controls. The results show a broad reorganization, with weakened cortical networks and strengthened subcortical circuits, possibly as a form of compensation. These insights sharpen our understanding of treatment-related structural network alterations and point to new targets for future studies of cognitive outcomes and rehabilitation.

## Teaser

Raja *et al*. report that structural connections supporting working memory are altered in paediatric acute lymphoblastic leukaemia survivors. Using a multiscale network approach, disrupted patterns of segregation and integration in cortical and subcortical regions were identified. These findings highlight treatment-related effects on brain organization that are important for cognitive health.

## Graphical Abstract

**Graphical Abstract:**
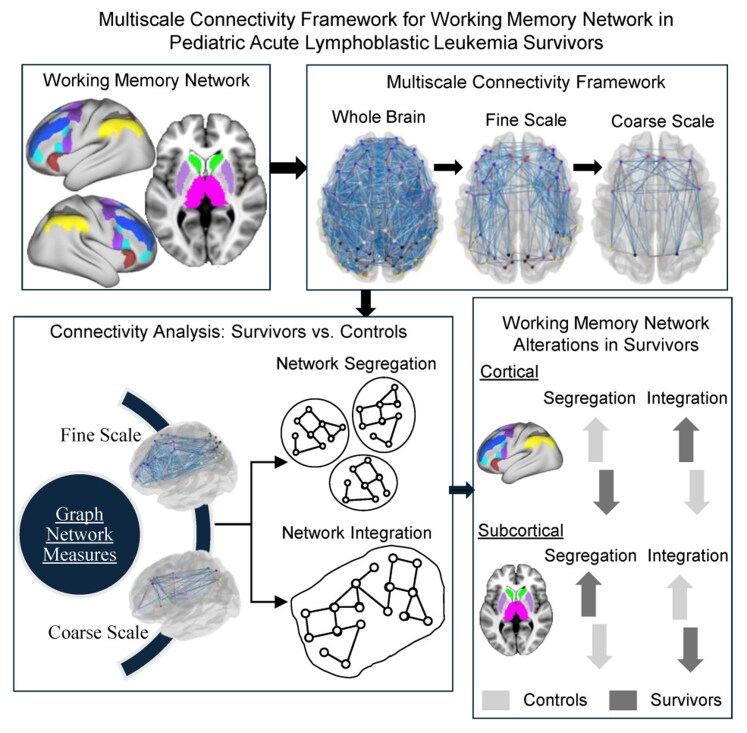

## Introduction

Working memory (WM) is a core cognitive system in the brain that enables the temporary storage and manipulation of information, and this short-term processing acts as the foundation for complex abilities such as learning, decision making and problem solving.^1^ Disruptions to WM can significantly impair an individual’s academic, occupational and social functioning and can have far-reaching consequences for functional independence and quality of life.^2,3^ WM impairments have been documented in various clinical populations, including childhood cancer survivors, who often face long-term neurocognitive challenges because of their treatment.^4^ As a result, significant research has focused on exploring the structural and functional organization of the brain, along with alterations related to WM and associated deficits.^5^ Identifying the neural underpinnings of neurocognitive late effects in clinical populations can inform targets for interventions to improve outcomes.^6^

In the quest to understand the structural changes underlying WM deficits, a growing body of work has focused on identifying and characterizing WM-related brain regions and networks.^7-9^ The brain regions and networks associated with WM involve a distributed set of cortical and subcortical nodes intricately connected to support the encoding, maintenance and retrieval of information.^5,10^ Traditionally, these attempts have relied on task-based functional MRI (fMRI) to pinpoint regions engaged during WM tasks such as N-back,^10,11^ or on resting state functional connectivity analyses to highlight communities of regions working in concert.^12,13^ However, inconsistencies arise from varied methodological choices, including different task contrasts, threshold settings and brain parcellation schemes, resulting in divergent WM region definitions across studies.^14^ These variations make it challenging to integrate functional insights into diffusion MRI connectomics or to reproduce and compare findings. To address this challenge, we derived and released a standardized list of 76 WM regions based on high-quality Human Connectome Project (HCP) task maps. This provides researchers with a reproducible framework for extracting a WM-associated sub-connectome, eliminating the need to perform additional fMRI analyses.

Survivors of acute lymphoblastic leukaemia (ALL), for instance, frequently report persistent difficulties in attention, executive functioning and WM.^4^ Neuroimaging studies link these deficits to diffuse white matter injury, especially within fronto-parietal and basal-ganglia pathways.^15^ Recent advances in neuroimaging and network science have highlighted the importance of structural connectivity for understanding the brain’s functional architecture.^16-18^ Kesler and colleagues reported that long-term ALL survivors show lower global efficiency and altered modular organization of the whole-brain connectome, changes that correlated with poorer executive performance.^19^ While the field has seen widespread application of whole-brain analyses, there is a growing need for approaches that focus on specific cognitive networks.^20,21^ Fine-grained examinations of WM related structural connectivity can reveal subtle alterations that remain obscured in global analyses.^22,23^ The availability of high-resolution cortical parcellations,^24,25^ diffusion MRI tractography and computational tools now makes it feasible to systematically derive WM-targeted sub-connectivity matrices. Although numerous studies have applied connectomics to investigate structural and functional brain networks, few have zeroed in on establishing a framework to perform WM-associated connectivity analysis.^26^ Existing approaches that integrate WM-related regions into structural network analyses often require multiple labour-intensive steps, subjective thresholds, or manual curation, making replication and comparison across studies difficult.^22^ The development of a framework for isolating WM-associated structural networks and enabling direct comparison of WM-related structural connectivity across different populations, conditions, or interventions would improve the consistency and interpretability of findings.

The present work aims to address this gap by introducing a multiscale framework that integrates robust definitions of WM-associated regions with subsequent structural connectivity analysis. Multiscale analyses are widely used in network neuroscience via hierarchical or multi-resolution parcellations, and it is well recognized that graph measures depend on scale and node definition.^14,23,27^ To capture the full spatial complexity of WM-associated organization, we evaluated the network at two complementary parcellation scales, a fine-scale and a coarse-scale. The fine-scale representation preserves the fine-grained anatomical detail of the HCP-MMP1 atlas and enables detection of subtle, subregional variations in WM-associated connectivity, showing patterns that may be heterogeneous or highly localized and are often diluted when parcels are spatially aggregated. In contrast, the coarse-scale consolidates spatially contiguous and functionally coherent parcels into broader anatomical units, yielding a more interpretable meso-scale summary that reduces analytic complexity and the multiple comparison burden. Prior work has shown that graph theoretical metrics are sensitive to the choice of parcellation granularity, and multiscale analyses can reveal effects that are either consistent across levels or visible only at specific resolutions.^14,23,27^ Accordingly, using both scales allows us to test whether group differences in WM-associated topology are focal and region-specific or reflect robust, convergent patterns across hierarchical spatial organization. This multiscale design provides a principled framework for examining the stability, specificity and interpretive richness of WM-associated network alterations in ALL survivors.

By applying this framework to both healthy controls (HC) and ALL survivors, we demonstrate how it can uncover nuanced differences in WM-related structural networks. Our overarching motivation is 2-fold: first, to develop a framework for WM network extraction and multiscale connectivity analysis that can be applied across studies, and second, to harness this method to gain clinically meaningful insights into how WM connectivity differs in paediatric ALL survivors, a population that is vulnerable to treatment-related neurocognitive difficulties. Ultimately, this approach can guide future research towards more targeted interventions that improve cognitive outcomes and quality of life for individuals impacted by ALL and other neurological conditions involving WM impairments.

## Materials and methods

### Participants

Participants included in this study belong to St. Jude Total Therapy Study 16 cohort (ClinicalTrials.gov identifier NCT00549848), which enrolled 598 patients (aged 0–18 years at diagnosis) between 2007 and 2017 to study the treatment effects in paediatric ALL patients aiming to improve the cure rate of children with ALL. Detailed treatment protocols and primary outcomes of Total Therapy Study 16 have been reported previously.^28,29^ All participants received risk-directed chemotherapy under the protocol approved by the institutional review board, with written informed consent obtained from parents or legal guardians (and assent from minors where applicable). As part of the therapeutic protocol, patients were imaged at completion of treatment, ∼2.5 years after diagnosis. Exclusion criteria for the current study included prior cranial radiation therapy for central nervous system (CNS) relapses, any history of relapses or secondary neoplasm, the presence of a genetic disorder known to affect cognition, or a significant history of head trauma or other neurological conditions unrelated to cancer therapy. From this group, 97 participants were selected for this study based on the availability of advanced diffusion MRI data acquired with a *b*-value of 1500 s/mm^2^ and in 64 gradient directions. Clinical characteristics, including age at diagnosis and treatment risk arm, were obtained from study records for all included participants. HC were drawn from the publicly available Human Connectome Project—Development (HCP-D) dataset^30,31^ which enrolled 652 typically developing children and adolescents. From this cohort, 176 participants with complete diffusion MRI acquisitions of sufficient quality were identified for potential inclusion. The HCP-D applied its own eligibility criteria, excluding individuals with significant neurological or psychiatric conditions, ensuring a healthy comparison group appropriate for developmental studies. These HC participants served as the pool from which matched controls were later selected for the present analysis.

### MRI data acquisition

All MRI data were acquired on a 3T Siemens Prisma scanner using a 64-channel head coil. Diffusion weighted imaging (DWI) data were collected using a single-shell spin-echo echo-planar imaging (EPI) sequence with the following parameters: 64 diffusion weighted directions with a *b*-value of 1500 s/mm^2^ and one non-diffusion weighted (*b* = 0 s/mm^2^) volume. Sequence parameters were: repetition time (TR) = 4000 ms, echo time (TE) = 77.4 ms, voxel size = 1.8 × 1.8 × 1.8 mm^3^, and 108 contiguous axial slices covering the entire brain. Additionally, high-resolution 3D T1-weighted anatomical images were also acquired using a magnetization prepared rapid gradient echo (MPRAGE) sequence with TR = 1800 ms, TE = 2.26 ms, inversion time (TI) = 900 ms, flip angle = 9°, voxel size = 1.0 × 1.0 × 1.0 mm^3^. Diffusion MRI data from the HCP-D dataset were resampled to match the ALL acquisition protocol of *b*-value = 1500 s/mm^2^ and 64 gradient directions.

### Design of multiscale structural connectivity framework

We developed a targeted structural connectivity framework to isolate and analyse white matter pathways specifically associated with WM. Our framework was designed to analyse WM networks at two scales: a fine 76-node scale to capture subregional specificity, and a coarser 24-node scale to enable broader anatomical interpretation and cross-scale comparisons. This section outlines the key stages of the framework, including the identification of WM-related regions, diffusion MRI processing and the construction of WM-associated structural connectomes.

#### WM network

**Figure 1:**
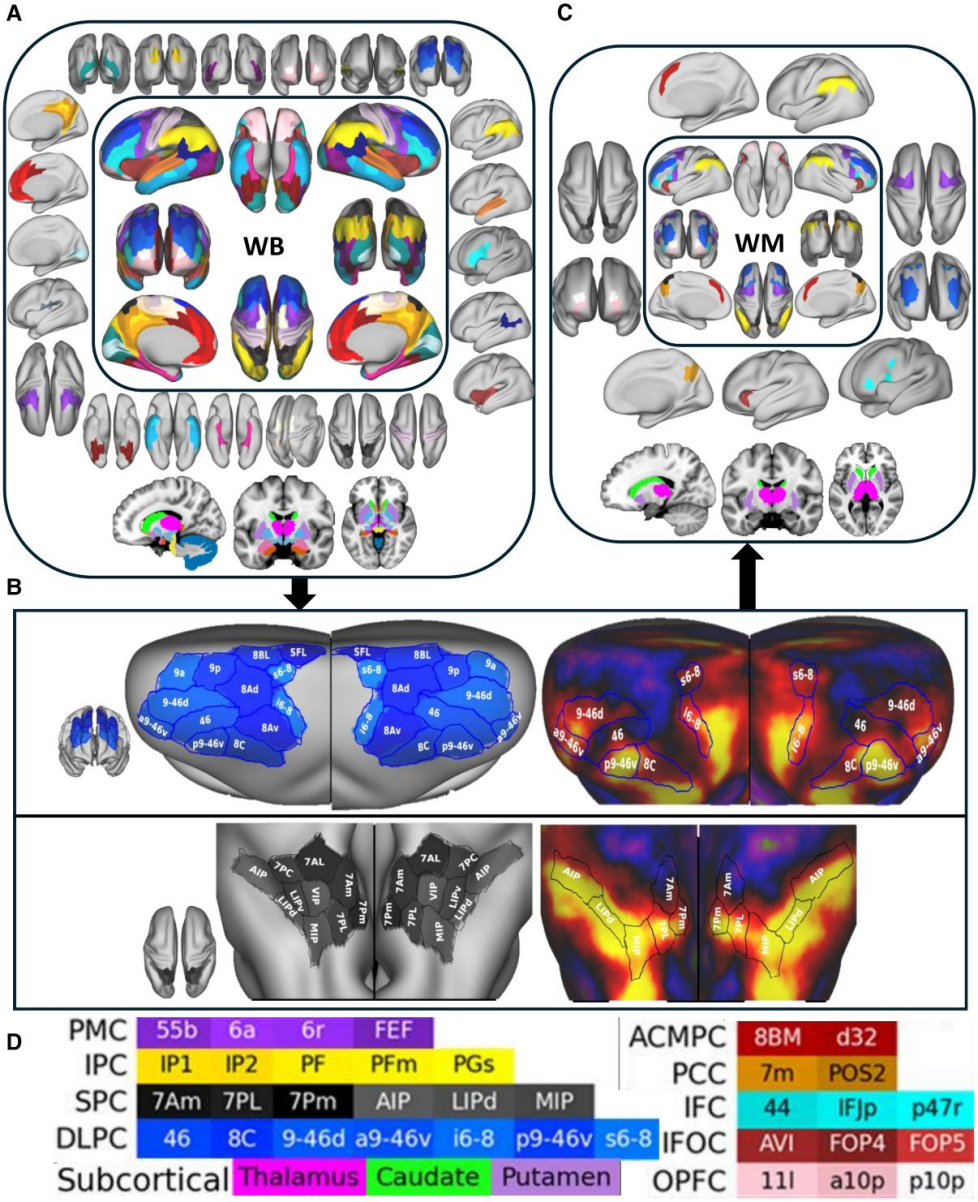
**Framework for defining the working memory (WM) network based on the HCP-MMP1 atlas.** (**A**) Illustrate initial selection of atlas and corresponding parcellation with visualization of the 180 bilateral cortical regions delineated by the HCP-MMP1 atlas and eight selected subcortical regions, along with the cerebellum and brainstem. Detailed legend indicating each cortical and subcortical region with colour-coded labels corresponding to visualizations in A are provided in Supplementary Fig. 1. (**B**) Identification of predominantly activated regions using task-fMRI contrast maps (2-back versus 0-back), shown for 2 representative regions, DLPC and SPC. Activation maps are overlaid onto the cortical surface, highlighting regions involved in WM tasks. The cortical folding maps and activation maps were adapted from the datasets available in BALSA at https://balsa.wustl.edu/^25,65,66^ with customized labels. (**C**) Selected cortical and subcortical regions included in the final WM network. (**D**) Colour-coded legend indicating the final set of cortical and subcortical WM network regions displayed in panel C. Abbreviations: WB, whole-brain; ACMPC, anterior cingulate and medial prefrontal cortex; DLPC, dorsolateral prefrontal cortex; IFC, inferior frontal cortex; IFOC, insular and frontal opercular cortex; IPC, inferior parietal cortex; OPFC, orbital and polar frontal cortex; PCC, posterior cingulate cortex; PMC, premotor cortex; SPC, superior parietal cortex.

We begin with a recognized, standard atlas called, multimodal parcellation atlas of Human Connectome Project (HCP-MMP1),^25^ which forms the anatomical basis of our WM node definitions. The rationale for selecting the HCP-MMP1 atlas was based on its multimodal integration, high spatial resolution and neuroanatomical precision. Figure 1 provides an overview of the WM network definition workflow, illustrating the process of narrowing from the whole-brain HCP-MMP1 atlas to a specific set of functionally defined WM regions. The atlas offers a high-resolution map of 180 bilateral cortical regions (Fig. 1A; Supplementary Fig. 1). Glasser *et al*. have published task-contrast maps (2BK-0BK) that highlight regions strongly activated during a WM task. The availability of the 2BK-0BK task contrast within this atlas provided an added advantage due to the functional specificity of these contrast maps in accurately identifying WM regions. Transitioning from manual inspection of these activation maps to generate WM node set is the key idea of this framework. This step involves a careful, manual review of the 2BK-0BK maps to verify which HCP-MMP1 regions exhibit robust WM-related activation. The aim is to retain nodes that show consistent involvement in WM processes, while excluding areas with negligible or inconsistent activation (Fig. 1B). We identified cortical regions exhibiting strong activation in the 2BK-0BK contrast maps, resulting in a set of 76 WM-related regions derived from the HCP-MMP1 atlas (Fig. 1C, D). These regions comprised 35 bilateral cortical regions and 3 bilateral subcortical regions. This initial node selection step ensures that the starting point of the framework is grounded in functionally validated, neuroanatomically precise data.

#### MRI data processing

**Figure 2:**
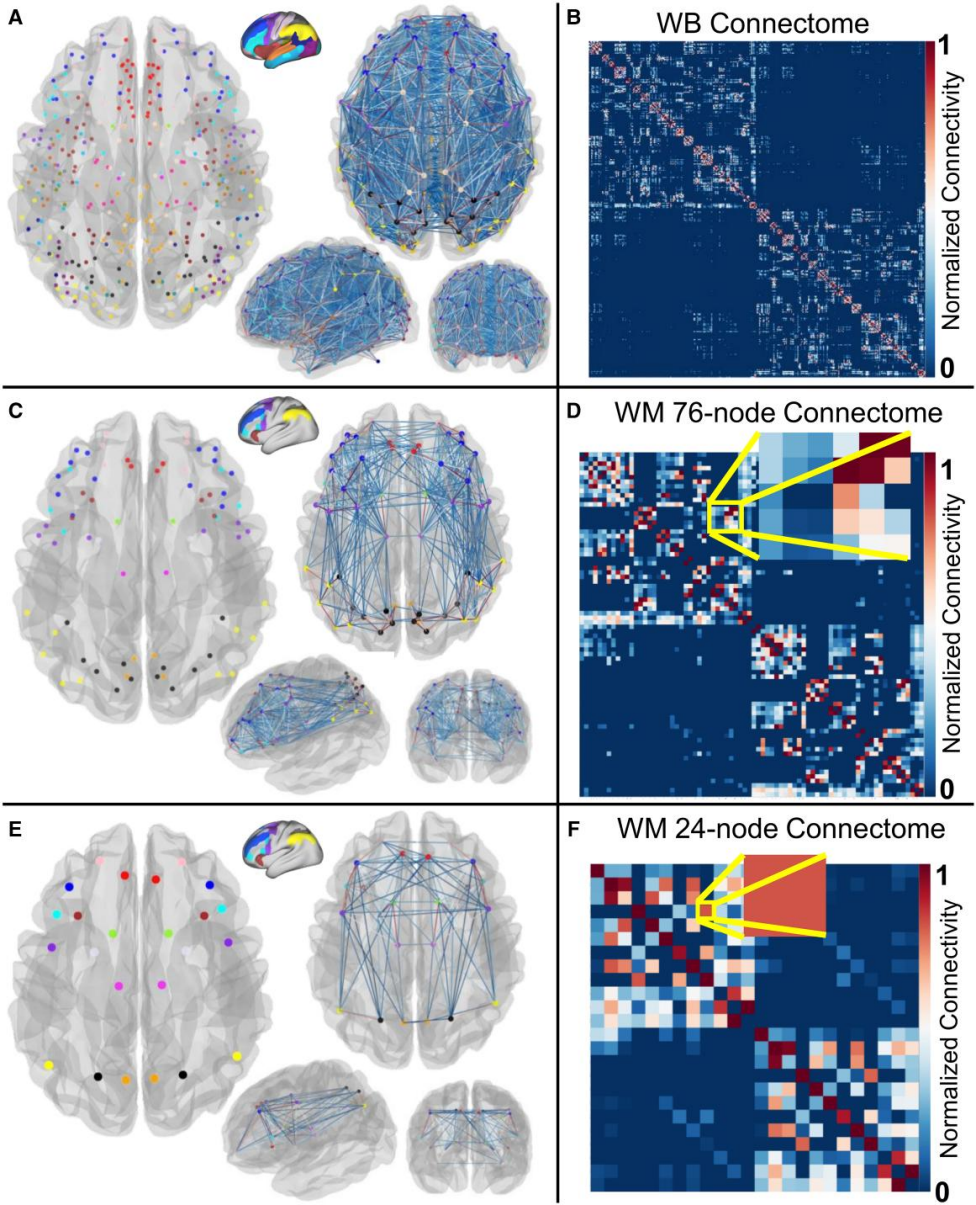
**Multiscale construction of working memory (WM) structural connectivity matrices.** This figure illustrates the hierarchical framework used to extract WM-associated structural connectomes from the whole-brain (WB) connectome and to generate multiscale representations. (**A** and **B**) Whole-brain structural connectome based on the HCP-MMP1.0 parcellation (379 regions). (**A**) Nodes are visualized anatomically. The inflated cortical maps were adapted from the datasets available in BALSA at https://balsa.wustl.edu/^25,65,66^ with customized colours. (**B**) The corresponding 379 × 379 group-averaged connectivity matrix is shown. (**C** and **D**) A 76-node WM subnetwork was extracted from the WB connectome. (**C**) WM-related nodes and their connectivity profiles are visualized. (**D**) The resulting 76 × 76 connectivity submatrix is shown. The inset highlights matrix entries representing pairwise connections between WM parcels assigned to left inferior parietal cortex (IPC) and left superior parietal cortex (SPC). (**E** and **F**) Coarse-scale network construction. The 76 WM nodes were grouped into 24 broader regions, consisting of nine bilateral cortical and three bilateral subcortical divisions. (**E**) The 24-scale nodes and their connectivity profiles are visualized. (**F**) The resulting 24 × 24 connectivity matrix is shown. The inset shows the final aggregated edge weight between IPC left and SPC left, corresponding to the sum of the matrix elements highlighted in panel D. The ordering of nodes in the WB, 76-node, and 24-node connectivity matrices is provided in Supplementary Tables 2–4, respectively.

Diffusion MRI data were processed using a custom pipeline comprising four main stages: preprocessing, reconstruction, tractography and connectome matrix generation. Initially, raw diffusion weighted images were corrected for artefacts including eddy current induced distortions, susceptibility related distortions and subject motion using FSL’s *eddy* and *topup* tools.^32^ Preprocessed diffusion data were then reconstructed using the constrained spherical deconvolution model in MRtrix3 to estimate fibre orientation distribution (FOD) functions.^33,34^ This model enables accurate representation of multiple fibre populations within a voxel, supporting reliable tractography in regions with complex fibre architecture. As a next step, whole-brain probabilistic tractography was performed using the iFOD2 algorithm in MRtrix3 on the reconstructed FOD images.^35^ An initial tractogram containing 20 million streamlines was generated to ensure comprehensive coverage of white matter pathways. This tractogram was then refined using Spherical deconvolution Informed Filtering of Tractograms (SIFT2) to improve biological accuracy by reducing biases in streamline density.^36^ The final tractogram consisted of 200 000 streamlines, each assigned a weight based on SIFT2 optimization. Finally, structural connectomes were derived from the SIFT2 weighted tractograms using the HCP-MMP1.0 parcellation, which delineates 379 cortical and subcortical brain regions. The parcellation image was registered to each subject’s diffusion space, and streamlines were assigned to region pairs. Connectivity matrices of size 379 × 379 were constructed using MRtrix3, where edge weights reflect the total SIFT2 assigned streamline weights between region pairs (Fig. 2A, B; Supplementary Table 2). To account for variability in region size and reduce the influence of node volume on connection strength, connectivity values were further normalized based on the inverse of the anatomical region volumes.

#### WM structural connectivity

WM structural connectivity matrices were constructed following a two-step hierarchical approach to analyse the connectomes at two spatial scales by systematically reducing the whole-brain connectivity matrix to WM-relevant subgraphs. Once the WM-related nodes were defined, we extracted their corresponding structural connectivity profiles from the whole-brain diffusion MRI-based connectomes. Each node served as a region within the WM network, and edges represented the reconstructed white matter pathways linking these WM nodes. By focusing our connectivity estimation on these functionally identified WM regions, we constructed a sub-connectivity matrix that emphasizes structural relationships relevant to WM processing, filtering out extraneous network connections not central to this cognitive domain (Fig. 2C, D; Supplementary Table 3).

The next step is anatomical grouping of WM nodes to facilitate higher-level interpretation of connectivity patterns. We grouped the identified WM-related nodes into broader cortical clusters. This aggregation was based on the multimodal cortical parcellation scheme defined in,^25^ in which the 180 bilateral cortical areas are organized into 22 neuroanatomically and functionally coherent broad regions. We adopted this scheme to assign the 35 bilateral WM-related cortical parcels to 9 bilateral cortical categories, which are anterior cingulate and medial prefrontal cortex (ACMPC), dorsolateral prefrontal cortex (DLPC), inferior frontal cortex (IFC), insular and frontal opercular cortex (IFOC), inferior parietal cortex (IPC), orbital and polar frontal cortex (OPFC), posterior cingulate cortex (PCC), premotor cortex (PMC) and superior parietal cortex (SPC), as listed in Table 1. Additionally, three bilateral subcortical regions (thalamus, caudate and putamen) were retained as distinct parcels, yielding a final set of 24 anatomically defined WM regions.

**Table 1:** List of 76 nodes identified as working memory network with the broader cortical region to which the node belongs to

Region	Nodes
Anterior cingulate and medial prefrontal cortex (ACMPC)	8BM, d32
Dorsolateral prefrontal cortex (DLPC)	8C, 9-46d, a9-46v, p9-46v, i6-8, s6-8, 46
Inferior frontal cortex (IFC)	IFJp, 44, p47r
Inferior parietal cortex (IPC)	PF, PFm, IP2, IP1, PGs
Insular and frontal opercular cortex (IFOC)	FOP4, FOP5, AVI
Orbital and polar frontal cortex (OPFC)	p10p, a10p, 11l
Posterior cingulate cortex (PCC)	POS2, 7m
Premotor cortex (PC)	6a, FEF, 55b, 6r
Superior parietal cortex (SPC)	AIP, LIPd, 7PL, 7Am, 7Pm, MIP
Caudate	Caudate
Putamen	Putamen
Thalamus	Thalamus

To compute the 24 × 24 coarse-scale connectivity matrix, we aggregated the corresponding entries from the 76 × 76 fine-scale matrix (Fig. 2E, F; Supplementary Table 4). Specifically, for each pair of coarse-scale regions, all interconnections between their constituent fine-scale nodes were identified, and the corresponding streamline weights were summed to form the edge weight in the coarse matrix. This procedure was applied symmetrically across rows and columns, ensuring that the coarse-scale matrix preserved total within and between group connectivity. This computation is visually demonstrated in Fig. 2, for computing the connection strength for the edge connecting left of IPC and SPC, where the zoomed inset in Fig. 2D highlights the fine-scale matrix entries used to derive the single coarse-scale edge shown in Fig. 2F. This summation-based approach retained the strength and distribution of connections while reducing dimensionality. Presenting the WM network at both node-level and the aggregated coarser-level preserves node-level resolution and supports higher-level interpretation of connectivity patterns across major anatomical systems.

### Local graph network metrics computation

To characterize the topological organization of the WM structural networks, we computed a set of local graph metrics for each node within the defined WM subnetwork. These metrics were chosen to capture complementary aspects of nodal connectivity and integration within the broader network architecture.^37^ Specifically, we computed four local metrics: clustering coefficient (CC), Eigenvector centrality (EC), local assortativity (LA) and participation coefficient (PC). Among these, CC and LA are primarily considered measures of local integration and structural organization, whereas EC and PC reflect the nodal influence and inter-modular communication within the global network.

The CC was used to quantify the extent to which a node’s neighbours are themselves interconnected, providing a measure of local connectivity density. To assess the global importance of each node, we computed EC, which accounts not only for a node’s direct connections but also for the centrality of its neighbours. The LA metric was included to evaluate the extent to which a node tends to connect with others of similar degree or strength, offering insight into local structural properties. Lastly, the PC was calculated to determine how evenly a node’s connections are distributed across different network modules, indicating its role in facilitating cross-network communication. To aid interpretation, we provide a summary of the four local graph metrics in Supplementary Table 1. All metrics were computed using NetworkX, a Python-based graph analysis library,^38^ applied to weighted, undirected connectivity matrices. Metric computation was performed for each subject individually at both the 76-node and 24-node WM network scales.

### Statistical analysis

To assess group differences in WM structural connectivity, we conducted statistical analysis on local graph network metrics derived from the WM structural connectomes. Analyses were performed at both the 76-node and 24-node scales. A multivariable linear regression model was used to compare ALL survivors and demographically matched HC, and heteroskedasticity-consistent robust standard errors were employed to ensure valid statistical inference. For each local graph metric and each node, the model included group ALL versus HC as the primary predictor, with age and sex included as covariates to control for potential confounding effects. False discovery rate (FDR) correction was applied to the resulting *P*-values across nodes to account for multiple comparisons, using the Benjamini–Hochberg procedure^39^ with a significance threshold of FDR corrected *P* < 0.05. All statistical analyses were conducted using R statistical software (version 4.4.2). The analysis utilized a complete dataset, eliminating the need for any data imputation procedures.

## Results

### Participant characteristics

From the initial pool of 97 ALL survivors and 176 eligible HCP-D participants, age- and sex-matched subjects were identified using group-level frequency matching on age and biological sex. Matching was based on age, using ±1-year tolerance bins and biological sex, ensuring identical male-to-female ratios in both groups. This resulted in a final sample of 140 participants, comprising 70 ALL survivors and 70 HCs. A small residual difference in mean age remained between groups (*P* = 0.09). Consistent with best practices in observational matched-cohort analyses, age and sex were included as covariates in all statistical models to address residual imbalance and improve precision.^40,41^ Demographic and clinical characteristics of the final matched sample are summarized in Table 2. The demographic profile of the final ALL cohort was comparable to that of the overall Total Therapy Study 16 population, supporting the representativeness of this subgroup.

**Table 2:** Demographic and clinical characteristics of study participants

Characteristic	ALL survivors	Healthy controls
*N*	70	70
Age at imaging, mean (SD), range (years)	11.84 (4.13), 6.2–20.3	12.93 (3.37), 6.4–20.3
Sex (M/F)	40/30	40/30
Age at diagnosis, mean (SD), range (years)	9.3 (4.1), 3.7–17.7	NA
Risk arm, *n* (%)	Low: 34 (48.6%), standard/high: 36 (51.4%)	NA

### Group differences in local graph metrics at the 76-node scale

**Figure 3:**
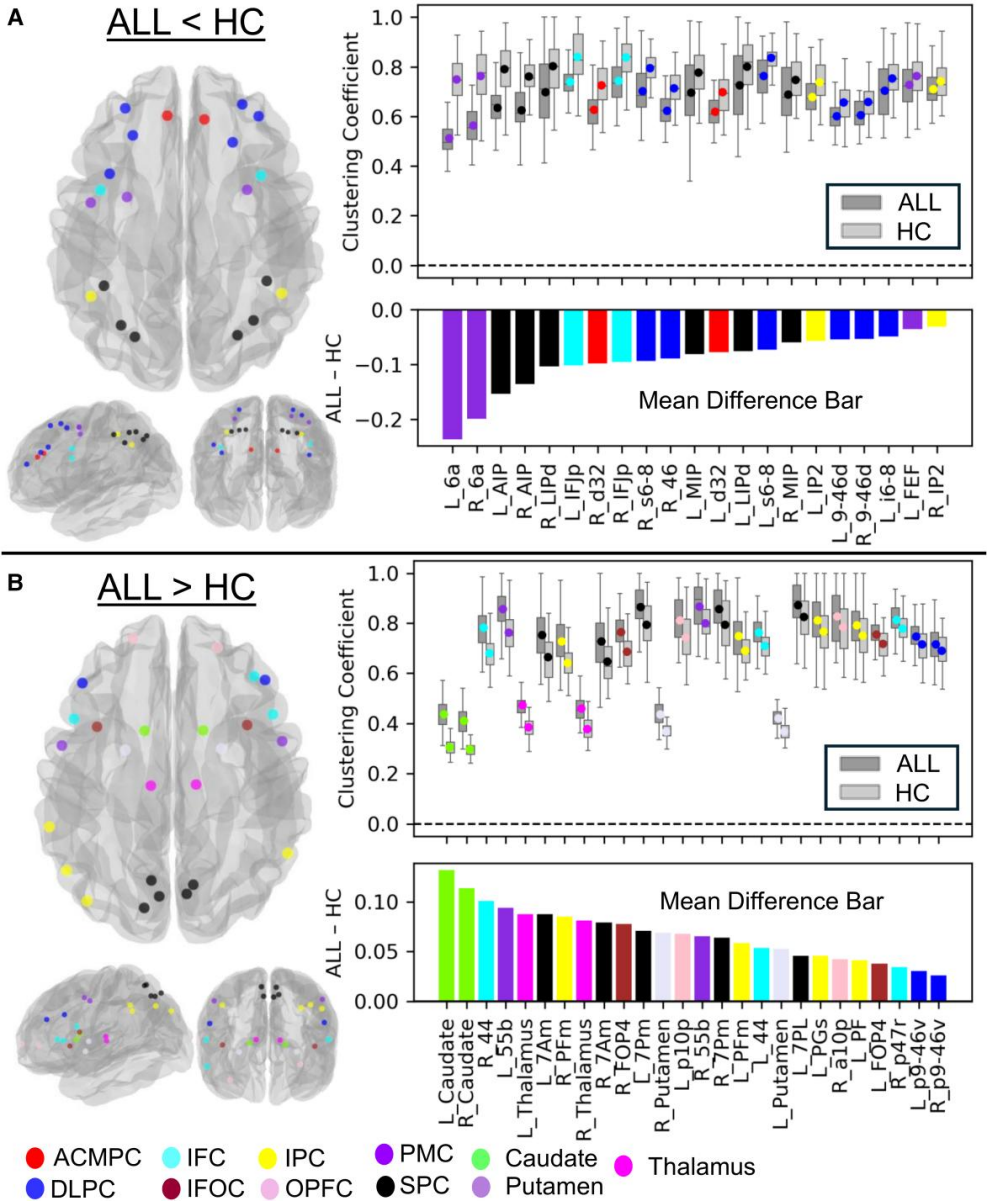
**Group differences in clustering coefficient (CC) across the 76-node working memory (WM) structural network.** (**A**) Panel shows regions where CC was lower in acute lymphoblastic leukaemia (ALL) survivors compared with healthy controls (HC) (ALL < HC), and (**B**) panel shows regions where CC was higher in ALL survivors (ALL > HC). In each panel, the left subpanel displays glass brain plots highlighting significant cortical and subcortical nodes. The upper right subpanel shows boxplots illustrating the distribution of CC values for ALL and HC groups at each significant node. The lower right subpanel presents bar plots of the mean group difference (ALL—HC) in CC, indicating the direction and magnitude of effects. *x*-axis labels are shared between boxplots and bar plots. Colour coding denotes cortical and subcortical regions as indicated in the legend at the bottom of the figure. Group differences were assessed using multivariable linear regression with group (ALL versus HC) as the primary predictor and age and sex as covariates, with false discovery rate (FDR) correction applied for multiple comparisons (*P* < 0.05). The full set of regression estimates, raw *P*-values and FDR-corrected *P*-values for all nodes is provided in Supplementary Table 5. The final sample included *N* = 70 ALL survivors and *N* = 70 HCs. Abbreviations: ACMPC, anterior cingulate and medial prefrontal cortex; DLPC, dorsolateral prefrontal cortex; IFC, inferior frontal cortex; IFOC, insular and frontal opercular cortex; IPC, inferior parietal cortex; OPFC, orbitofrontal and polar frontal cortex; PMC, premotor cortex; SPC, superior parietal cortex.

**Figure 4:**
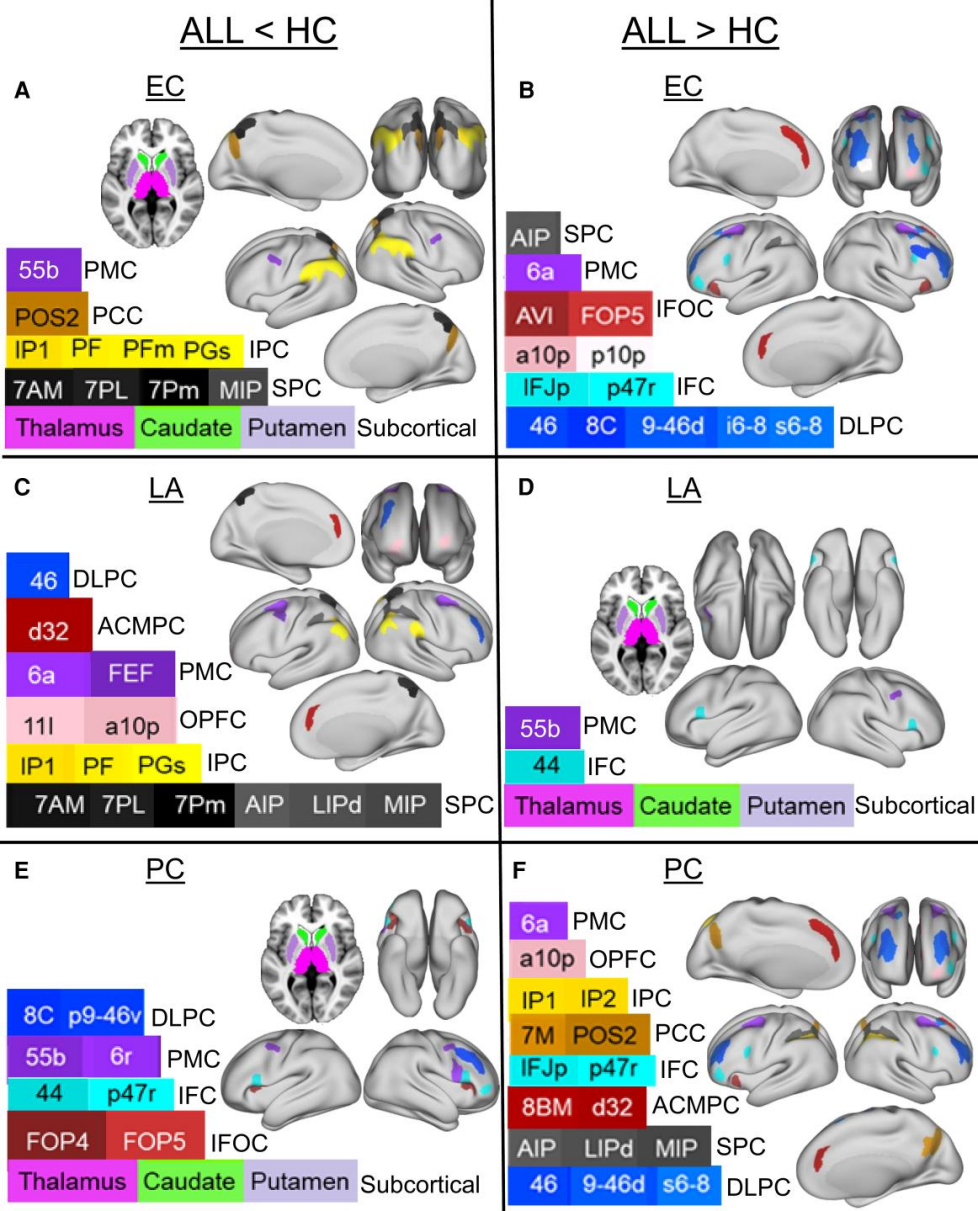
**Consolidated group differences in eigenvector centrality (EC), local assortativity (LA) and participation coefficient (PC) at the 76-node scale.** Panels A, C and E show regions with significantly reduced values in ALL survivors compared to controls (ALL < HC), while panels B, D and F show regions with significantly increased values in ALL survivors (ALL > HC). The inflated cortical maps were adapted from the datasets available in BALSA at https://balsa.wustl.edu/^25,65,66^ with customized labels. Full metric-specific glass brain plots (including boxplots and mean difference bars) are provided in Supplementary Figs 2–4. Colour coding denotes cortical and subcortical regions as indicated in the legends inside the panels. Group differences were assessed using multivariable linear regression with group (ALL versus HC) as the primary predictor and age and sex as covariates, with false discovery rate (FDR) correction applied for multiple comparisons (*P* < 0.05). The full set of regression estimates, raw *P*-values and FDR-corrected *P*-values for all nodes is provided in Supplementary Tables 6–8. The final sample included *N* = 70 ALL survivors and *N* = 70 HCs. Abbreviations: ACMPC, anterior cingulate and medial prefrontal cortex; DLPC, dorsolateral prefrontal cortex; IFC, inferior frontal cortex; IFOC, insular and frontal opercular cortex; IPC, inferior parietal cortex; OPFC, orbital and polar frontal cortex; PCC, posterior cingulate cortex; PMC, premotor cortex; SPC, superior parietal cortex.

**Figure 5:**
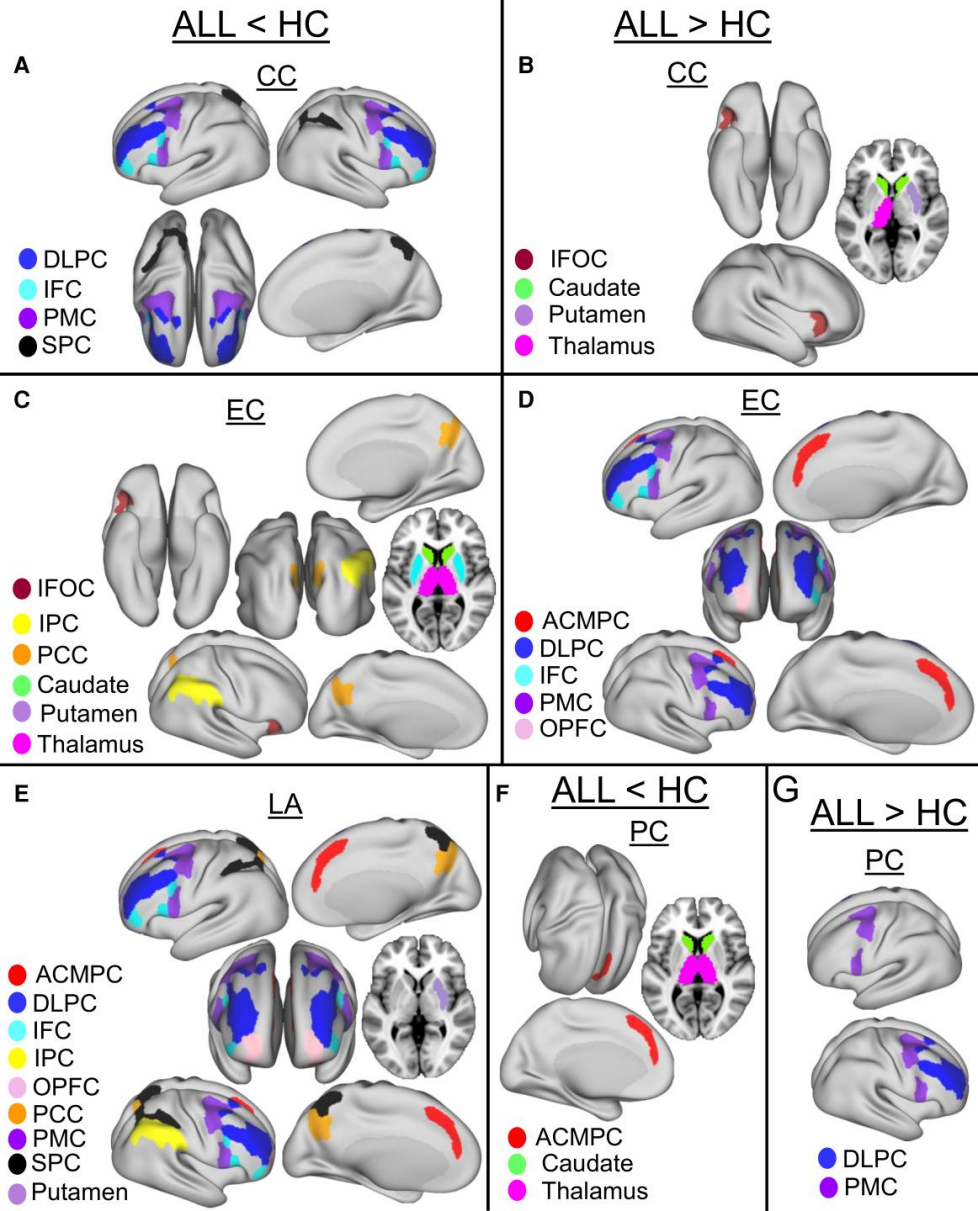
**Consolidated group differences in clustering coefficient (CC), eigenvector centrality (EC), local assortativity (LA) and participation coefficient (PC) at the 24-node scale.** Panels A, C, E and F show regions with significantly reduced values in ALL survivors compared to controls (ALL < HC), while panels B, D and G show regions with significantly increased values in ALL survivors (ALL > HC). The inflated cortical maps were adapted from the datasets available in BALSA at https://balsa.wustl.edu/^25,65,66^ with customized colours. Full metric-specific plots (including boxplots and mean difference bars) are provided in Supplementary Figs 5–8. Colour coding denotes cortical and subcortical regions as indicated in the legends inside the panels. Group differences were assessed using multivariable linear regression with group (ALL versus HC) as the primary predictor and age and sex as covariates, with false discovery rate (FDR) correction applied for multiple comparisons (*P* < 0.05). The full set of regression estimates, raw *P*-values and FDR-corrected *P*-values for all nodes is provided in Supplementary Tables 9–12. The final sample included *N* = 70 ALL survivors and *N* = 70 HCs. Abbreviations: ACMPC, anterior cingulate and medial prefrontal cortex; DLPC, dorsolateral prefrontal cortex; IFC, inferior frontal cortex; IFOC, insular and frontal opercular cortex; IPC, inferior parietal cortex; OPFC, orbital and polar frontal cortex; PCC, posterior cingulate cortex; PMC, premotor cortex; SPC, superior parietal cortex.

The results for each network metric are illustrated using graphical visualizations comprising glass brain plots, groupwise boxplots and mean difference bar plots. As an exemplar, Fig. 3 presents these plots for CC at the 76-node scale. The glass brain plots highlight the spatial distribution of significant nodes, providing anatomical context and revealing the localization and hemispheric patterning of altered connectivity across cortical and subcortical structures. The accompanying boxplots illustrate the distribution of metric values within each group, while the mean difference bar plots show raw group differences (ALL − HC) ranked to emphasize regions with the most prominent effects. Consolidated summaries of additional metrics at the 76-node and 24-node scales are provided in Figs 4 and 5, respectively, while full per-metric visualizations are available in Supplementary Figures, and detailed statistical results are presented in Supplementary Tables.

#### Clustering coefficient

Group differences in CC between ALL survivors and HC are presented in Fig. 3 and detailed in Supplementary Table 5. Regions with significantly reduced clustering in the ALL group (ALL < HC) are shown in Fig. 3A, while regions with increased clustering (ALL > HC) are shown in Fig. 3B. Regions with significantly reduced clustering in the ALL group (ALL < HC) include ACMPC, DLPC, IFC, IPC, PMC and SPC (Fig. 3A). These differences are further illustrated in the corresponding boxplots and mean difference bar plots, showing clear group separation (Fig. 3A). The most pronounced decreases are observed in SPC, PMC and DLPC. In contrast, regions with increased clustering in the ALL group (ALL > HC) include cortical regions such as OPFC, and IFOC and subcortical regions (caudate, thalamus and putamen) (Fig. 3B), with the strongest increases observed in ventral frontal and subcortical regions.

#### Eigenvector centrality

Summarized group differences in EC at the 76-node scale are shown in Fig. 4A, B, with full visualizations in Supplementary Fig. 2 and statistical details in Supplementary Table 6. Significant reductions in the ALL group were found in the cortical regions such as IPC, PCC, PMC and SPC and subcortical regions (caudate, thalamus and putamen) (Fig. 4A; Supplementary Fig. 2A). These reductions were particularly pronounced in posterior cortical and subcortical regions. Conversely, increased EC in the ALL group was observed in the cortical (ACMPC, DLPC, IFC, IFOC, OPFC, PMC and SPC) brain regions (Fig. 4B; Supplementary Fig. 2B). Elevated centrality was most prominent in prefrontal, parietal and associative cortical areas, as shown by the distribution plots and mean difference bars (Supplementary Fig. 2).

#### Local assortativity

Summarized group differences in LA are shown in Fig. 4C, D, with full visualizations in Supplementary Fig. 3 and statistical details in Supplementary Table 7. Regions with significantly reduced assortativity in the ALL group (ALL < HC) are shown in Fig. 4C and Supplementary Fig. 3A, while regions with increased assortativity (ALL > HC) are shown in Fig. 4D and Supplementary Fig. 3B. The ALL group exhibited significantly reduced LA in several higher-order cortical areas, including the ACMPC, DLPC, IPC, OPFC, PMC and SPC. These reductions were most prominent in SPC and IPC. In contrast, increased LA in the ALL group was detected in the cortical (IFC and PMC) and subcortical (caudate, putamen and thalamus) brain regions, with the most substantial increases occurring in subcortical regions.

#### Participation coefficient

Summarized group differences in PC are shown in Fig. 4E, F, with full visualizations in Supplementary Fig. 4 and statistical details in Supplementary Table 8. Regions with significantly reduced participation in the ALL group (ALL < HC) are shown in Fig. 4E and Supplementary Fig. 4A, while regions with increased participation (ALL > HC) are shown in Fig. 4F and Supplementary Fig. 4B. Reduced participation in the ALL group was found in cortical (DLPC, IFC, IFOC, PMC) and subcortical (caudate, putamen and thalamus) brain regions, with the most notable decreases observed in subcortical areas. On the other hand, increased participation was observed in the ACMPC, DLPC, IFC, IPC, IFOC, OPFC, PCC, PMC and SPC, primarily involving cortical association regions.

### Group differences in local graph metrics at the 24-node scale

#### Clustering coefficient

At the coarser 24-node scale, group differences in CC are summarized in Fig. 5A, B, with full visualizations in Supplementary Fig. 5 and statistical details in Supplementary Table 9. Regions with significantly reduced clustering in the ALL group (ALL < HC) are shown in Fig. 5A and Supplementary Fig. 5A, while those with increased clustering (ALL > HC) are shown in Fig. 5B and Supplementary Fig. 5B. The ALL group demonstrated significant reductions in clustering in regions including the DLPC, IFC, PMC and SPC, with the most marked decreases in PMC and DLPC. In contrast, elevated clustering in the ALL group was observed in IFOC and subcortical regions, including the caudate, thalamus and putamen. The largest increases were noted in the thalamus and caudate.

#### Eigenvector centrality

Group differences in EC at the 24-node scale are summarized in Fig. 5C, D, with full visualizations in Supplementary Fig. 6 and statistical details in Supplementary Table 10. Regions with significantly reduced centrality in the ALL group (ALL < HC) are shown in Fig. 5C and Supplementary Fig. 6A, while regions with increased centrality (ALL > HC) are shown in Fig. 5D and Supplementary Fig. 6B. The ALL group exhibited significantly reduced centrality in the cortical (IPC, PCC and IFOC) and subcortical (caudate, thalamus, putamen) regions, with the strongest effects observed in the thalamus, caudate and putamen. Conversely, increased centrality in the ALL group was identified in the ACMPC, DLPC, IFC, OPFC and PMC, with the most pronounced effects in the PMC and DLPC.

#### Local assortativity

Group differences in LA on the 24-node scale are summarized in Fig. 5E, with full visualizations in Supplementary Fig. 7 and statistical details in Supplementary Table 11. Decreased assortativity in the ALL group involved in both cortical (ACMPC, DLPC, IFC, IPC, OPFC, PCC, PMC and SPC) and subcortical (putamen) regions. The most pronounced reductions were observed in SPC, PCC and OPFC.

#### Participation coefficient

Group differences in PC at the 24-node scale are summarized in Fig. 5F, G, with full visualizations in Supplementary Fig. 8 and statistical details in Supplementary Table 12. Regions with significantly reduced participation in the ALL group (ALL < HC) are shown in Fig. 5F and Supplementary Fig. 8A, while regions with increased participation (ALL > HC) are shown in Fig. 5G and Supplementary Fig. 8B. The ALL group showed reduced participation in the ACMPC and subcortical (caudate and thalamus) with the most marked decreases observed in the thalamus and caudate. In contrast, increased participation was noted in the DLPC and PMC. Mean difference bar plots highlighted negative group differences in subcortical regions and positive differences in frontal-parietal areas.

## Discussion

This study provides a comprehensive characterization of brain regions commonly associated with WM at the end of protocol-directed chemotherapy treatment for childhood ALL, using a multiscale graph theoretical approach to analyse structural connectivity derived from diffusion MRI. By examining local graph metrics at both a fine grained 76-node and a coarser 24-node WM network scale, we identified consistent and spatially distributed disruptions in topological organization across multiple network dimensions, including local clustering, hubness, assortativity and inter-modular integration. The multiscale analysis revealed three broad categories of effects such as, consistent patterns that appeared at both parcellation scales, scale-specific alterations detectable only at the fine 76-node resolution and heterogeneous fine-scale shifts that either converged into clear regional changes or cancelled out when aggregated at the coarser 24-node level. In the paragraphs that follow we discuss these scenarios for each metric, showing how the spatial scale shapes the interpretation of WM associated network alterations. The observed differences in WM network organization between ALL survivors and controls are suggestive of treatment and diagnosis related effects. However, without explicit analyses linking imaging findings to treatment exposures, these observations should be interpreted cautiously. Future studies integrating treatment variables will be essential for establishing direct associations and clarifying mechanisms.

### Consistent cross-scale patterns

Increased CC in ALL survivors compared to HCs could be seen consistently across 76 and 24 scales in regions such as IFOC, caudate, putamen and thalamus (Figs 3B and 5B). This indicates tighter, highly segregated neighbourhoods within salience processing and cortico-striatal-thalamic motor loops.^37,42,43^ Crucially, similar over-segregation in these nodes has been linked to poorer WM performance in childhood ALL survivors and other cohorts.^44,45^ Increased EC in ALL survivors could be seen consistently across 76 and 24 scales in executive frontal areas such as ACMPC, DLPC, IFC, OPFC, but decreased in the PMC and the three basal-ganglia/thalamic nuclei (Figs 4B and 5D). These frontal regions overlap the canonical, highly reliable hubs mapped by,^46^ indicating a systematic but not random shift. Echoing Cole *et al.*,^47^ who tied stronger prefrontal hubness to better cognitive control, our findings suggest that survivors reroute network influence from motor-subcortical loops to executive hubs as a compensatory strategy to preserve cognition after treatment. The fronto-subcortical EC imbalances we observed align with prior reports of poorer WM performance in long-term ALL survivors^44,48^ and with mechanistic basal-ganglia gating studies.^45,49^ Together, these findings support the idea that EC alterations contribute to the WM deficits characteristic of this population.

Compared to HC, survivors of ALL showed decreased LA in both scales in several fronto-parietal regions such as ACMPC, DLPC, IPC, OPFC and SPC (Figs 4C and 5E). According to prior studies,^37,50^ lower LA in key nodes means they stop favouring similar partners, removing the network’s usual barriers against random crosstalk. Because the same hubs form the fronto-parietal control network,^13,51^ this crosstalk overload likely destabilizes the circuits that support attention and WM in ALL survivors. Finally, PC consistently declined in the caudate and thalamus (Figs 4E and 5F), denoting a stable retreat of these nodes from cross-modular communication and consonant with their EC demotion. Because PC quantifies the extent to which a node’s links span multiple modules,^37,52^ lower values in the caudate and thalamus point to a stable loss of their normal integrative, hub-like role.^42,53^ Reductions in striatal and thalamic integration have been tied to poorer WM and attention outcomes in childhood ALL survivors,^44,48^ supporting the functional significance of the PC decline observed here. Collectively, these consistent changes across scales may represent true distributed effects tied to cognitive dysfunction, such as cortical thinning and white matter degradation. These regions likely represent core hubs of pathological or compensatory reorganization in ALL survivors. The consistency across scales reinforces their role as key drivers of WM network alteration and suggests that both local and system-level architecture is affected in these areas.

### Fine-scale-only alterations

A critical strength of this study lies in the use of hierarchical anatomical modelling, which exposed a far broader spectrum of scale-dependent response patterns beyond the consistent alterations that would be obscured in any single-resolution analysis. We identified several distinct multiscale scenarios, each offering unique neurobiological implications. The first scenario involves effects that appeared only at the 76-node scale, suggesting highly focal or subregional topological differences that are too spatially localized to influence the coarser parcellation level. Previous scale-sensitive graph works demonstrated that regional effects can disappear when parcels are spatially averaged.^14,23^ In our cohort, these purely fine-scale effects comprised increased CC in OPFC and decreased CC in ACMPC, increased LA in the caudate and thalamus and increased PC in OPFC, IPC, PCC and SPC.

Increased CC in ALL survivors observed in the OPFC indicates a focal tightening of orbitofrontal neighbourhoods that does not propagate to larger functional modules (Fig. 3). Orbitofrontal micro-circuits are known to be especially locally interconnected.^54^ Moreover, a diffusion-MRI study of long-term childhood ALL survivors reported increased local segregation in orbitofrontal white matter regions alongside executive deficits,^48^ supporting the functional relevance of the OPFC CC rise observed here. Conversely, decreased CC seen in the ACMPC indicate subtle local fragmentation of cingulo-medial networks that remains undetected when nodes are pooled (Fig. 3). The ACMPC normally exhibits high local clustering to support conflict monitoring and WM updating.^13,55^ Studies in paediatric cohorts including childhood ALL survivors show that reduced anterior cingulate clustering or local efficiency predicts poorer attention and WM performance.^44,56^

### Heterogeneous fine-scale effects that converge at the coarse scale

The second scenario features heterogeneous changes at the 76-node level that, when pooled into 24-node parcels, sum to an overall increase or decrease. This indicates a meso-scale reallocation in which enough sub-parcels shift in the same direction to push the whole region towards hyper-integration, marked by higher PC or EC, or towards hypo-segregation, marked by lower CC or LA. Previous studies have illustrated this scenario as a biased-averaging phenomenon showing direction reversals across resolutions in diffusion and fMRI graphs in premotor and parietal hubs.^57^ In our study, this effect appeared in several regions, such as heterogeneous CC in DLPC, IFC, PMC and SPC with net coarse-scale reduction (Figs 3 and 5A), heterogeneous EC in PMC with net coarse-scale increase (Figs 4A, B and 5D), heterogeneous LA in PMC with net coarse-scale reduction (Figs 4C, D and 5E) and heterogeneous PC in DLPC and PMC with net coarse-scale increase (Figs 4E, F and 5G).

Reduced segregation and greater integration can both hurt WM in ALL survivors. Structural MRI from the St Jude Lifetime Cohort shows that lower clustering and local efficiency in fronto-parietal tracts predict later WM problems.^44^ Our coarse-scale CC drop in DLPC, IFC, PMC and SPC mirrors those fronto-parietal findings. The EC rise in PMC agrees with fMRI work showing that stronger premotor connectivity aids executive control.^47^ The LA decrease in the PMC indicates reduced local segregation in premotor regions, which may reflect altered support for executive and WM processes.^45^ Finally, the increased PC in DLPC and PMC matches reports that connector-hub reinforcement in these regions supports cognitive control under stress.^55,58^

### Heterogeneous fine-scale effects that cancel at the coarse scale

The third scenario involves heterogeneous fine-scale deviations yet showing no coarse-scale effect. This represents a cancellation mode, where competing fine-scale changes neutralize one another. Such balanced heterogeneity has been reported in previous works for temporo-parietal centrality and default-mode participation across multiple resolutions, suggesting a hidden tug of war that becomes invisible once neighbouring voxels are merged.^22,46,58^ In our data, this cancellation was evident for CC in IPC, EC in SPC and PC in IFC and IFOC, where mixed 76-node shifts summed to zero change at 24-node scale (Figs 3–5). These findings indicate that a coarse-scale null result may conceal meaningful micro-level imbalances that affect cognition. At the same time, cancellation may give the brain robustness, implying that if one region loses a long-range connection, an adjacent sub-parcel can compensate. Together, these scenarios underscore how multiscale connectomics can disentangle focal damage, distributed compensation and hidden equilibrium states, revealing subtle mechanisms that a single-scale approach would miss.

### Integrative interpretation

**Figure 6:**
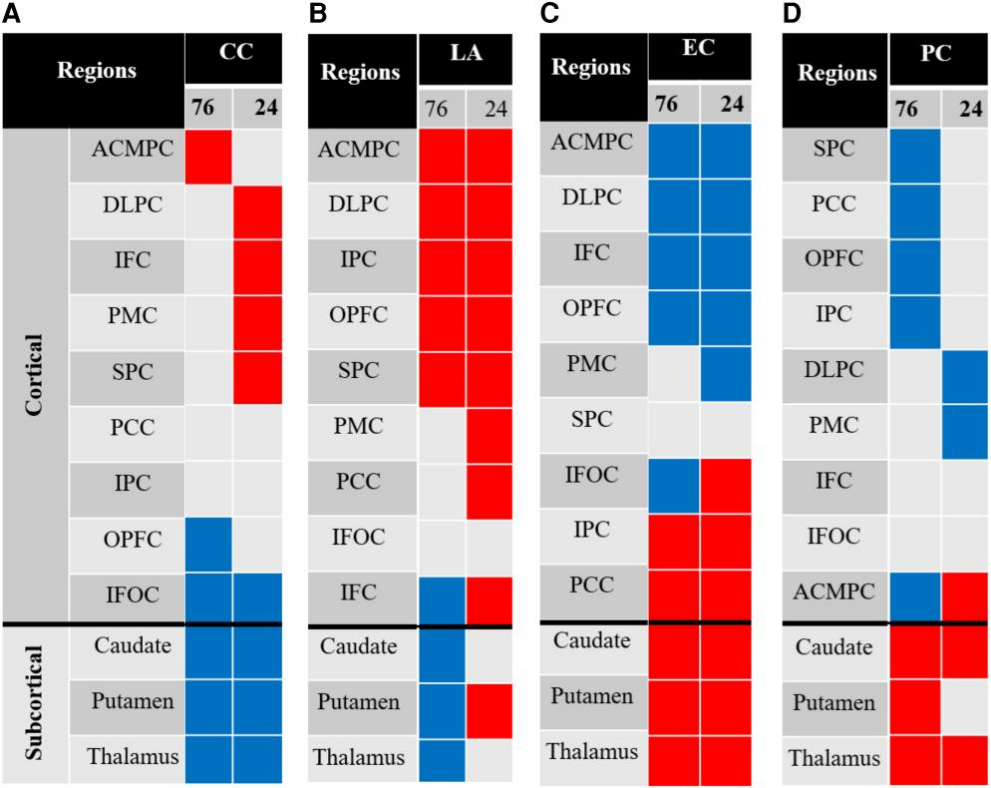
**Inverse segregation–integration patterns in working memory (WM) network graph metrics between ALL survivors and healthy controls (HC).** Colour-coded matrices summarize group differences across four local graph metrics at two spatial scales (76- and 24-node WM networks) for cortical and subcortical regions. Each panel corresponds to a network metric: (**A**) clustering coefficient (CC), (**B**) local assortativity (LA), (**C**) eigenvector centrality (EC) and (**D**) participation coefficient (PC). Regions shown in red indicate significantly reduced values in ALL survivors compared to HC, while blue indicates increased values in survivors. Grey represents regions with heterogeneous fine-scale effects or no fine-scale or coarse-scale effect. Inverse pattern illustrated between metrics of segregation (CC, LA) and integration (EC, PC), with subcortical regions showing increased segregation but reduced integration. In contrast, cortical regions show the opposite pattern: reduced segregation and increased integration. Group differences were assessed using multivariable linear regression with group (ALL versus HC) as the primary predictor and age and sex as covariates, with false discovery rate (FDR) correction applied for multiple comparisons (*P* < 0.05). The full set of regression estimates, raw *P*-values and FDR-corrected *P*-values for all nodes is provided in Supplementary Tables 5–12. The final sample included *N* = 70 ALL survivors and *N* = 70 HCs. Abbreviations: ACMPC, anterior cingulate and medial prefrontal cortex; DLPC, dorsolateral prefrontal cortex; IFC, inferior frontal cortex; IPC, inferior parietal cortex; IFOC, insular and frontal opercular cortex; OPFC, orbitofrontal cortex; PCC, posterior cingulate cortex; PMC, premotor cortex; SPC, superior parietal cortex.

Consolidating across metrics reveals a striking inverse interplay between measures of segregation such as CC and LA and measures of integration such as EC and PC. Conceptually, such segregation and integration trade-offs are a common motif in large-scale brain organization, where increases in CC and LA are often accompanied by reduced connector-hub status and vice versa.^37,59^ This pattern is illustrated in Fig. 6, showing red colour for regions with reduced metric value in ALL survivors as compared to corresponding values in HC, and blue for increased values in survivors. Regions showing heterogeneous fine-scale deviations and no fine-scale or coarse-scale effect are coloured in grey.

In subcortical nuclei, we could see a consistent increase in both CC and LA (Fig. 6A, B) while EC and PC (Fig. 6C, D) decreased, depicting circuits that are becoming internally cohesive yet progressively disconnected from broader cortical exchange. Biologically, this pattern suggests subcortical gating whereby motor-reward loops tighten their local processing at the expense of cross-network dialogue.^42,45,49,53^ Clinically, similar subcortical over-segregation has been linked to bradyphrenia and slowed executive throughput in ALL survivors and other frontal subcortical disorders.^44^

Likewise, inverse patterns could be seen in cortical regions too. This involves decrease in CC and LA whereas EC and PC increase with reduced CC in ACMPC, DLPC, IFC, PMC and SPC (Fig. 6A), reduced LA in ACMPC, DLPC, OPFC, IPC, SPC, PMC and PCC (Fig. 6B), increased EC in ACMPC, DLPC, OPFC, IFC and PMC (Fig. 6C) and increased PC in DLPC, OPFC, IPC, PMC, PCC and SPC (Fig. 6D). Here, the cortex appears to sacrifice local clustering to amplify long-range hubness and cross-module participation, an adaptive topology previously interpreted as compensatory up-regulation of fronto-parietal control hubs under structural or metabolic stress.^47,55,58^ Our results align with the segregation–integration trade-off outlined by Shine *et al.*,^60^ whereby subcortical nodes retreat into specialized enclaves while cortical hubs broaden their influence, a coordinated shift that is likely fundamental to the neurocognitive phenotype observed in ALL survivors.

### Limitations and future directions

Despite the strengths of this study, including the use of multiscale graph theoretical analysis, functionally defined WM networks and a matched control group with comparable age and sex distribution, several limitations should be acknowledged. First, the cross-sectional design limits our ability to infer longitudinal progression or causal relationships between treatment exposure and observed network alterations. Longitudinal studies are needed to track how structural connectivity evolves over time in ALL survivors. Second, although diffusion MRI and tractography provide valuable estimates of white matter pathways, they are indirect and susceptible to methodological biases, such as false positives in streamline reconstruction and partial volume effects in regions of complex fibre geometry. Third, our structural connectomes were based on streamline count, which, while widely used, does not fully account for connection strength or microstructural integrity.

Fourth, because the HCP-MMP1 atlas is derived from adult multimodal data, its use in a paediatric cohort may introduce developmental mismatches in cortical boundaries. Paediatric-specific multimodal diffusion MRI parcellations remain less established.^61,62^ However, HCP-MMP1 has been widely used in both adult and developmental datasets, including large paediatric studies such as ABCD and HCP-D, demonstrating its stability and suitability for mixed-age structural analyses.^63,64^ Future work incorporating paediatric optimized parcellations may further refine WM-associated network definitions. Fifth, WM behavioural measures were not available in this cohort. As a result, direct structure–function associations could not be assessed. Confirming whether the observed network alterations relate uniquely to WM function will require harmonized cognitive assessments in future studies. Finally, our analysis focused specifically on WM-associated regions, and we did not examine whole-brain topology. Broader disruptions may coexist, and future work incorporating whole-brain multiscale analysis will be important for evaluating the relative specificity of WM effects. Future work integrating diffusion and fMRI, along with cognitive assessments and longitudinal follow-up, will be crucial for translating these findings into clinically meaningful biomarkers for cognitive outcomes in paediatric cancer survivors and to guide interventions for cognitive rehabilitation in childhood cancer survivors.

## Conclusion

Our framework gives researchers a consistent, task-defined map of WM network with a reproducible starting point for WM studies. Methodologically, our work highlights the added value of hierarchical parcellations and metric-specific insights for disentangling complex brain reorganization. We demonstrated these benefits in childhood ALL survivors across both scales, characterized by a reciprocal shift towards cortical integration and subcortical segregation. By demonstrating that key segregation and integration metrics invert in opposing anatomical domains and that these alterations are partly scale-dependent, we show that coarse-grained summaries alone cannot capture the nuanced re-balancing that underpins post-treatment neurocognitive profiles. The consistency of several findings across both 76- and 24-node scales underscores their robustness and nominates the fronto-parietal executive hubs, and basal-ganglia/thalamic nuclei as core nodes for future prognostic and therapeutic targeting. Clinically, these network fingerprints hold promise as biomarkers for early detection of cognitive vulnerability and for monitoring response to rehabilitation or neuromodulation strategies.

## Supplementary Material

fcag137_Supplementary_Data

## Data Availability

The imaging datasets analysed in this study were obtained from the St. Jude Total Therapy Study 16 cohort and the Human Connectome Project—Development (HCP-D). Access to Total Therapy Study 16 data is restricted and may be requested from St. Jude Children’s Research Hospital, subject to appropriate approvals. The HCP-D dataset is publicly accessible at https://www.humanconnectome.org/study/hcp-lifespan-development/document/hcp-development-20-release. All diffusion MRI preprocessing and structural connectivity reconstruction were performed using publicly available software, including FSL (https://fsl.fmrib.ox.ac.uk/fsl/docs/diffusion/index.html), MRtrix3 (https://www.mrtrix.org/), ANTs (https://github.com/ANTsX/ANTs) and FreeSurfer (https://github.com/freesurfer/freesurfer). Graph-theoretical analyses were conducted using Python libraries such as Nilearn (https://github.com/nilearn/nilearn) and NetworkX (https://github.com/networkx/networkx). Statistical analyses were performed in R (https://www.r-project.org/). Network visualizations and figure generation were produced using Nilearn and Connectome Workbench (https://github.com/Washington-University/workbench). Custom analysis scripts used in this study are available at https://github.com/rajikha/MultiscaleConnectivityFramework.
