# Supplementary material for: Multiscale connectivity framework for working memory network in paediatric acute lymphoblastic leukaemia survivors
Source: Brain Commun. 2026 Apr 17;8(2):fcag137. doi: 10.1093/braincomms/fcag137 (PMC13126661; doi:10.1093/braincomms/fcag137)
Supplement: fcag137_Supplementary_Data [file fcag137_supplementary_data.zip › Original_submission_manuscript.pdf]

OXFORD UNIVERSITY PRESS | Brain Communications

Multiscale Connectivity Framework for Working Memory Network in Pediatric Acute Lymphoblastic Leukemia Survivors

Journal:	*Brain Communications*
Manuscript ID	BRAINCOM-2025-1002
Manuscript Type:	Original Article
Date Submitted by the Author:	30-Sep-2025
Complete List of Authors:	Raja, Rajikha; St Jude Children's Research Hospital, Department of Radiology<br>Glass, John; St Jude Children's Research Hospital, Department of Radiology<br>Song, Ruitian; St Jude Children's Research Hospital, Department of Radiology<br>Jacola, Lisa; St Jude Children's Research Hospital, Department of Psychology and Biobehavioral Sciences<br>Patni, Tushar; St Jude Children's Research Hospital, Department of Biostatistics<br>Li, Yimei; St Jude Children's Research Hospital, Department of Biostatistics<br>Reddick, Wilburn; St Jude Children's Research Hospital, Department of Radiology
Keywords:	Acute Lymphoblastic Leukemia, structural connectivity, working memory, pediatric cancer survivors, graph network metrics

SCHOLARONE™
Manuscripts

Multiscale Connectivity Framework for Working Memory Network in Pediatric Acute Lymphoblastic Leukemia Survivors

Rajikha Raja,[1] John O. Glass,[1] Ruitian Song,[1] Lisa M. Jacola,[2] Tushar Patni,[3] Yimei Li,[3] Wilburn E. Reddick[1]

1 Department of Radiology, St. Jude Children's Research Hospital, Memphis, TN 38105, USA

2 Department of Psychology and Biobehavioral Sciences, St. Jude Children's Research Hospital, Memphis, TN 38105, USA

3 Department of Biostatistics, St. Jude Children's Research Hospital, Memphis, TN 38105, USA

Correspondence to: Rajikha Raja

Department of Radiology

St. Jude Children's Research Hospital,

262 Danny Thomas Pl, Memphis, TN 38105, USA

E-mail: rajikha.raja@stjude.org

For Review Only

Keywords: Acute Lymphoblastic Leukemia; structural connectivity; working memory; pediatric cancer survivors; white matter

Abstract

Background:

Working memory (WM) impairments are a common late effect in survivors of childhood acute lymphoblastic leukemia (ALL), yet the structural network substrates of these difficulties remain poorly defined. Existing connectomic studies often rely on whole brain parcellations, overlooking WM specific circuitry and multiscale organization.

Methods:

We developed a multiscale structural connectivity framework to investigate WM specific networks using diffusion MRI and performed a cross-sectional study with 70 ALL survivors and 70 age and sex matched healthy controls. WM relevant regions were identified based on functional activation patterns, and structural connectomes were constructed at two spatial scales: a fine-scale 76-node network and coarser 24-node network derived from spatially contiguous, architecturally and functionally coherent regional groupings, as defined in the HCP-MMP1.0 atlas. Graph theoretical metrics, clustering coefficient, Eigenvector centrality, local assortativity, and participation coefficient were computed to assess local network topology. Group comparisons were conducted with false discovery rate correction for multiple comparisons.

Results:

Compared to healthy controls, ALL survivors exhibited marked topological shifts. Specifically, clustering and assortativity were increased in the caudate, putamen, and thalamus but decreased in the frontoparietal cortex. In contrast, centrality and participation showed the opposite pattern, signaling subcortical segregation and cortical hyperintegration. These effects were consistent across both spatial scales. Additional findings included scale-specific effects unique to the fine scale, as well as heterogeneous fine-scale patterns that resolved into consistent regional changes at the coarse scale. All effects remained significant after False discovery rate (FDR) correction, highlighting the robustness of the network reorganization.

Conclusion:

Our framework combining a targeted WM network with multiscale connectomic analysis proves its worth by revealing structural changes of WM circuitry in ALL survivors compared to healthy controls. The results show a broad reorganization, with weakened cortical networks and strengthened subcortical circuits, possibly as a form of compensation. These insights sharpen our understanding of treatment related structural network alterations and point to new targets for future studies of cognitive outcomes and rehabilitation.

For Review Only

Introduction

Working memory (WM) is a core cognitive system in the brain that enables the temporary storage and manipulation of information, and this short-term processing acts as the foundation for complex abilities such as learning, decision making, and problem solving.[1] Disruptions to WM can significantly impair an individual's academic, occupational, and social functioning and can have far reaching consequences for functional independence and quality of life.[2,3] WM impairments have been documented in various clinical populations, including childhood cancer survivors, who often face long term neurocognitive challenges because of their treatment.[4] As a result, significant research has focused on exploring the structural and functional organization of the brain, along with alterations related to WM and associated deficits.[5] Identifying the neural underpinnings of neurocognitive late effects in clinical populations can inform targets for interventions to improve outcomes.[6]

In the quest to understand the structural changes underlying WM deficits, a growing body of work has focused on identifying and characterizing WM related brain regions and networks.[7–9] The brain regions and networks associated with WM involve a distributed set of cortical and subcortical nodes intricately connected to support the encoding, maintenance, and retrieval of information.[5,10] Traditionally, these attempts have relied on task based functional MRI (fMRI) to pinpoint regions engaged during WM tasks such as N-back,[10,11] or on resting state functional connectivity analyses to highlight communities of regions working in concert.[12,13] However, inconsistencies arise from varied methodological choices, including different task contrasts, threshold settings, and brain parcellation schemes, resulting in divergent WM region definitions across studies.[14] These variations make it challenging to integrate functional insights into diffusion MRI connectomics or to reproduce and compare findings. To address this challenge, we derived and released a standardized list of 76 WM regions based on high-quality Human Connectome Project (HCP) task maps. This provides researchers with a reproducible framework for extracting a WM-specific sub-connectome, eliminating the need to perform additional fMRI analyses.

Survivors of acute lymphoblastic leukemia (ALL), for instance, frequently report persistent difficulties in attention, executive functioning, and WM.[4] Neuroimaging studies link these deficits to diffuse white matter injury, especially within fronto-parietal and basal-ganglia pathways.[15] Recent advances in neuroimaging and network science have highlighted the importance of structural connectivity for understanding the brain's functional architecture.[16–18] Kesler and colleagues reported that long-term ALL survivors show lower global efficiency and altered modular organization of the whole-brain connectome, changes that correlated with poorer executive performance.[19] While the field has seen widespread application of whole brain analyses, there is a growing need for approaches that focus on specific cognitive networks.[20,21] Fine grained examinations of WM related structural connectivity can reveal subtle alterations that remain obscured in global analyses.[22,23] The availability of high resolution cortical parcellations,[24,25] diffusion MRI tractography, and computational tools now make it feasible to systematically derive

For Review Only

WM targeted sub-connectivity matrices. Although numerous studies have applied connectomics to investigate structural and functional brain networks, few have zeroed in on establishing a framework to perform WM specific connectivity analysis.[26] Existing approaches that integrate WM related regions into structural network analyses often require multiple labor-intensive steps, subjective thresholds, or manual curation, making replication and comparison across studies difficult.[22] The development of a framework for isolating WM specific structural networks and enabling direct comparison of WM related structural connectivity across different populations, conditions, or interventions would improve the consistency and interpretability of findings.

The present work aims to address this gap by introducing a multiscale framework that integrates robust definitions of WM related regions with subsequent structural connectivity analysis. Multiscale analyses are widely used in network neuroscience via hierarchical or multi‑resolution parcellations, and it is well recognized that graph measures depend on scale and node definition.[14,23,27] Here we introduce a task‑defined WM network and a multiscale structural connectomics framework that quantifies graph network measure across two WM specific parcellation levels. By applying this framework to both healthy controls (HC) and ALL survivors, we demonstrate how it can uncover nuanced differences in WM related structural networks. Our overarching motivation is twofold: first, to develop a framework for WM network extraction and multiscale connectivity analysis that can be applied across studies, and second, to harness this method to gain clinically meaningful insights into how WM connectivity differs in pediatric ALL survivors, population that is vulnerable to treatment related neurocognitive difficulties. Ultimately, this approach can guide future research toward more targeted interventions that improve cognitive outcomes and quality of life for individuals impacted by ALL and other neurological conditions involving WM impairments.

Materials and Methods

Participants

Participants included in this study belong to St. Jude Total Therapy Study 16 cohort (ClinicalTrials.gov identifier NCT00549848) which enrolled 598 patients (aged 0–18 years at diagnosis) between 2007 and 2017 to study the treatment effects in pediatric ALL patients aiming to improve the cure rate of children with ALL. Detailed treatment protocols and primary outcomes of Total Therapy Study 16 have been reported previously.[28,29] All participants received risk directed chemotherapy under the protocol approved by the institutional review board, with written informed consent obtained from parents or legal guardians (and assent from minors where applicable). As part of the therapeutic protocol, patients were imaged at completion of treatment, approximately 2.5 years after diagnosis. Exclusion criteria for the current study included prior cranial radiation therapy for central nervous system (CNS) relapses, any history of relapses or secondary neoplasm, the presence of a genetic disorder known to affect cognition, or a significant history of head trauma or other neurological conditions unrelated to cancer therapy. From this

For Review Only

group, 97 participants were selected for this study based on the availability of advanced diffusion MRI data acquired with b-value of 1500 s/mm² and in 64 gradient directions. Clinical characteristics, including age at diagnosis and treatment risk arm, were obtained from study records for all included participants. Age and sex matched HC were included in the study with a tolerance of ±1 year for age bins and identical male-to-female ratios from the publicly available Human Connectome Project – Development (HCP-D) dataset[30,31] which enrolled 652 participants. Of these, 176 individuals with complete diffusion MRI data of sufficient quality were considered for this study. The HCP-D applied its own eligibility criteria, including exclusion of individuals with significant neurological or psychiatric disorders.

MRI Data Acquisition

All MRI data were acquired on a 3T Siemens Prisma scanner using a 64-channel head coil. Diffusion weighted imaging (DWI) data were collected using a single-shell spin-echo echo-planar imaging (EPI) sequence with the following parameters: 64 diffusion weighted directions with a b-value of 1500 s/mm² and one non-diffusion weighted (b = 0 s/mm²) volume. Sequence parameters were: repetition time (TR) = 4000 ms, echo time (TE) = 77.4 ms, voxel size = 1.8 × 1.8 × 1.8 mm³, and 108 contiguous axial slices covering the entire brain. Additionally, high-resolution 3D T1-weighted anatomical images were also acquired using a magnetization prepared rapid gradient echo (MPRAGE) sequence with TR = 1800 ms, TE = 2.26 ms, inversion time (TI) = 900 ms, flip angle = 9°, voxel size = 1.0 × 1.0 × 1.0 mm³. Diffusion MRI data from the HCP-D dataset were resampled to match the ALL acquisition protocol of b-value = 1500 s/mm² and 64 gradient directions.

Design of Multiscale Structural Connectivity Framework

We developed a targeted structural connectivity framework to isolate and analyze white matter pathways specifically associated with WM. Our framework was designed to analyze WM networks at two scales: a fine 76-node scale to capture subregional specificity, and a coarser 24-node scale to enable broader anatomical interpretation and cross-scale comparisons. This section outlines the key stages of the framework, including the identification of WM related regions, diffusion MRI processing, and the construction of WM specific structural connectomes.

WM Network

We begin with a recognized, standard atlas called, multimodal parcellation atlas of Human Connectome Project (HCP-MMP1)[25], which forms the anatomical basis of our WM node definitions. The rationale for selecting the HCP-MMP1 atlas was based on its multimodal integration, high spatial resolution, and neuroanatomical precision. Fig. 1 provides an overview of the WM network definition workflow, illustrating the process of narrowing from the whole-brain HCP-MMP1 atlas to a specific set of functionally defined WM regions. The atlas offers a high-resolution map of 180 bilateral cortical regions (Fig. 1A-C). Glasser et al. have published task-contrast maps (2BK-0BK) that highlight regions strongly activated during a WM task. The

For Review Only

availability of the 2BK-0BK task contrast within this atlas provided an added advantage due to the functional specificity of these contrast maps in accurately identifying WM regions. Transitioning from manual inspection of these activation maps to generate WM node set is the key idea of this framework. This step involves a careful, manual review of the 2BK–0BK maps to verify which HCP-MMP1 regions exhibit robust WM related activation. The aim is to retain nodes that show consistent involvement in WM processes, while excluding areas with negligible or inconsistent activation (Fig. 1D-E). We identified cortical regions exhibiting strong activation in the 2BK-0BK contrast maps, resulting in a set of 76 WM related regions derived from the HCP-MMP1 atlas (Fig. 1F-H). These regions comprised 35 bilateral cortical regions and 3 bilateral subcortical regions. This initial node selection step ensures that the starting point of the framework is grounded in functionally validated, neuroanatomically precise data.

MRI Data Processing

For Review Only

Diffusion MRI data was processed using a custom pipeline comprising four main stages: preprocessing, reconstruction, tractography, and connectome matrix generation. Initially, raw diffusion weighted images were corrected for artifacts including eddy current induced distortions, susceptibility related distortions, and subject motion using FSL's *eddy* and *topup* tools.[32] Preprocessed diffusion data were then reconstructed using the constrained spherical deconvolution (CSD) model in MRtrix3 to estimate fiber orientation distribution (FOD) functions.[33,34] This model enables accurate representation of multiple fiber populations within a voxel, supporting reliable tractography in regions with complex fiber architecture. As a next step, whole brain probabilistic tractography was performed using the iFOD2 algorithm in MRtrix3 on the reconstructed FOD images.[35] An initial tractogram containing 20 million streamlines was generated to ensure comprehensive coverage of white matter pathways. This tractogram was then refined using Spherical deconvolution Informed Filtering of Tractograms (SIFT2) to improve biological accuracy by reducing biases in streamline density.[36] The final tractogram consisted of 200,000 streamlines, each assigned a weight based on SIFT2 optimization. Finally, structural connectomes were derived from the SIFT2 weighted tractograms using the HCP-MMP1.0 parcellation, which delineates 379 cortical and subcortical brain regions. The parcellation image was registered to each subject's diffusion space, and streamlines were assigned to region pairs. Connectivity matrices of size 379 × 379 were constructed using MRtrix3, where edge weights reflect the total SIFT2 assigned streamline weights between region pairs (Fig. 2A-B). To account for variability in region size and reduce the influence of node volume on connection strength, connectivity values were further normalized based on the inverse of the anatomical region volumes.

WM Structural Connectivity

WM structural connectivity matrices were constructed following a two-step hierarchical approach to analyze the connectomes at two spatial scales by systematically reducing the whole brain connectivity matrix to WM relevant subgraphs. Once the WM related nodes were defined, we extracted their corresponding structural connectivity profiles from the whole brain diffusion

MRI based connectomes. Each node served as a region within the WM network, and edges represented the reconstructed white matter pathways linking these WM nodes. By focusing our connectivity estimation on these functionally identified WM regions, we constructed a sub-connectivity matrix that emphasizes structural relationships relevant to WM processing, filtering out extraneous network connections not central to this cognitive domain (Fig. 2C-D).

The next step is anatomical grouping of WM nodes to facilitate higher level interpretation of connectivity patterns. We grouped the identified WM related nodes into broader cortical clusters. This aggregation was based on the multimodal cortical parcellation scheme defined in[25], in which the 180 bilateral cortical areas are organized into 22 neuroanatomically and functionally coherent broad regions. We adopted this scheme to assign the 35 bilateral WM related cortical parcels to 9 bilateral cortical categories, which are anterior cingulate and medial prefrontal cortex (ACMPC), dorsolateral prefrontal cortex (DLPC), inferior frontal cortex (IFC), insular and frontal opercular cortex (IFOC), inferior parietal cortex (IPC), orbital and polar frontal cortex (OPFC), posterior cingulate cortex (PCC), premotor cortex (PMC), superior parietal cortex (SPC) as listed in Table 1. Additionally, 3 bilateral subcortical regions (thalamus, caudate, and putamen) were retained as distinct parcels, yielding a final set of 24 anatomically defined WM regions.

For Review Only

To compute the 24 × 24 coarse-scale connectivity matrix, we aggregated the corresponding entries from the 76 × 76 fine scale matrix (Fig. 2E-F). Specifically, for each pair of coarse scale regions, all interconnections between their constituent fine scale nodes were identified, and the corresponding streamline weights were summed to form the edge weight in the coarse matrix. This procedure was applied symmetrically across rows and columns, ensuring that the coarse-scale matrix preserved total within and between group connectivity. This computation is visually demonstrated in Fig. 2, for computing the connection strength for the edge connecting left of IPC and SPC, where the zoomed inset in Fig. 2D highlights the fine-scale matrix entries used to derive the single coarse-scale edge shown in Fig. 2F. This summation-based approach retained the strength and distribution of connections while reducing dimensionality. Presenting the WM network at both node-level and the aggregated coarser-level, preserves node level resolution and supports higher level interpretation of connectivity patterns across major anatomical systems.

Local Graph Network Metrics Computation

To characterize the topological organization of the WM structural networks, we computed a set of local graph metrics for each node within the defined WM subnetwork. These metrics were chosen to capture complementary aspects of nodal connectivity and integration within the broader network architecture.[37] Specifically, we computed four local metrics: clustering coefficient (CC), Eigenvector centrality (EC), local assortativity (LA), and participation coefficient (PC). Among these, CC and LA are primarily considered measures of local integration and structural organization, whereas EC and PC reflect the nodal influence and inter-modular communication within the global network.

The CC was used to quantify the extent to which a node's neighbors are themselves interconnected, providing a measure of local connectivity density. To assess the global importance of each node, we computed EC, which accounts not only for a node's direct connections but also for the centrality of its neighbors. The LA metric was included to evaluate the extent to which a node tends to connect with others of similar degree or strength, offering insight into local structural properties. Lastly, the PC was calculated to determine how evenly a node's connections are distributed across different network modules, indicating its role in facilitating cross-network communication. To aid interpretation, we provide a summary of the four local graph metrics in Supplementary Table 1. All metrics were computed using NetworkX, a Python-based graph analysis library,[38] applied to weighted, undirected connectivity matrices. Metric computation was performed for each subject individually at both the 76-node and 24-node WM network scales.

Statistical Analysis

To assess group differences in WM structural connectivity, we conducted statistical analysis on local graph network metrics derived from the WM structural connectomes. Analyses were performed at both the 76-node and 24-node scales. A multivariable linear regression model was used to compare ALL survivors and demographically matched HC and heteroskedasticity consistent robust standard errors were employed to ensure valid statistical inference. For each local graph metric and each node, the model included group ALL vs. HC as the primary predictor, with age and sex included as covariates to control for potential confounding effects. False discovery rate (FDR) correction was applied to the resulting p-values across nodes to account for multiple comparisons, using the Benjamini-Hochberg procedure.[39] with a significance threshold of FDR corrected $p < 0.05$. All statistical analyses were conducted using R statistical software (version 4.4.2). The analysis utilized a complete dataset, eliminating the need for any data imputation procedures.

Results

Participant Characteristics

From the initial 97 ALL survivors and 176 healthy controls (HCP-D) considered for this study, 70 participants were selected from each group after age and sex matching. This matching ensured comparable male-to-female ratios and similar age distributions across groups. The demographic profile of the final ALL cohort was comparable to the overall Total Therapy Study 16 population, supporting the representativeness of this subgroup. The final study sample therefore included 140 participants (70 ALL survivors and 70 HC). Participant demographics and clinical characteristics are summarized in Table 2. The difference in age between groups was not statistically significant ($p = 0.09$). Age and sex were included as covariates in all subsequent statistical analyses.

Group Differences in Local Graph Metrics at the 76-Node Scale

For Review Only

The results for each network metric are illustrated using graphical visualizations comprising glass brain plots, groupwise boxplots, and mean difference bar plots. As an exemplar, Fig. 3 presents these plots for CC at the 76-node scale. The glass brain plots highlight the spatial distribution of significant nodes, providing anatomical context and revealing the localization and hemispheric patterning of altered connectivity across cortical and subcortical structures. The accompanying boxplots illustrate the distribution of metric values within each group, while the mean difference bar plots show raw group differences (ALL − HC) ranked to emphasize regions with the most prominent effects. Consolidated summaries of additional metrics at the 76-node and 24-node scales are provided in Fig. 4 and Fig. 5, respectively, while full per-metric visualizations are available in Supplementary Figures, and detailed statistical results are presented in Supplementary Tables.

Clustering Coefficient

Group differences in CC between ALL survivors and HC are presented in Fig. 3 and detailed in Supplementary Table 2. Regions with significantly reduced clustering in the ALL group (ALL < HC) are shown in Fig. 3A–C, while regions with increased clustering (ALL > HC) are shown in Fig. 3D–F. Regions with significantly reduced clustering in the ALL group (ALL < HC) include ACMPC, DLPC, IFC, IPC, PMC, and SPC (Fig. 3A). These differences are further illustrated in the corresponding boxplots and mean difference bar plots, showing clear group separation (Fig. 3B–C). The most pronounced decreases are observed in SPC, PMC, and DLPC. In contrast, regions with increased clustering in the ALL group (ALL > HC) include cortical regions such as OPFC, and IFOC and subcortical regions (caudate, thalamus, and putamen) (Fig. 3D), with the strongest increases observed in ventral frontal and subcortical regions.

Eigenvector Centrality

Summarized group differences in EC at the 76-node scale are shown in Fig. 4A–B, with full visualizations in Supplementary Fig. 1 and statistical details in Supplementary Table 3. Significant reductions in the ALL group were found in the cortical regions such as IPC, PCC, PMC, and SPC and subcortical regions (caudate, thalamus, and putamen) (Fig. 4A; Supplementary Fig. 1A). These reductions were particularly pronounced in posterior cortical and subcortical regions. Conversely, increased EC in the ALL group was observed in the cortical (ACMPC, DLPC, IFC, IFOC, OPFC, PMC, and SPC) brain regions (Fig. 4B; Supplementary Fig. 1D). Elevated centrality was most prominent in prefrontal, parietal, and associative cortical areas, as shown by the distribution plots and mean difference bars (Supplementary Fig. 1).

Local Assortativity

Summarized group differences in LA are shown in Fig. 4C–D, with full visualizations in Supplementary Fig. 2 and statistical details in Supplementary Table 4. Regions with significantly reduced assortativity in the ALL group (ALL < HC) are shown in Fig. 4C and Supplementary Fig. 2A–C, while regions with increased assortativity (ALL > HC) are shown in Fig. 4D and

For Review Only

Supplementary Fig. 2D–F. The ALL group exhibited significantly reduced LA in several higher-order cortical areas, including the ACMPC, DLPC, IPC, OPFC, PMC, and SPC. These reductions were most prominent in SPC and IPC. In contrast, increased LA in the ALL group was detected in the cortical (IFC, and PMC) and subcortical (caudate, putamen, and thalamus) brain regions with the most substantial increases occurring in subcortical regions.

Participation Coefficient

Summarized group differences in PC are shown in Fig. 4E–F, with full visualizations in Supplementary Fig. 3 and statistical details in Supplementary Table 5. Regions with significantly reduced participation in the ALL group (ALL < HC) are shown in Fig. 4E and Supplementary Fig. 3A–C, while regions with increased participation (ALL > HC) are shown in Fig. 4F and Supplementary Fig. 3D–F. Reduced participation in the ALL group was found cortical (DLPC, IFC, IFOC, PMC) and subcortical (caudate, putamen, and thalamus) brain regions with the most notable decreases observed in subcortical areas. On the other hand, increased participation was observed in the ACMPC, DLPC, IFC, IPC, IFOC, OPFC, PCC, PMC, and SPC, primarily involving cortical association regions.

For Review Only

Group Differences in Local Graph Metrics at the 24-Node Scale

Clustering Coefficient

At the coarser 24-node scale, group differences in CC are summarized in Fig. 5A–B, with full visualizations in Supplementary Fig. 4 and statistical details in Supplementary Table 6. Regions with significantly reduced clustering in the ALL group (ALL < HC) are shown in Fig. 5A and Supplementary Fig. 4A–C, while those with increased clustering (ALL > HC) are shown in Fig. 5B and Supplementary Fig. 4D–F. The ALL group demonstrated significant reductions in clustering in regions including the DLPC, IFC, PMC, and SPC, with the most marked decreases in PMC and DLPC. In contrast, elevated clustering in the ALL group was observed in IFOC and subcortical regions including the caudate, thalamus, and putamen. The largest increases were noted in the thalamus and caudate.

Eigenvector Centrality

Group differences in EC at the 24-node scale are summarized in Fig. 5C–D, with full visualizations in Supplementary Fig. 5 and statistical details in Supplementary Table 7. Regions with significantly reduced centrality in the ALL group (ALL < HC) are shown in Fig. 5C and Supplementary Fig. 5A–C, while regions with increased centrality (ALL > HC) are shown in Fig. 5D and Supplementary Fig. 5D–F. The ALL group exhibited significantly reduced centrality in the cortical (IPC, PCC, and IFOC) and subcortical (caudate, thalamus, putamen) regions, with the strongest effects observed in the thalamus, caudate, and putamen. Conversely, increased centrality in the ALL group was identified in the ACMPC, DLPC, IFC, OPFC, and PMC, with the most pronounced effects in the PMC and DLPC.

Local Assortativity

Group differences in LA on the 24-node scale are summarized in Fig. 5E, with full visualizations in Supplementary Fig. 6 and statistical details in Supplementary Table 8. Decreased assortativity in the ALL group involved both cortical (ACMPC, DLPC, IFC, IPC, OPFC, PCC, PMC, and SPC) and subcortical (putamen) regions. The most pronounced reductions were observed in SPC, PCC, and OPFC.

Participation Coefficient

Group differences in PC at the 24-node scale are summarized in Fig. 5F–G, with full visualizations in Supplementary Fig. 7 and statistical details in Supplementary Table 9. Regions with significantly reduced participation in the ALL group (ALL < HC) are shown in Fig. 5F and Supplementary Fig. 7A, while regions with increased participation (ALL > HC) are shown in Fig. 5G and Supplementary Fig. 7B. The ALL group showed reduced participation in the ACMPC and subcortical (caudate, and thalamus) with the most marked decreases observed in the thalamus and caudate. In contrast, increased participation was noted in the DLPC and PMC. Mean difference bar plots highlighted negative group differences in subcortical regions and positive differences in frontal-parietal areas.

Discussion

This study provides a comprehensive characterization of brain regions commonly associated with WM at the end of protocol-directed chemotherapy treatment for childhood ALL, using a multiscale graph theoretical approach to analyze structural connectivity derived from diffusion MRI. By examining local graph metrics at both a fine grained 76-node and a coarser 24-node WM network scale, we identified consistent and spatially distributed disruptions in topological organization across multiple network dimensions, including local clustering, hubness, assortativity, and inter-modular integration. The findings underscore the importance of a multiscale analytical framework to fully capture the complexity of brain network dynamics. We noted a spectrum of multiscale response patterns across all the four graph metrics, from uniform increases or decreases that appeared at both resolutions, to alterations confined to the fine 76‑node scale, and even heterogeneous fine‑scale shifts that either amplified or vanished when viewed at the coarser 24‑node level. In the paragraphs that follow we discuss these scenarios for each metric, showing how the spatial scale shapes their topological and functional implications. The observed differences in WM network organization between ALL survivors and controls are suggestive of treatment and diagnosis related effects. However, without explicit analyses linking imaging findings to treatment exposures, these observations should be interpreted cautiously. Future studies integrating treatment variables will be essential for establishing direct associations and clarifying mechanisms.

Consistent cross‑scale patterns

For Review Only

Increased CC in ALL survivors compared to HCs could be seen consistently across 76 and 24 scales in regions such as IFOC, caudate, putamen, and thalamus (Fig. 3D and 5B). This indicates tighter, highly segregated neighborhoods within salience processing and cortico-striatal-thalamic motor loops.[37,40,41] Crucially, similar over-segregation in these nodes has been linked to poorer WM performance in childhood ALL survivors and other cohorts.[42,43] Increased EC in ALL survivors could be seen consistently across 76 and 24 scales in executive frontal areas such as ACMPC, DLPC, IFC, OPFC but decreased in the PMC and the three basal-ganglia/thalamic nuclei (Fig. 4B and 5D). These frontal regions overlap the canonical, highly reliable hubs mapped by,[44] indicating a systematic but not random shift. Echoing Cole et. al.,[45] who tied stronger prefrontal hubness to better cognitive control, our findings suggest survivors reroute network influence from motor-subcortical loops to executive hubs as a compensatory strategy to preserve cognition after treatment. The fronto-subcortical EC imbalances we observed align with prior reports of poorer WM performance in long-term ALL survivors[42,46] and with mechanistic basal-ganglia gating studies.[43,47] Together, these findings support the idea that EC alterations contribute to the WM deficits characteristic of this population.

Compared to HC, survivors of ALL showed decreased LA in both scales in several fronto-parietal regions such as ACMPC, DLPC, IPC, OPFC, and SPC (Fig. 4C and 5E). According to prior studies,[37,48] lower LA in key nodes means they stop favoring similar partners, removing the network's usual barriers against random crosstalk. Because the same hubs form the fronto-parietal control network,[13,49] this crosstalk overload likely destabilizes the circuits that support attention and WM in ALL survivors. Finally, PC consistently declined in the caudate and thalamus (Fig. 4E and 5F), denoting a stable retreat of these nodes from cross-modular communication and consonant with their EC demotion. Because PC quantifies the extent to which a node's links spans multiple modules,[37,50] lower values in the caudate and thalamus point to a stable loss of their normal integrative, hub-like role.[40,51] Reductions in striatal and thalamic integration have been tied to poorer WM and attention outcomes in childhood ALL survivors,[42,46] supporting the functional significance of the PC decline observed here. Collectively, these consistent changes across scales may represent true distributed effects tied to cognitive dysfunction such as cortical thinning and white matter degradation. These regions likely represent core hubs of pathological or compensatory reorganization in ALL survivors. The consistency across scales reinforces their role as key drivers of WM network alteration and suggests that both local and system level architecture is affected in these areas.

Scale-specific and heterogeneous patterns

A critical strength of this study lies in the use of hierarchical anatomical modeling, which exposed a far broader spectrum of scale-dependent response patterns beyond the consistent alterations that would be obscured in any single-resolution analysis. We identified several distinct multiscale scenarios, each offering unique neurobiological implications. The first scenario involves effects that appeared only at the 76-node scale suggest highly focal or subregional

For Review Only

changes such as micro-circuit pruning, early demyelination, or small-scale compensatory sprouting that are too spatially constrained to sway the coarser parcellation. Previous scale-sensitive graph works demonstrated that regional effects can disappear when parcels are spatially averaged.[14,23] In our cohort these purely fine-scale effects comprised increased CC in OPFC and decreased CC in ACMPC, increased LA in the caudate and thalamus, and increased PC in OPFC, IPC, PCC and SPC.

Increased CC in ALL survivors observed in the OPFC indicates a focal tightening of orbitofrontal neighbourhoods that does not propagate to larger functional modules (Fig. 3). Orbitofrontal micro-circuits are known to be especially locally interconnected.[52] Moreover, a diffusion-MRI study of long-term childhood ALL survivors reported increased local segregation in orbitofrontal white matter regions alongside executive deficits,[46] supporting the functional relevance of the OPFC CC rise observed here. Conversely, decreased CC seen in the ACMPC, indicate subtle local fragmentation of cingulo-medial networks that remains undetected when nodes are pooled (Fig. 3). The ACMPC normally exhibits high local clustering to support conflict monitoring and WM updating.[13,53] Studies in pediatric cohorts including childhood ALL survivors show that reduced anterior cingulate clustering or local efficiency predicts poorer attention and WM performance.[42,54]

The second scenario features heterogeneous changes at the 76-node level that, when pooled into 24-node parcels, sum to an overall increase or decrease. This indicates a meso-scale reallocation in which enough sub-parcels shift in the same direction to push the whole region toward hyper-integration, marked by higher PC or EC, or toward hypo-segregation, marked by lower CC or LA. Previous studies have illustrated this scenario as biased-averaging phenomenon showing direction-reversals across resolutions in diffusion and fMRI graphs in premotor and parietal hubs.[55] In our study this effect appeared in several regions such as heterogenous CC in DLPC, IFC, PMC and SPC with net coarse-scale reduction (Fig. 3 and 5A), heterogenous EC in PMC with net coarse-scale increase (Fig. 4A-B and 5D), heterogenous LA in PMC with net coarse-scale reduction (Fig. 4C-D and 5E), and heterogenous PC in DLPC and PMC with net coarse-scale increase (Fig. 4E-F and 5G).

Reduced segregation and greater integration can both hurt WM in ALL survivors. Structural MRI from the St Jude Lifetime Cohort shows that lower clustering and local efficiency in fronto-parietal tracts predict later WM problems.[42] Our coarse-scale CC drop in DLPC, IFC, PMC, and SPC mirror those fronto-parietal findings. The EC rise in PMC agrees with fMRI work showing that stronger premotor connectivity aids executive control.[45] The LA decrease in the PMC indicates reduced local segregation in premotor regions, which may reflect altered support for executive and WM processes.[43] Finally, the increased PC in DLPC and PMC matches reports that connector-hub reinforcement in these regions supports cognitive control under stress.[53,56]

The third scenario involves heterogeneous fine-scale deviations yet showing no coarse-scale effect. This represents a cancellation mode, where competing fine scale changes neutralize

For Review Only

one another. Such balanced heterogeneity has been reported in previous works for temporo-parietal centrality and default-mode participation across multiple resolutions suggesting a hidden tug of war that becomes invisible once neighboring voxels are merged.[22,44,56] In our data, this cancellation was evident for CC in IPC, EC in SPC, and PC in IFC and IFOC, where mixed 76-node shifts summed to zero change at 24-node scale (Fig. 3,4 and 5). These findings indicate that a coarse scale null result may conceal meaningful micro-level imbalances that affect cognition. At the same time, cancellation may give the brain robustness implying if one region loses long-range connection, an adjacent sub-parcel can compensate. Together, these scenarios underscore how multiscale connectomics can disentangle focal damage, distributed compensation, and hidden equilibrium states revealing subtle mechanisms that a single-scale approach would miss.

Integrative interpretation

Consolidating across metrics reveals a striking inverse interplay between measures of segregation such as CC and LA and measures of integration such as EC and PC. Conceptually, such segregation and integration trade-offs are a common motif in large-scale brain organization, where increases in CC and LA are often accompanied by reduced connector-hub status and vice-versa.[37,57] This pattern is illustrated in Fig. 6 showing red color for regions with reduced metric value in ALL survivors as compared to corresponding values in HC and blue for increased values in survivors. Regions showing heterogeneous fine-scale deviations and no fine-scale or coarse-scale effect are colored in gray.

In subcortical nuclei, we could see consistent increase in both CC and LA (Fig. 6A-B) while EC and PC (Fig. 6C-D) decrease, depicting circuits that are becoming internally cohesive yet progressively disconnected from broader cortical exchange. Biologically, this pattern suggests subcortical gating whereby motor‑reward loops tighten their local processing at the expense of cross‑network dialogue.[40,43,47,51] Clinically, similar subcortical over-segregation has been linked to bradyphrenia and slowed executive throughput in ALL survivors and other frontal subcortical disorders.[42]

Likewise, inverse patterns could be seen in cortical regions too. This involves decrease in CC and LA whereas EC and PC increase with reduced CC in ACMPC, DLPC, IFC, PMC, and SPC (Fig. 6A), reduced LA in ACMPC, DLPC, OPFC, IPC, SPC, PMC, and PCC (Fig. 6A-B), increased EC in ACMPC, DLPC, OPFC, IFC, and PMC (Fig. 6C) and increased PC in DLPC, OPFC, IPC, PMC, PCC, and SPC (Fig. 6D). Here, the cortex appears to sacrifice local clustering to amplify long‑range hubness and cross‑module participation, an adaptive topology previously interpreted as compensatory up-regulation of fronto-parietal control hubs under structural or metabolic stress.[45,53,56] Our results align with the segregation–integration trade-off outlined by Shine et. al.,[58] whereby subcortical nodes retreat into specialized enclaves while cortical hubs broaden their influence, a coordinated shift that is likely fundamental to the neurocognitive phenotype observed in ALL survivors.

For Review Only

Limitations and future directions

Despite the strengths of this study, including the use of multiscale graph theoretical analysis, functionally defined WM networks, and a matched control group with comparable age and sex distribution, several limitations should be acknowledged. First, the cross-sectional design limits our ability to infer longitudinal progression or causal relationships between treatment exposure and observed network alterations. Longitudinal studies are needed to track how structural connectivity evolves over time in ALL survivors. Second, although diffusion MRI and tractography provide valuable estimates of white matter pathways, they are indirect and susceptible to methodological biases, such as false positives in streamline reconstruction and partial volume effects in regions of complex fiber geometry. Third, our structural connectomes were based on streamline count, which, while widely used, does not fully account for connection strength or microstructural integrity. Fourth, while we defined WM relevant regions using robust task-based fMRI contrasts from the HCP atlas, we did not attempt to utilize functional or cognitive performance data from our own cohort. Given that WM impairment is expected only in a subset of patients, and varies with treatment exposure, age, and other clinical factors, this limits direct interpretation of structural alterations to functional outcomes. Future work integrating diffusion and fMRI, along with cognitive assessments and longitudinal follow up, will be crucial for translating these findings into clinically meaningful biomarkers for cognitive outcomes in pediatric cancer survivors and to guide interventions for cognitive rehabilitation in childhood cancer survivors.

Conclusion

Our framework gives researchers a consistent, task-defined map of WM network with a reproducible starting point for WM studies. Methodologically, our work highlights the added value of hierarchical parcellations and metric-specific insights for disentangling complex brain reorganization. We demonstrated these benefits in childhood ALL survivors across both scales, characterized by a reciprocal shift toward cortical integration and subcortical segregation. By demonstrating that key segregation and integration metrics invert in opposing anatomical domains and that these alterations are partly scale-dependent, we show that coarse-grained summaries alone cannot capture the nuanced re-balancing that underpins post-treatment neurocognitive profiles. The consistency of several findings across both 76- and 24-node scales underscores their robustness and nominates the fronto-parietal executive hubs, and basal-ganglia/thalamic nuclei as core nodes for future prognostic and therapeutic targeting. Clinically, these network fingerprints hold promise as biomarkers for early detection of cognitive vulnerability and for monitoring response to rehabilitation or neuromodulation strategies.

For Review Only

Data availability

The data that support the findings of this study are available from the corresponding author, upon reasonable request.

Acknowledgments

The authors wish to acknowledge the patients and families that volunteered their time for the study. The authors thank the Human Connectome Project–Development (HCP-D) consortium for providing publicly available imaging data. Data were provided in part by the Human Connectome Project, WU-Minn Consortium (Principal Investigators: David Van Essen and Kamil Ugurbil; 1U54MH091657) funded by the 16 NIH Institutes and Centers that support the NIH Blueprint for Neuroscience Research; and by the McDonnell Center for Systems Neuroscience at Washington University.

Funding

This research was supported in part by Cancer Center Support Grant P30 CA21765 and R01 CA90246 (WER) from the National Cancer Institute and ALSAC.

Competing interests

The authors report no competing interests.

For Review Only

Table 1 List of 76 nodes identified as working memory network with the broader cortical region to which the node belongs to

Region	Nodes
Anterior Cingulate and Medial Prefrontal Cortex (ACMPC)	8BM, d32
DorsoLateral Prefrontal Cortex (DLPC)	8C, 9-46d, a9-46v, p9-46v, i6-8, s6-8, 46
Inferior Frontal Cortex (IFC)	IFJp, 44, p47r
Inferior Parietal Cortex (IPC)	PF, PFm, IP2, IP1, PGs
Insular and Frontal Opercular Cortex (IFOC)	FOP4, FOP5, AVI
Orbital and Polar Frontal Cortex (OPFC)	p10p, a10p, 11l
Posterior Cingulate Cortex (PCC)	POS2, 7m
Premotor Cortex (PC)	6a, FEF, 55b, 6r
Superior Parietal Cortex (SPC)	AIP, LIPd, 7PL, 7Am, 7Pm, MIP
Caudate	Caudate
Putamen	Putamen
Thalamus	Thalamus

For Review Only

Table 2 Demographic and clinical characteristics of study participants

Characteristic	ALL survivors	Healthy controls
N	70	70
Age at imaging, mean (SD), range (years)	11.84 (4.13),<br>6.2–20.3	12.93 (3.37),<br>6.4–20.3
Sex (M/F)	40 / 30	40 / 30
Age at diagnosis, mean (SD), range (years)	9.3 (4.1),<br>3.7–17.7	NA
Risk arm, n (%)	Low: 34 (48.6%),<br>Standard/High: 36 (51.4%)	NA

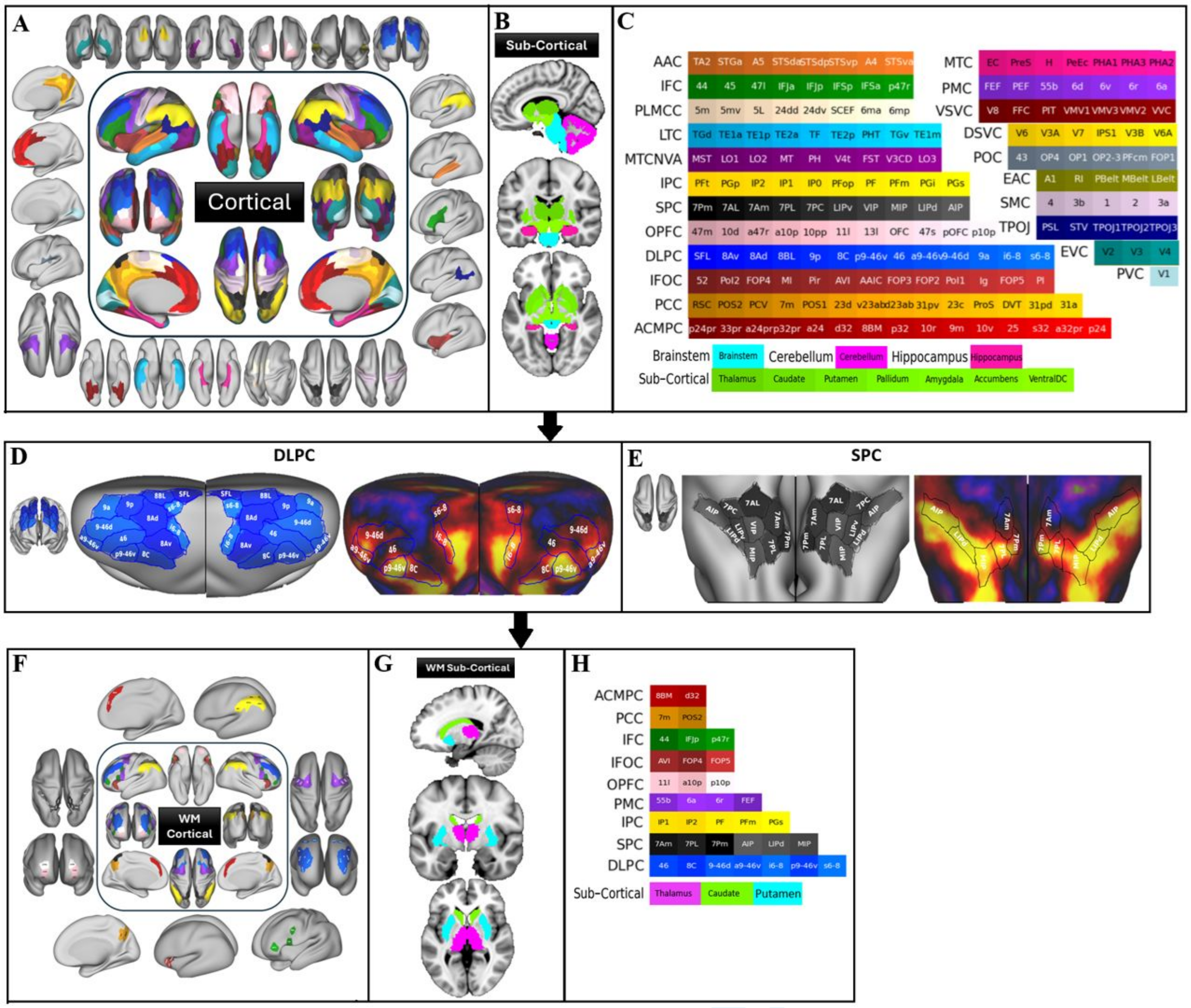

Figure 1 Framework for defining the working memory (WM) network based on the HCP-MMP1 atlas. A-C illustrate initial selection of atlas and corresponding parcellation: A. Visualization of the 180 bilateral cortical regions delineated by the HCP-MMP1 atlas; B. Illustration of eight selected subcortical regions, along with the cerebellum and brainstem; C. Detailed legend indicating each cortical and subcortical region with color coded labels corresponding to visualizations in A and B. D-E illustrate identification of predominantly activated regions using task-fMRI contrast maps (2-back vs. 0-back), shown for 2 representative regions, DLPC and SPC. D. Activated cortical regions in the DLPC; E. Activated regions within the SPC. Activation maps are overlaid onto the cortical surface, highlighting regions involved in WM tasks. The cortical folding maps and activation maps were adapted from the datasets available in BALSA at https://balsa.wustl.edu/ [25,59,60] with customized labels. F-H illustrate final selected WM network: F. Selected cortical regions forming the cortical component of the WM network; G. Subcortical regions included in the final WM network; H. Color coded legend indicating the final set of cortical and subcortical WM network regions displayed in F and G.

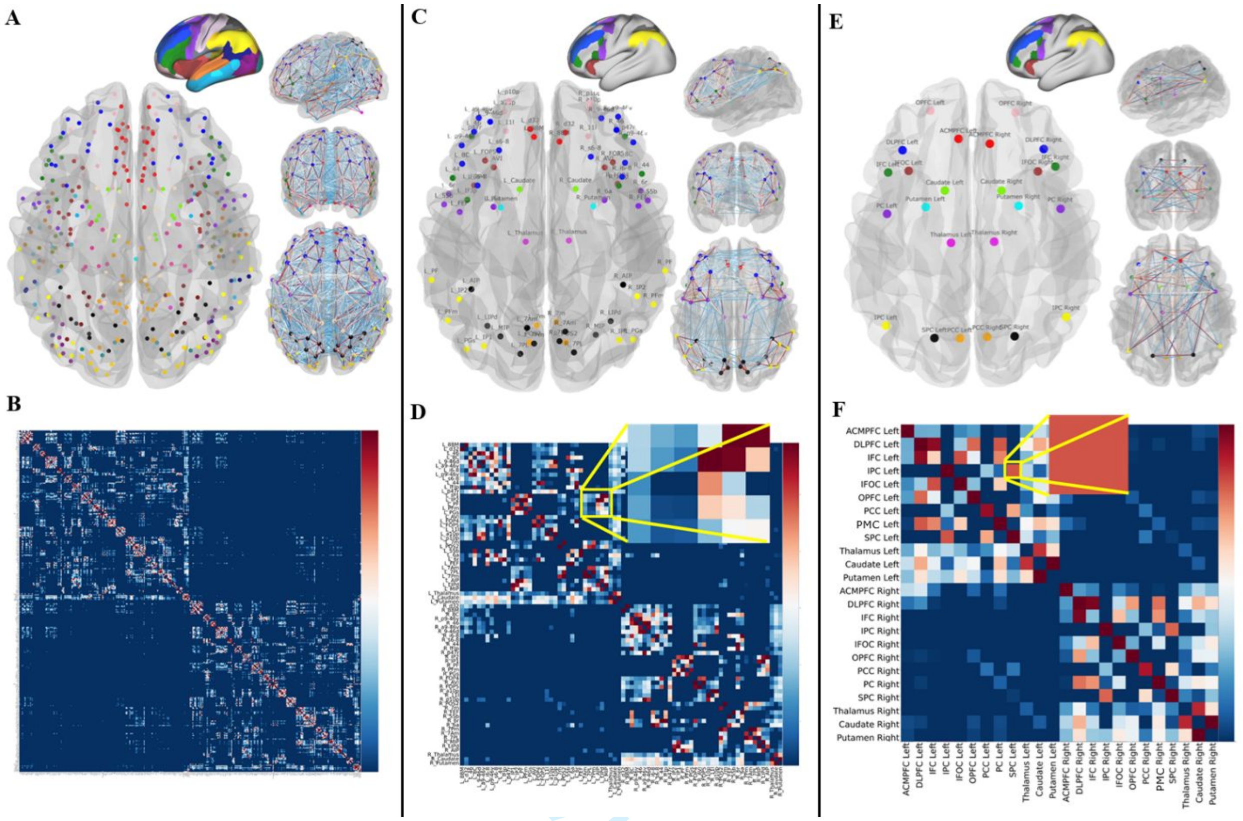

Figure 2 Multiscale construction of working memory (WM) structural connectivity matrices. This figure illustrates the hierarchical framework used to extract WM specific structural connectomes from the whole brain connectome and to generate multiscale representations. A–B Whole brain structural connectome based on the HCP-MMP1.0 parcellation (379 regions). A. Nodes are visualized anatomically. The inflated cortical maps were adapted from the datasets available in BALSA at https://balsa.wustl.edu/ [25,59,60] with customized labels. B. The corresponding 379×379 group averaged connectivity matrix is shown. C–D A 76-node WM subnetwork was extracted from the whole brain connectome. C. These functionally defined WM related nodes and their connectivity profiles are visualized. D. The resulting 76×76 submatrix is shown. The inset highlights all matrix entries representing pairwise connections between WM parcels assigned to left inferior parietal cortex (IPC) and left superior parietal cortex (SPC). (E–F) For coarse-scale analysis, the 76 WM nodes were grouped into 24 broader regions, consisting of 9 bilateral cortical and 3 bilateral subcortical divisions. F. The 24×24 matrix is shown. The inset shows the final aggregated edge weight between IPC left and SPC left, corresponding to the sum of the matrix elements highlighted in D.

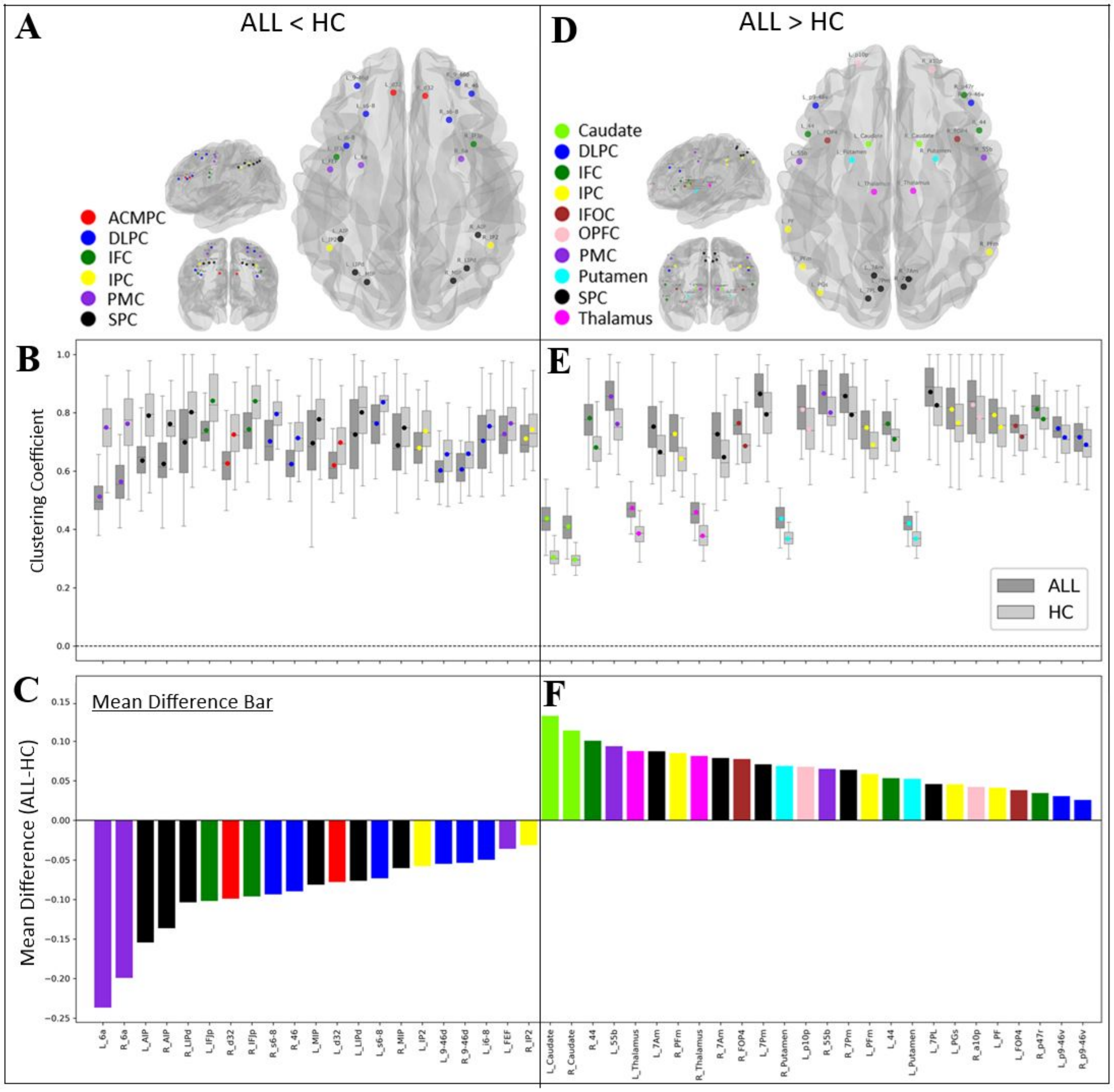

Figure 3 Group differences in clustering coefficient (CC) across the 76-node working memory (WM) structural network. A-C show significant results where CC was lower in ALL survivors compared to HC (ALL < HC), and D-F show significant results where CC was higher in ALL survivors (ALL > HC). A and D show glass brain plots highlighting significant, with legend indicating color coded cortical and subcortical regions. B and E show boxplots illustrating groupwise distribution of CC values for each significant node for ALL and HC groups. C and F show bar plots displaying the mean difference (ALL − HC) in CC for each node, illustrating direction and magnitude of group effects. Xtick labels are common across box and bar plots. ACMPC – anterior cingulate and medial prefrontal cortex, DLPC – dorsolateral prefrontal cortex, IFC – inferior frontal cortex, IFOC – inferior frontal occipital cortex, IPC – inferior parietal cortex, OPFC – orbito and polar frontal cortex, PMC – premotor cortex, SPC – superior parietal cortex.

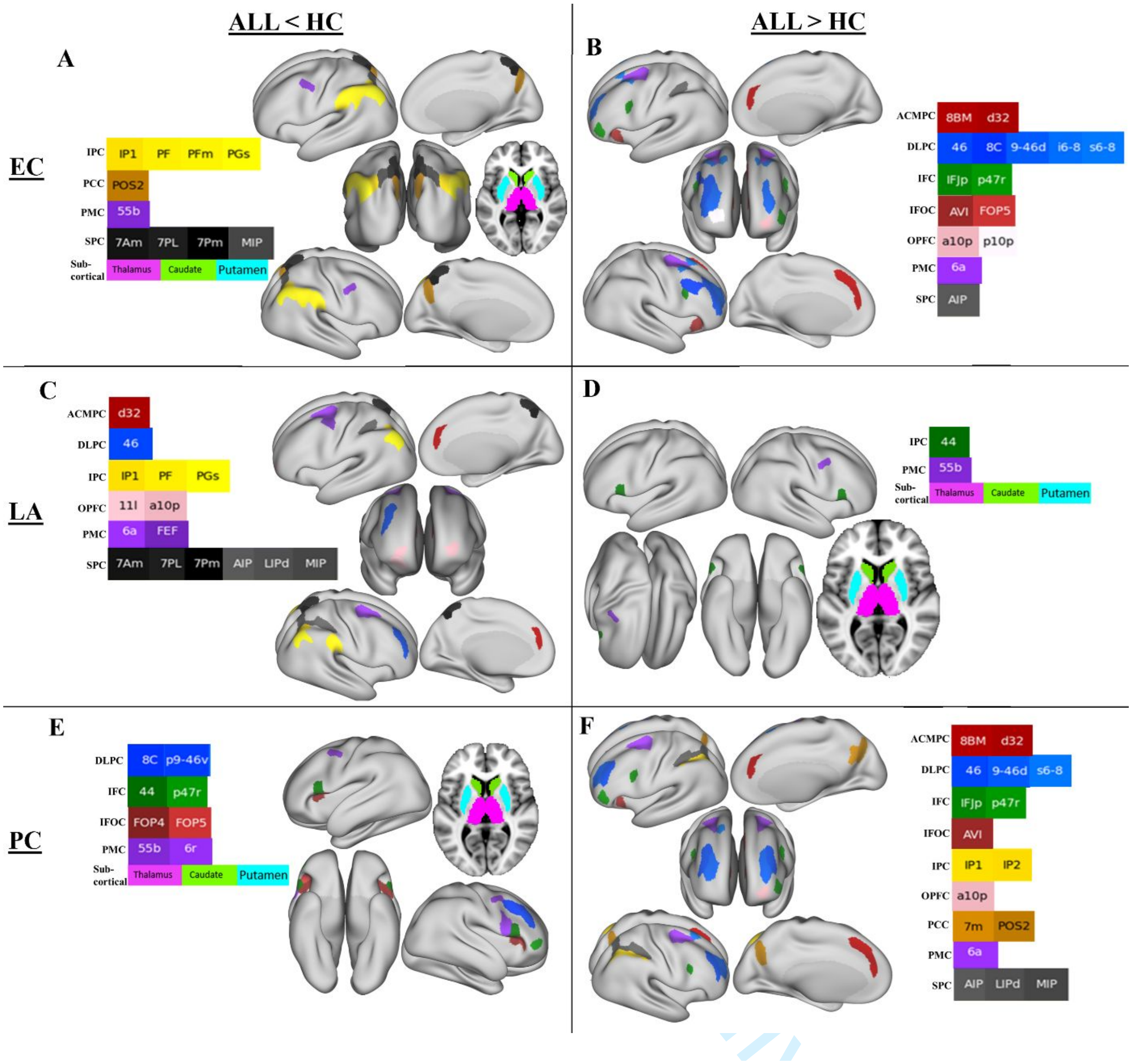

Figure 4 Consolidated group differences in Eigenvector centrality (EC), local assortativity (LA), and participation coefficient (PC) at the 76-node scale. Panels A, C, and E show regions with significantly reduced values in ALL survivors compared to controls (ALL < HC), while Panels B, D, and F show regions with significantly increased values in ALL survivors (ALL > HC). The inflated cortical maps were adapted from the datasets available in BALSA at https://balsa.wustl.edu/ [25,59,60] with customized labels. Full metric-specific glass brain plots (including boxplots and mean difference bars) are provided in Supplementary Fig. 1–3. ACMPC – anterior cingulate and medial prefrontal cortex, DLPC – dorsolateral prefrontal cortex, IFC – inferior frontal cortex, IFOC - insular and frontal opercular cortex, IPC - inferior parietal cortex, OPFC - orbital and polar frontal cortex, PCC - posterior cingulate cortex, PMC - premotor cortex, SPC - superior parietal cortex.

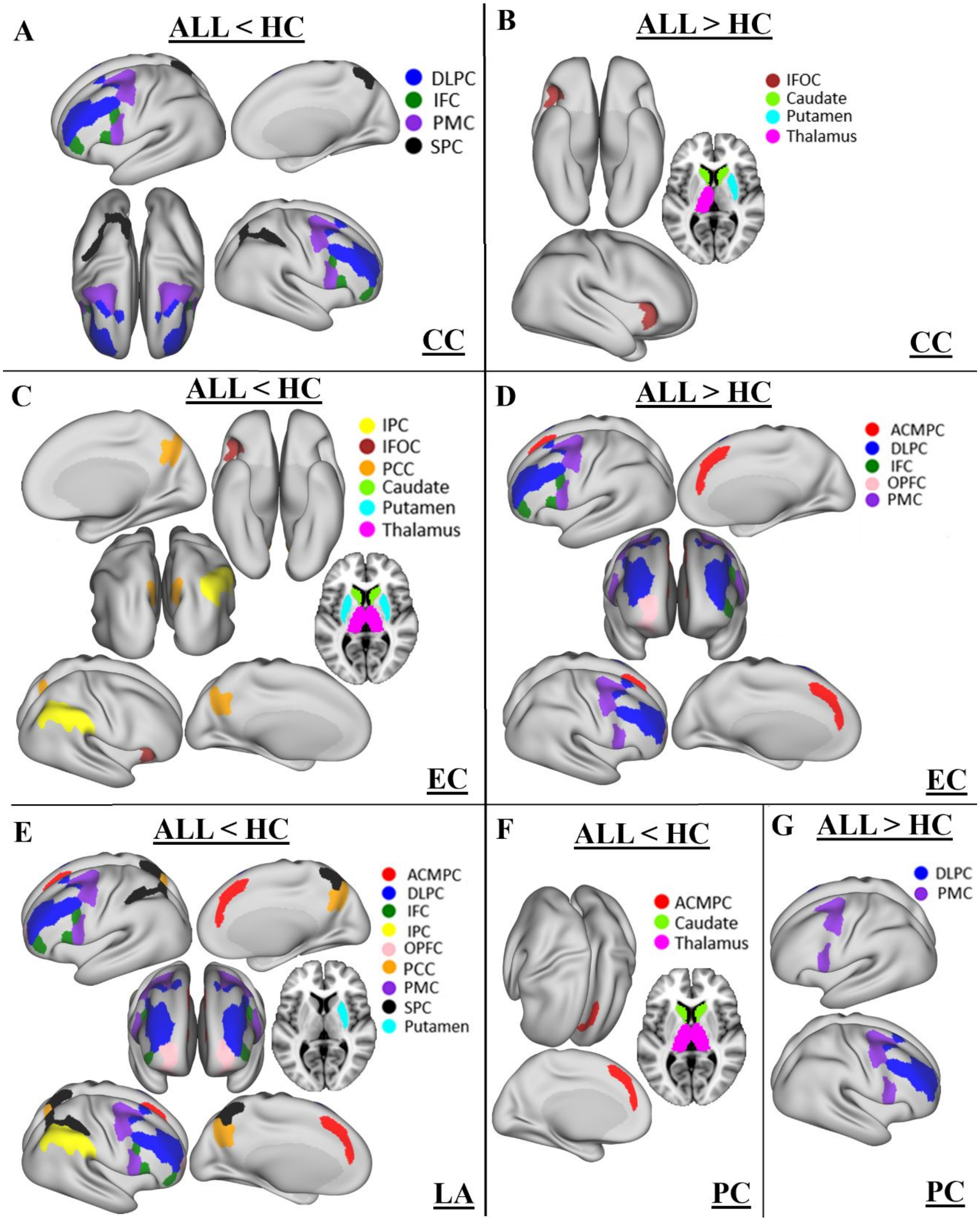

Figure 5 Consolidated group differences in clustering coefficient (CC), Eigenvector centrality (EC), local assortativity (LA), and participation coefficient (PC) at the 24-node scale. Panels A, C, E and F show regions with significantly reduced values in ALL survivors compared to controls (ALL < HC), while Panels B, D, and G show regions with significantly increased values in ALL survivors (ALL > HC). The inflated cortical maps were adapted from the datasets available in BALSA at https://balsa.wustl.edu/ [25,59,60] with customized labels. Full metric-specific plots (including boxplots and mean difference bars) are provided in Supplementary Fig. 4–7. ACMPC – anterior cingulate and medial prefrontal cortex, DLPC – dorsolateral prefrontal cortex, IFC – inferior frontal cortex, IFOC - insular and frontal opercular cortex, IPC - inferior parietal cortex, OPFC - orbital and polar frontal cortex, PCC - posterior cingulate cortex, PMC - premotor cortex, SPC - superior parietal cortex.

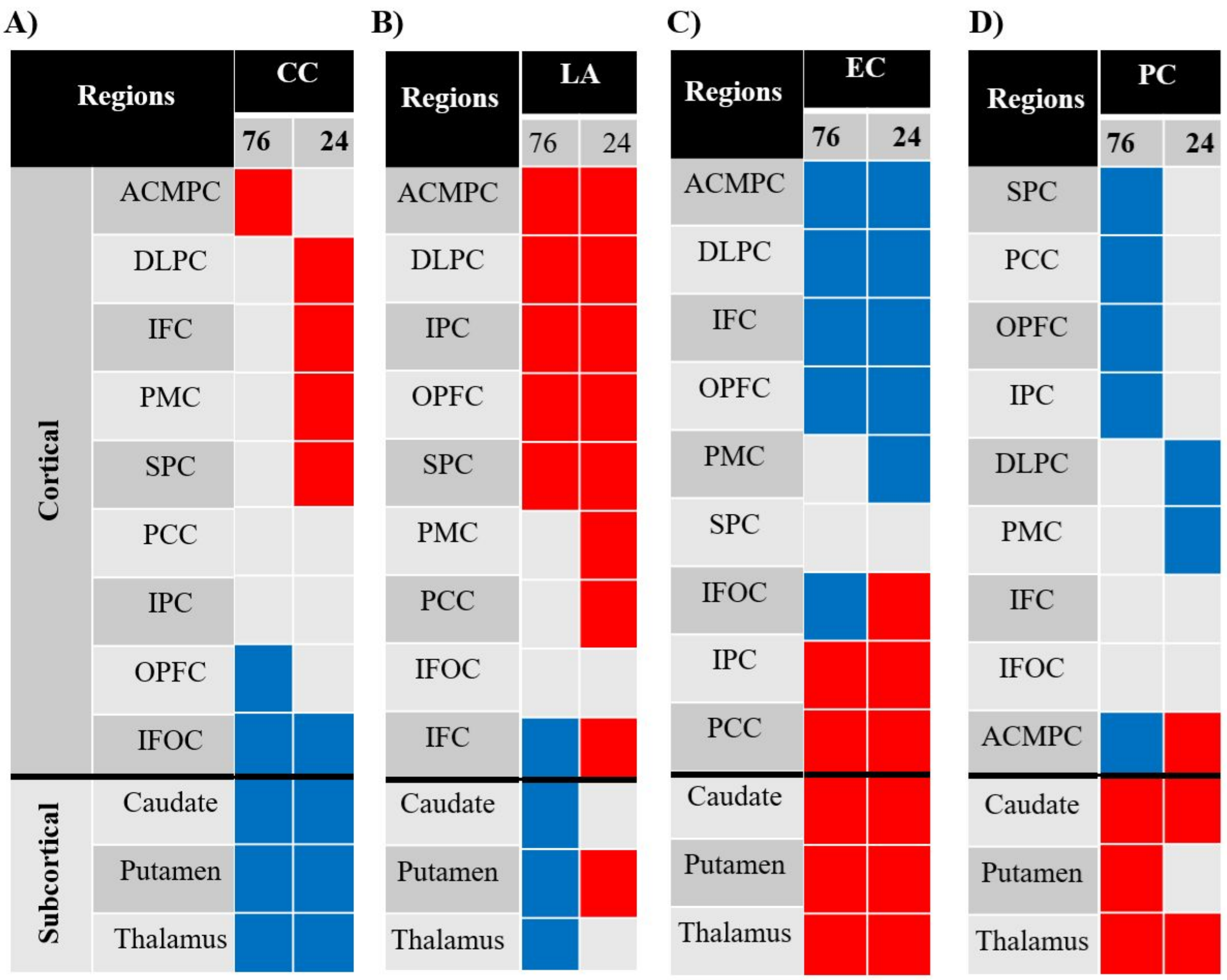

Figure 6 Inverse segregation - integration patterns in working memory (WM) network graph metrics between ALL survivors and healthy controls (HC). Color-coded matrices summarize group differences across four local graph metrics at two spatial scales (76- and 24-node WM networks) for cortical and subcortical regions. Each panel corresponds to a network metric: (A) Clustering Coefficient (CC), (B) Local Assortativity (LA), (C) Eigenvector Centrality (EC), and (D) Participation Coefficient (PC). Regions shown in red indicate significantly reduced values in ALL survivors compared to HC, while blue indicates increased values in survivors. Gray represents regions with heterogeneous fine-scale effects or no fine-scale or coarse-scale effect. Inverse pattern illustrated between metrics of segregation (CC, LA) and integration (EC, PC), with subcortical regions showing increased segregation but reduced integration. In contrast, cortical regions show the opposite pattern: reduced segregation and increased integration. ACMPC – anterior cingulate and medial prefrontal cortex, DLPC – dorsolateral prefrontal cortex, IFC – inferior frontal cortex, IPC – inferior parietal cortex, IFOC – inferior fronto occipital cortex, OPFC – orbitofrontal cortex, PCC – posterior cingulate cortex, PMC – premotor cortex, SPC – superior parietal cortex.

References

1. Baddeley A. Working memory: Theories, models, and controversies. *Annu Rev Psychol*. 2012;63. doi:10.1146/annurev-psych-120710-100422

2. Morrison AB, Chein JM. Does working memory training work? the promise and challenges of enhancing cognition by training working memory. *Psychon Bull Rev*. 2011;18(1). doi:10.3758/s13423-010-0034-0

3. Gathercole SE, Alloway TP. *Working Memory and Learning: A Practical Guide for Teachers*.; 2008.

4. van der Plas E, Modi AJ, Li CK, Krull KR, Cheung YT. Cognitive Impairment in Survivors of Pediatric Acute Lymphoblastic Leukemia Treated With Chemotherapy Only. *Journal of Clinical Oncology*. 2021;39(16). doi:10.1200/JCO.20.02322

5. D'Esposito M, Postle BR. The cognitive neuroscience of working memory. *Annu Rev Psychol*. 2015;66. doi:10.1146/annurev-psych-010814-015031

6. Krull KR, Hardy KK, Kahalley LS, Schuitema I, Kesler SR. Neurocognitive Outcomes and Interventions in Long-Term Survivors of Childhood Cancer. *J Clin Oncol*. 2018;36:2181-2189. doi:10.1200/JCO.2017

7. Robinson KE, Livesay KL, Campbell LK, et al. Working memory in survivors of childhood acute lymphocytic leukemia: Functional neuroimaging analyses. *Pediatr Blood Cancer*. 2010;54(4). doi:10.1002/pbc.22362

8. King TZ, Na S, Mao H. Neural Underpinnings of Working Memory in Adult Survivors of Childhood Brain Tumors. *Journal of the International Neuropsychological Society*. 2015;21(7). doi:10.1017/S135561771500051X

9. Siegwart V, Steiner L, Pastore-Wapp M, et al. The Working Memory Network and Its Association with Working Memory Performance in Survivors of non-CNS Childhood Cancer. *Dev Neuropsychol*. 2021;46(3):249-264. doi:10.1080/87565641.2021.1922410

10. Owen AM, McMillan KM, Laird AR, Bullmore E. N-back working memory paradigm: A meta-analysis of normative functional neuroimaging studies. In: *Human Brain Mapping*. Vol 25. 2005. doi:10.1002/hbm.20131

11. Wager TD, Smith EE. Neuroimaging studies of working memory: A meta-analysis. *Cogn Affect Behav Neurosci*. 2003;3(4):255-274. doi:10.3758/CABN.3.4.255

12. Fox MD, Raichle ME. Spontaneous fluctuations in brain activity observed with functional magnetic resonance imaging. *Nat Rev Neurosci*. 2007;8(9). doi:10.1038/nrn2201

13. Dosenbach NUF, Fair DA, Miezin FM, et al. Distinct brain networks for adaptive and stable task control in humans. *Proc Natl Acad Sci U S A*. 2007;104(26). doi:10.1073/pnas.0704320104

14. Fornito A, Zalesky A, Bullmore ET. Network scaling effects in graph analytic studies of human resting-state fMRI data. *Front Syst Neurosci*. 2010;4. doi:10.3389/fnsys.2010.00022

15. Cheung YT, Sabin ND, Reddick WE, et al. Association Between Acute Leukoencephalopathy and Long-Term Neurobehavioral and Brain Imaging Outcomes in Survivors of Childhood Acute Lymphoblastic Leukemia Treated with Chemotherapy Only. *Blood*. 2015;126(23). doi:10.1182/blood.v126.23.3255.3255

16. Hagmann P, Cammoun L, Gigandet X, et al. Mapping the Structural Core of Human Cerebral Cortex. *PLoS Biol*. 2008;6(7):e159. doi:10.1371/journal.pbio.0060159

17. Colombo M. Olaf Sporns: Networks of the Brain. *Minds Mach (Dordr)*. 2013;23(2). doi:10.1007/s11023-012-9294-y

For Review Only

18. Sporns O, Bullmore E. Complex brain networks: graph theoretical analysis of structural and functional systems. *Nat Rev Neurosci*. 2009;10.

19. Kesler SR, Ogg R, Reddick WE, et al. Brain Network Connectivity and Executive Function in Long-Term Survivors of Childhood Acute Lymphoblastic Leukemia. *Brain Connect*. 2018;8(6):333-342. doi:10.1089/brain.2017.0574

20. Faskowitz J, Yan X, Zuo XN, Sporns O. Weighted Stochastic Block Models of the Human Connectome across the Life Span. *Sci Rep*. 2018;8(1). doi:10.1038/s41598-018-31202-1

21. Crossley NA, Mechelli A, Vértes PE, et al. Cognitive relevance of the community structure of the human brain functional coactivation network. *Proc Natl Acad Sci U S A*. 2013;110(28). doi:10.1073/pnas.1220826110

22. Betzel RF, Avena-Koenigsberger A, Goñi J, et al. Generative models of the human connectome. *Neuroimage*. 2016;124. doi:10.1016/j.neuroimage.2015.09.041

23. Zalesky A, Fornito A, Harding IH, et al. Whole-brain anatomical networks: Does the choice of nodes matter? *Neuroimage*. 2010;50(3). doi:10.1016/j.neuroimage.2009.12.027

24. Schaefer A, Kong R, Gordon EM, et al. Local-Global Parcellation of the Human Cerebral Cortex from Intrinsic Functional Connectivity MRI. *Cerebral Cortex*. 2018;28(9). doi:10.1093/cercor/bhx179

25. Glasser MF, Coalson TS, Robinson EC, et al. A multi-modal parcellation of human cerebral cortex. *Nature*. 2016;536(7615):171-178. doi:10.1038/nature18933

26. Vasung L, Huang H, Jovanov-Milošević N, Pletikos M, Mori S, Kostović I. Development of axonal pathways in the human fetal fronto-limbic brain: Histochemical characterization and diffusion tensor imaging. *J Anat*. 2010;217(4). doi:10.1111/j.1469-7580.2010.01260.x

27. Cammoun L, Gigandet X, Meskaldji D, et al. Mapping the human connectome at multiple scales with diffusion spectrum MRI. *J Neurosci Methods*. 2012;203(2). doi:10.1016/j.jneumeth.2011.09.031

28. Jeha S, Pei D, Choi J, et al. Improved CNS Control of Childhood Acute Lymphoblastic Leukemia Without Cranial Irradiation: St Jude Total Therapy Study 16. *J Clin Oncol*. 2019;37:3377-3391. doi:10.1200/JCO.19

29. Jacola LM, Conklin HM, Krull KR, et al. The Impact of Intensified CNS-Directed Therapy on Neurocognitive Outcomes in Survivors of Childhood Acute Lymphoblastic Leukemia Treated Without Cranial Irradiation. *J Clin Oncol*. Published online 2022:0. doi:10.1200/JCO.22

30. Somerville LH, Bookheimer SY, Buckner RL, et al. The Lifespan Human Connectome Project in Development: A large-scale study of brain connectivity development in 5–21 year olds. *Neuroimage*. 2018;183:456-468. doi:10.1016/j.neuroimage.2018.08.050

31. Harms MP, Somerville LH, Ances BM, et al. Extending the Human Connectome Project across ages: Imaging protocols for the Lifespan Development and Aging projects. *Neuroimage*. 2018;183. doi:10.1016/j.neuroimage.2018.09.060

32. Smith SM, Jenkinson M, Woolrich MW, et al. Advances in functional and structural MR image analysis and implementation as FSL. *Neuroimage*. 2004;23(SUPPL. 1):S208-219. doi:10.1016/j.neuroimage.2004.07.051

33. Jeurissen B, Tournier JD, Dhollander T, Connelly A, Sijbers J. Multi-tissue constrained spherical deconvolution for improved analysis of multi-shell diffusion MRI data. *Neuroimage*. 2014;103. doi:10.1016/j.neuroimage.2014.07.061

For Review Only

34. Tournier JD, Smith R, Raffelt D, et al. MRtrix3: A fast, flexible and open software framework for medical image processing and visualisation. *Neuroimage*. 2019;202(August):116137. doi:10.1016/j.neuroimage.2019.116137

35. Tournier JD, , F. Calamante and a. C. Improved probabilistic streamlines tractography by 2 nd order integration over fibre orientation distributions. In: *Ismrm*. Vol 88. 2010:2010.

36. Smith RE, Tournier JD, Calamante F, Connelly A. SIFT2: Enabling dense quantitative assessment of brain white matter connectivity using streamlines tractography. *Neuroimage*. 2015;119. doi:10.1016/j.neuroimage.2015.06.092

37. Rubinov M, Sporns O. Complex network measures of brain connectivity: Uses and interpretations. *Neuroimage*. 2010;52(3):1059-1069. doi:10.1016/j.neuroimage.2009.10.003

38. Hagberg AA, Schult DA, Swart PJ. Exploring Network Structure, Dynamics, and Function using NetworkX. In: *Proceedings of the 7th Python in Science Conference*. 2008. doi:10.25080/tcwv9851

39. Benjamini Y, Hochberg Y. Controlling the False Discovery Rate: A Practical and Powerful Approach to Multiple Testing. *Journal of the Royal Statistical Society: Series B (Methodological)*. 1995;57(1):289-300. doi:10.1111/j.2517-6161.1995.tb02031.x

40. Choi EY, Thomas Yeo BT, Buckner RL. The organization of the human striatum estimated by intrinsic functional connectivity. *J Neurophysiol*. 2012;108(8). doi:10.1152/jn.00270.2012

41. Menon V, Uddin LQ. Saliency, switching, attention and control: a network model of insula function. *Brain Struct Funct*. 2010;214(5-6). doi:10.1007/s00429-010-0262-0

42. Krull KR, Brinkman TM, Li C, et al. Neurocognitive outcomes decades after treatment for childhood acute lymphoblastic leukemia: A report from the St jude lifetime cohort study. *Journal of Clinical Oncology*. 2013;31(35). doi:10.1200/JCO.2012.48.2315

43. McNab F, Klingberg T. Prefrontal cortex and basal ganglia control access to working memory. *Nat Neurosci*. 2008;11(1). doi:10.1038/nn2024

44. Zuo XN, Ehmke R, Mennes M, et al. Network centrality in the human functional connectome. *Cerebral Cortex*. 2012;22(8). doi:10.1093/cercor/bhr269

45. Cole MW, Yarkoni T, Repovš G, Anticevic A, Braver TS. Global connectivity of prefrontal cortex predicts cognitive control and intelligence. *Journal of Neuroscience*. 2012;32(26). doi:10.1523/JNEUROSCI.0536-12.2012

46. Kesler SR, Gugel M, Huston-Warren E, Watson C. Atypical Structural Connectome Organization and Cognitive Impairment in Young Survivors of Acute Lymphoblastic Leukemia. *Brain Connect*. 2016;6(4):273-282. doi:10.1089/brain.2015.0409

47. Frank MJ, Loughry B, O'Reilly RC. Interactions between frontal cortex and basal ganglia in working memory: A computational model. *Cogn Affect Behav Neurosci*. 2001;1(2). doi:10.3758/CABN.1.2.137

48. Thedchanamoorthy G, Piraveenan M, Kasthuriratna D, Senanayake U. Node assortativity in complex networks: An alternative approach. In: *Procedia Computer Science*. Vol 29. 2014. doi:10.1016/j.procs.2014.05.229

49. Vincent JL, Kahn I, Snyder AZ, Raichle ME, Buckner RL. Evidence for a frontoparietal control system revealed by intrinsic functional connectivity. *J Neurophysiol*. 2008;100(6). doi:10.1152/jn.90355.2008

For Review Only

50. Guimerà R, Amaral LAN. Functional cartography of complex metabolic networks. *Nature*. 2005;433(7028). doi:10.1038/nature03288

51. Hwang K, Bertolero MA, Liu WB, D'Esposito M. The human thalamus is an integrative hub for functional brain networks. *Journal of Neuroscience*. 2017;37(23). doi:10.1523/JNEUROSCI.0067-17.2017

52. Barbas H. General Cortical and Special Prefrontal Connections: Principles from Structure to Function. *Annu Rev Neurosci*. 2015;38. doi:10.1146/annurev-neuro-071714-033936

53. Niendam TA, Laird AR, Ray KL, Dean YM, Glahn DC, Carter CS. Meta-analytic evidence for a superordinate cognitive control network subserving diverse executive functions. *Cogn Affect Behav Neurosci*. 2012;12(2). doi:10.3758/s13415-011-0083-5

54. Wang L, Zhu C, He Y, et al. Altered small-world brain functional networks in children with attention-deficit/hyperactivity disorder. *Hum Brain Mapp*. 2009;30(2). doi:10.1002/hbm.20530

55. van Wijk BCM, Stam CJ, Daffertshofer A. Comparing brain networks of different size and connectivity density using graph theory. *PLoS One*. 2010;5(10). doi:10.1371/journal.pone.0013701

56. Power JD, Cohen AL, Nelson SM, et al. Functional Network Organization of the Human Brain. *Neuron*. 2011;72(4). doi:10.1016/j.neuron.2011.09.006

57. Shine JM, Bissett PG, Bell PT, et al. The Dynamics of Functional Brain Networks: Integrated Network States during Cognitive Task Performance. *Neuron*. 2016;92(2). doi:10.1016/j.neuron.2016.09.018

58. Shine JM, Poldrack RA. Principles of dynamic network reconfiguration across diverse brain states. *Neuroimage*. 2018;180. doi:10.1016/j.neuroimage.2017.08.010

59. Marcus DS, Harwell J, Olsen T, et al. Informatics and data mining tools and strategies for the human connectome project. *Front Neuroinform*. 2011;5. doi:10.3389/fninf.2011.00004

60. Van Essen DC, Smith J, Glasser MF, et al. The Brain Analysis Library of Spatial maps and Atlases (BALSA) database. *Neuroimage*. 2017;144. doi:10.1016/j.neuroimage.2016.04.002

For Review Only

Table 1 List of 76 nodes identified as working memory network with the broader cortical region to which the node belongs to

Region	Nodes
Anterior Cingulate and Medial Prefrontal Cortex (ACMPC)	8BM, d32
DorsoLateral Prefrontal Cortex (DLPC)	8C, 9-46d, a9-46v, p9-46v, i6-8, s6-8, 46
Inferior Frontal Cortex (IFC)	IFJp, 44, p47r
Inferior Parietal Cortex (IPC)	PF, PFm, IP2, IP1, PGs
Insular and Frontal Opercular Cortex (IFOC)	FOP4, FOP5, AVI
Orbital and Polar Frontal Cortex (OPFC)	p10p, a10p, 11l
Posterior Cingulate Cortex (PCC)	POS2, 7m
Premotor Cortex (PC)	6a, FEF, 55b, 6r
Superior Parietal Cortex (SPC)	AIP, LIPd, 7PL, 7Am, 7Pm, MIP
Caudate	Caudate
Putamen	Putamen
Thalamus	Thalamus

For Review Only

Table 2 Demographic and clinical characteristics of study participants

Characteristic	ALL survivors	Healthy controls
N	70	70
Age at imaging, mean (SD), range (years)	11.84 (4.13), 6.2–20.3	12.93 (3.37), 6.4–20.3
Sex (M/F)	40 / 30	40 / 30
Age at diagnosis, mean (SD), range (years)	9.3 (4.1), 3.7–17.7	NA
Risk arm, n (%)	Low: 34 (48.6%), Standard/High: 36 (51.4%)	NA

For Review Only

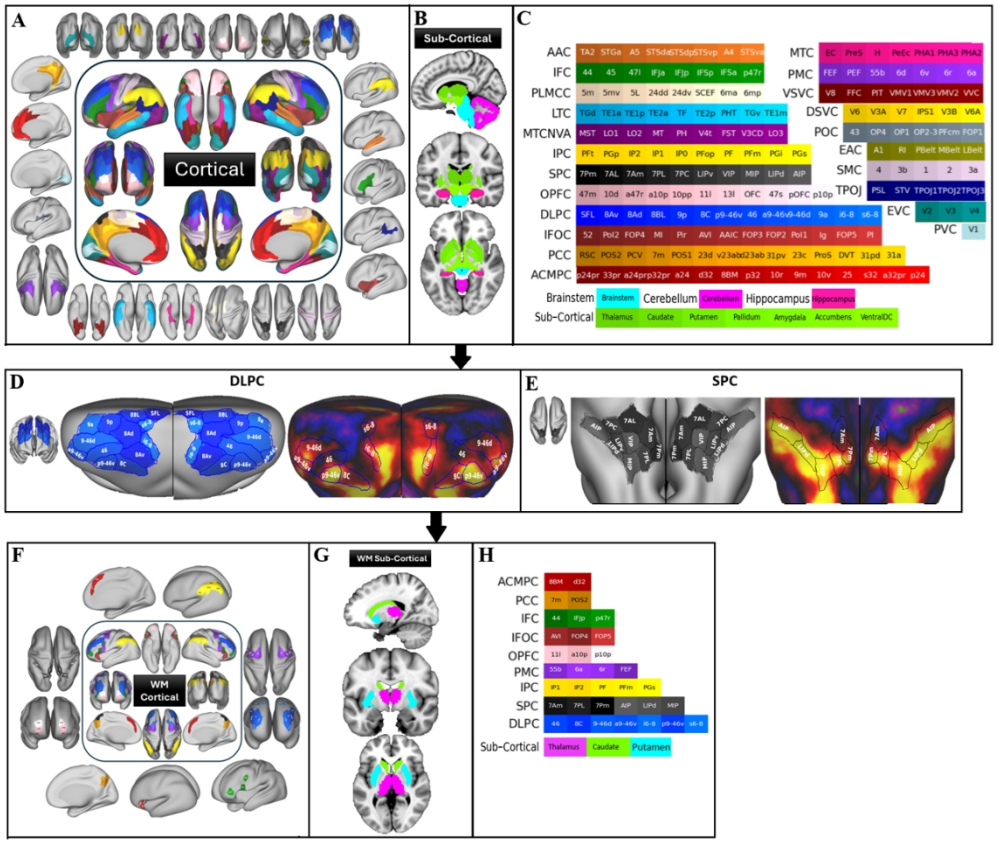

Figure 1 Framework for defining the working memory (WM) network based on the HCP-MMP1 atlas. A-C illustrate initial selection of atlas and corresponding parcellation: A. Visualization of the 180 bilateral cortical regions delineated by the HCP-MMP1 atlas; B. Illustration of eight selected subcortical regions, along with the cerebellum and brainstem; C. Detailed legend indicating each cortical and subcortical region with color coded labels corresponding to visualizations in A and B. D-E illustrate identification of predominantly activated regions using task-fMRI contrast maps (2-back vs. 0-back), shown for 2 representative regions, DLPC and SPC. D. Activated cortical regions in the DLPC; E. Activated regions within the SPC. Activation maps are overlaid onto the cortical surface, highlighting regions involved in WM tasks. The cortical folding maps and activation maps were adapted from the datasets available in BALSA at https://balsa.wustl.edu/ 25,59,60 with customized labels. F-H illustrate final selected WM network: F. Selected cortical regions forming the cortical component of the WM network; G. Subcortical regions included in the final WM network; H. Color coded legend indicating the final set of cortical and subcortical WM network regions displayed in F and G.

858x722mm (38 x 38 DPI)

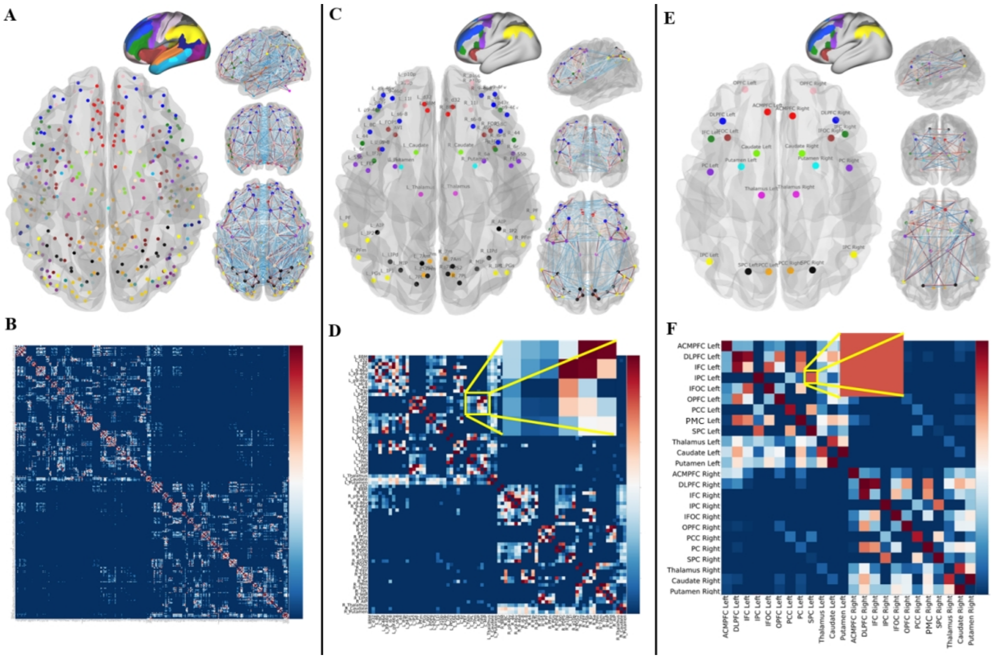

Figure 2 Multiscale construction of working memory (WM) structural connectivity matrices. This figure illustrates the hierarchical framework used to extract WM specific structural connectomes from the whole brain connectome and to generate multiscale representations. A–B Whole brain structural connectome based on the HCP-MMP1.0 parcellation (379 regions). A. Nodes are visualized anatomically. The inflated cortical maps were adapted from the datasets available in BALSA at https://balsa.wustl.edu/ 25,59,60 with customized labels. B. The corresponding 379×379 group averaged connectivity matrix is shown. C–D A 76-node WM subnetwork was extracted from the whole brain connectome. C. These functionally defined WM related nodes and their connectivity profiles are visualized. D. The resulting 76×76 submatrix is shown. The inset highlights all matrix entries representing pairwise connections between WM parcels assigned to left inferior parietal cortex (IPC) and left superior parietal cortex (SPC). (E–F) For coarse-scale analysis, the 76 WM nodes were grouped into 24 broader regions, consisting of 9 bilateral cortical and 3 bilateral subcortical divisions. F. The 24×24 matrix is shown. The inset shows the final aggregated edge weight between IPC left and SPC left, corresponding to the sum of the matrix elements highlighted in D.

1008x657mm (38 x 38 DPI)

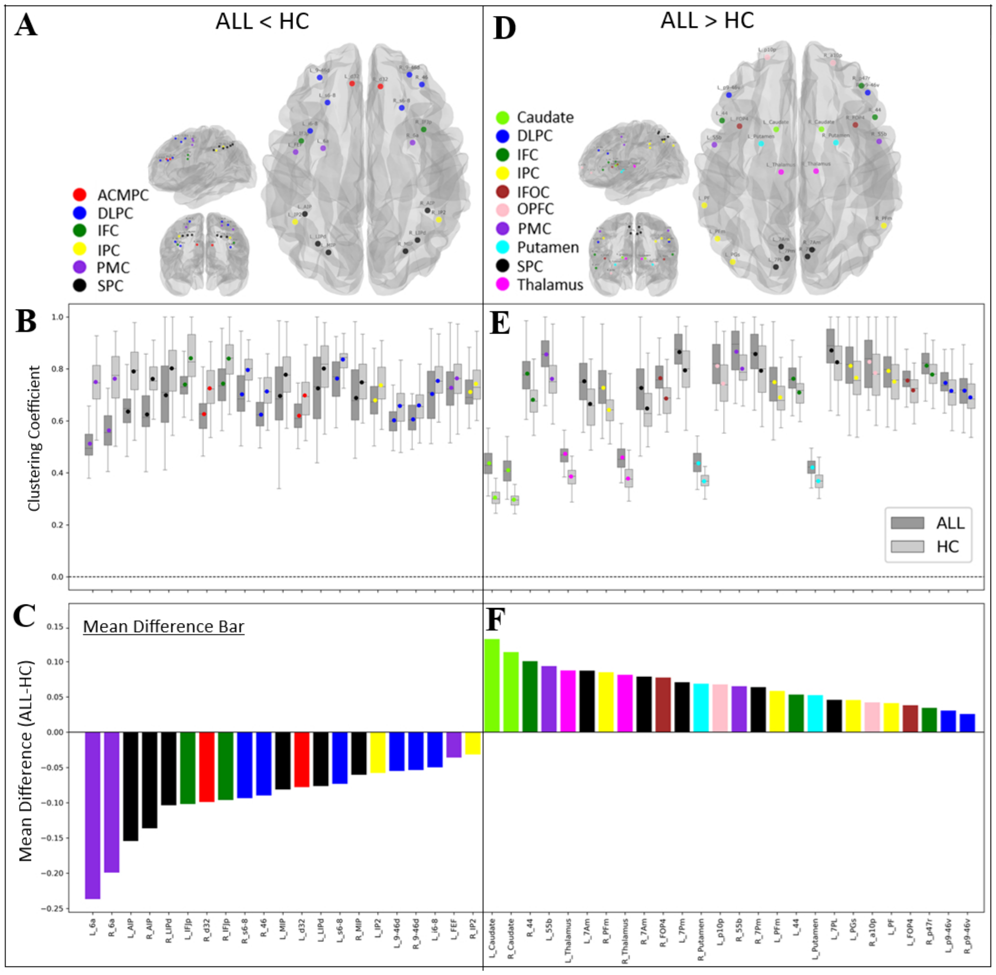

Figure 3 Group differences in clustering coefficient (CC) across the 76-node working memory (WM) structural network. A-C show significant results where CC was lower in ALL survivors compared to HC (ALL < HC), and D-F show significant results where CC was higher in ALL survivors (ALL > HC). A and D show glass brain plots highlighting significant, with legend indicating color coded cortical and subcortical regions. B and E show boxplots illustrating groupwise distribution of CC values for each significant node for ALL and HC groups. C and F show bar plots displaying the mean difference (ALL − HC) in CC for each node, illustrating direction and magnitude of group effects. Xtick labels are common across box and bar plots. ACMPC – anterior cingulate and medial prefrontal cortex, DLPC – dorsolateral prefrontal cortex, IFC – inferior frontal cortex, IFOC – inferior frontal occipital cortex, IPC – inferior parietal cortex, OPFC – orbito and polar frontal cortex, PMC – premotor cortex, SPC – superior parietal cortex.

735x717mm (38 x 38 DPI)

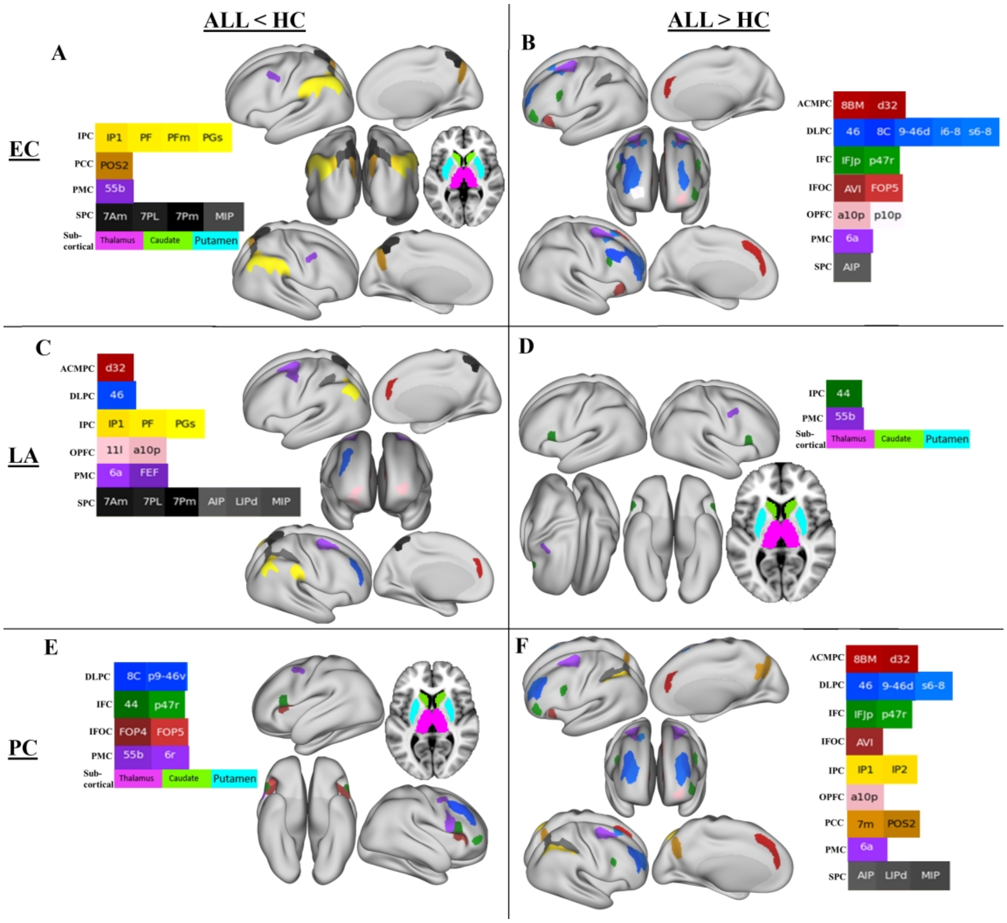

Figure 4 Consolidated group differences in Eigenvector centrality (EC), local assortativity (LA), and participation coefficient (PC) at the 76-node scale. Panels A, C, and E show regions with significantly reduced values in ALL survivors compared to controls (ALL < HC), while Panels B, D, and F show regions with significantly increased values in ALL survivors (ALL > HC). The inflated cortical maps were adapted from the datasets available in BALSA at https://balsa.wustl.edu/ 25,59,60 with customized labels. Full metric-specific glass brain plots (including boxplots and mean difference bars) are provided in Supplementary Fig. 1–3. ACMPC – anterior cingulate and medial prefrontal cortex, DLPC – dorsolateral prefrontal cortex, IFC – inferior frontal cortex, IFOC - insular and frontal opercular cortex, IPC - inferior parietal cortex, OPFC - orbital and polar frontal cortex, PCC - posterior cingulate cortex, PMC - premotor cortex, SPC - superior parietal cortex.

1915x1750mm (38 x 38 DPI)

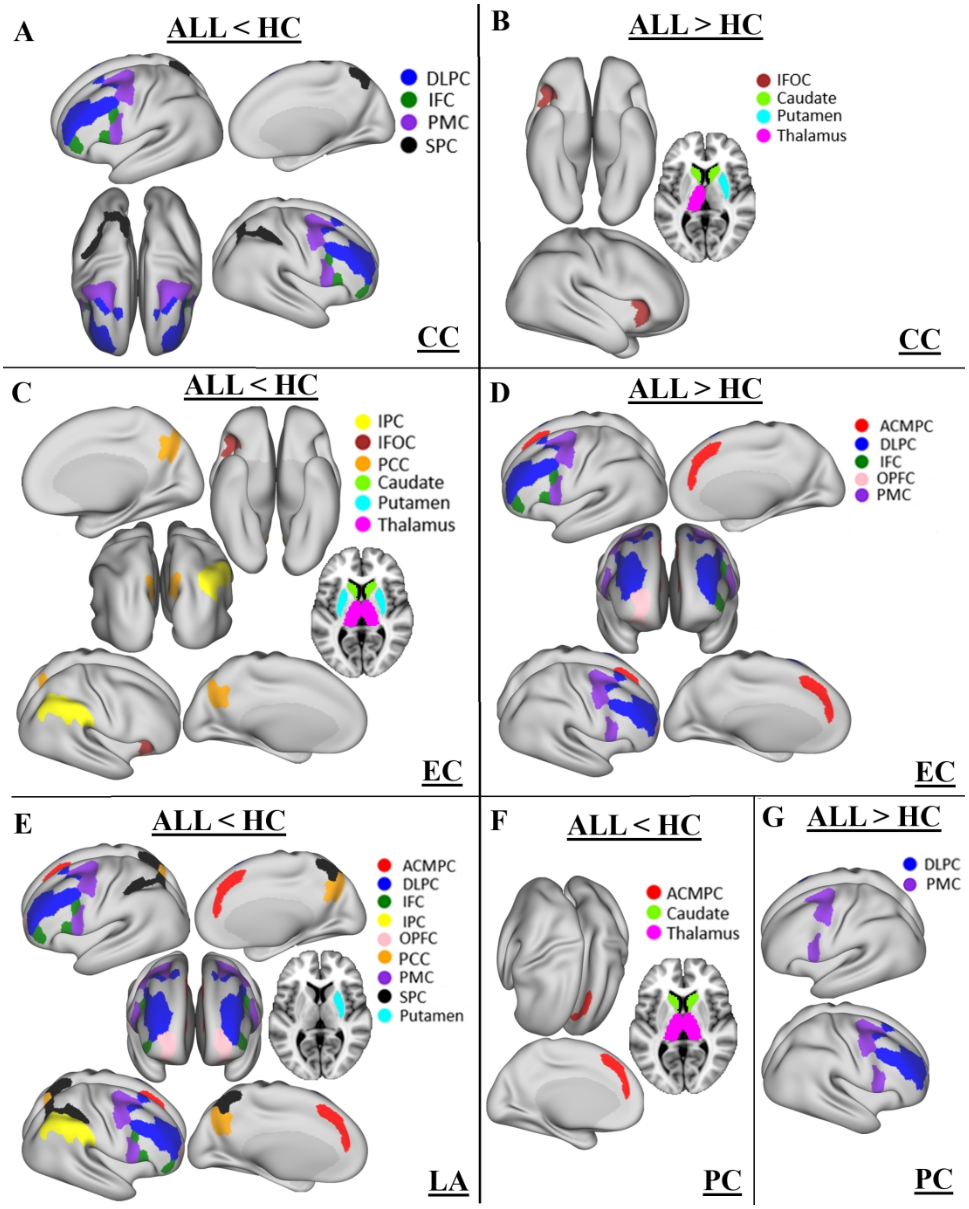

Figure 5 Consolidated group differences in clustering coefficient (CC), Eigenvector centrality (EC), local assortativity (LA), and participation coefficient (PC) at the 24-node scale. Panels A, C, E and F show regions with significantly reduced values in ALL survivors compared to controls (ALL < HC), while Panels B, D, and G show regions with significantly increased values in ALL survivors (ALL > HC). The inflated cortical maps were adapted from the datasets available in BALSA at https://balsa.wustl.edu/ 25,59,60 with customized labels. Full metric-specific plots (including boxplots and mean difference bars) are provided in Supplementary Fig. 4–7. ACMPC – anterior cingulate and medial prefrontal cortex, DLPC – dorsolateral prefrontal cortex, IFC – inferior frontal cortex, IFOC - insular and frontal opercular cortex, IPC - inferior parietal cortex, OPFC - orbital and polar frontal cortex, PCC - posterior cingulate cortex, PMC - premotor cortex, SPC - superior parietal cortex.

1399x1745mm (38 x 38 DPI)

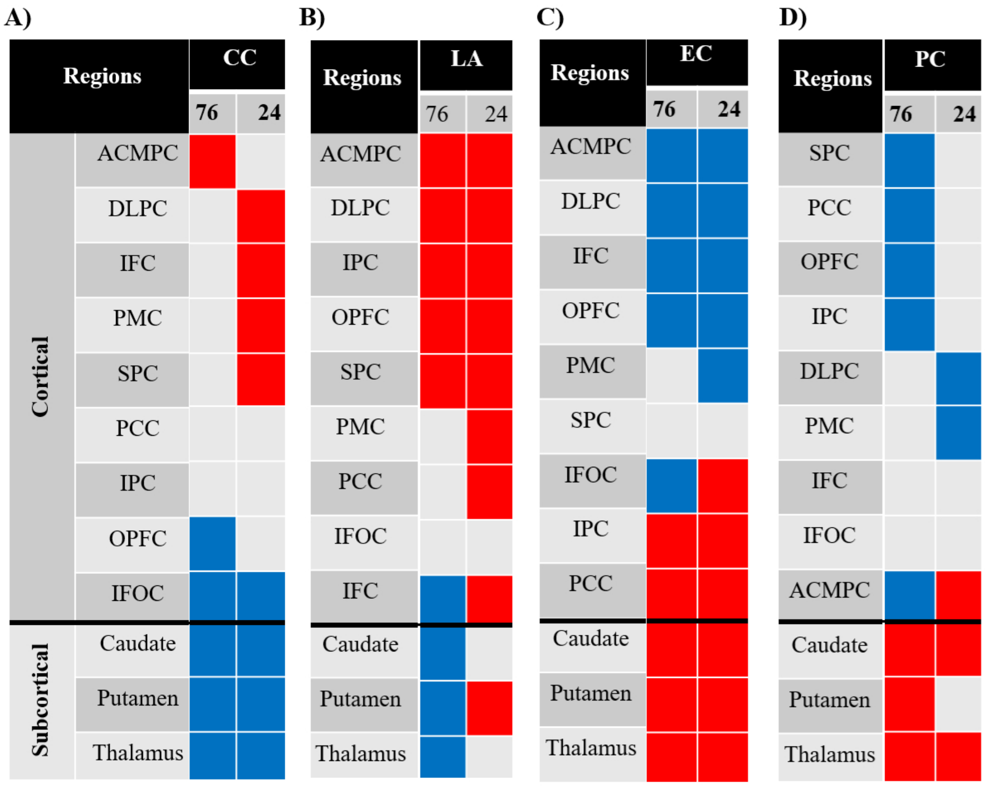

Figure 6 Inverse segregation - integration patterns in working memory (WM) network graph metrics between ALL survivors and healthy controls (HC). Color-coded matrices summarize group differences across four local graph metrics at two spatial scales (76- and 24-node WM networks) for cortical and subcortical regions. Each panel corresponds to a network metric: (A) Clustering Coefficient (CC), (B) Local Assortativity (LA), (C) Eigenvector Centrality (EC), and (D) Participation Coefficient (PC). Regions shown in red indicate significantly reduced values in ALL survivors compared to HC, while blue indicates increased values in survivors. Gray represents regions with heterogeneous fine-scale effects or no fine-scale or coarse-scale effect. Inverse pattern illustrated between metrics of segregation (CC, LA) and integration (EC, PC), with subcortical regions showing increased segregation but reduced integration. In contrast, cortical regions show the opposite pattern: reduced segregation and increased integration. ACMPC – anterior cingulate and medial prefrontal cortex, DLPC – dorsolateral prefrontal cortex, IFC – inferior frontal cortex, IPC – inferior parietal cortex, IFOC – inferior fronto occipital cortex, OPFC – orbitofrontal cortex, PCC – posterior cingulate cortex, PMC – premotor cortex, SPC – superior parietal cortex.

806x639mm (38 x 38 DPI)

Supplementary Materials

Multiscale Connectivity Framework for Working Memory Network in Pediatric Acute Lymphoblastic Leukemia Survivors

Supplementary Tables

Supplementary Table 1. Local network metrics used in the analysis. Definitions are based on Brain Connectivity Toolbox[1] and NetworkX,[2] with interpretations informed by[3–7]

Metric	Definition	Integration or Segregation	Impact of Higher Values in Human Brain Structural Connectivity	Impact of Lower Values in Human Brain Structural Connectivity
**Clustering Coefficient (CC)**	Fraction of a node's neighbors that are interconnected, indicating tightly knit local clusters.[1,6]	Segregation	Indicates stronger local specialization and redundant short-range connectivity, supporting modular processing in healthy brains.[8]	Reflects reduced local clustering and loss of specialization, often observed in disrupted or diseased structural networks.[9]
**Eigenvector Centrality (EC)**	Measure of a node's influence based on connections to other highly connected nodes.[3,4]	Integration	Node functions as a global hub, enhancing integrative communication across distributed regions and supporting complex cognition.[3,10]	Reduced hubness, with diminished contribution to global integration, associated with cognitive deficits in clinical populations.[11]
**Local Assortativity (LA)**	Extent to which a node links to neighbors with similar levels of connectivity.[1,5]	Segregation	Indicates preservation of modular segregation, with nodes linking to similarly connected neighbors, consistent with intact modular structure.[1]	Reflects heterogeneous local wiring and reduced modularity, potentially observed in network disorganization in disease.[12]
**Participation Coefficient (PC)**	Proportion of a node's connections distributed across different modules, reflecting its role in cross-module integration.[1,7]	Integration	Node bridges across modules, enabling cross-network integration and flexible communication.[7,13]	Node is confined to within-module connections, limiting integration and possibly reflecting structural disconnection.[14]

For Review Only

Supplementary Table 2. Statistical results for clustering coefficient (CC) at the 76-node scale. Only significant results are listed. Rows are ordered by estimate sign (positive first and then negative), followed by alphabetical order of anatomical region within each group. Columns are defined as follows: Node – fine-scale parcellation label, with prefixes L_ and R_ indicating left and right hemisphere nodes, respectively; Anatomical Region – broader cortical or subcortical grouping; Estimate – group mean difference (contrast: mean[HC] – mean[ALL]), where positive values indicate decreased CC in ALL survivors (ALL < HC) and negative values indicate increased CC in ALL survivors (ALL > HC); Raw P-value – uncorrected p-value; FDR P-value – false discovery rate adjusted P-value. All values are rounded to four decimal places.

Node	Anatomical Region	Estimate	Raw P-value	FDR P-value
L_d32	ACMPC Left	0.0778	0.0000	0.0000
R_d32	ACMPC Right	0.0993	0.0000	0.0000
L_s6-8	DLPC Left	0.0693	0.0000	0.0000

L_9-46d	DLPC Left	0.0514	0.0000	0.0001
L_i6-8	DLPC Left	0.0497	0.0039	0.0073
R_s6-8	DLPC Right	0.0928	0.0000	0.0000
R_46	DLPC Right	0.0872	0.0000	0.0000
R_9-46d	DLPC Right	0.0500	0.0000	0.0000
L_IFJp	IFC Left	0.1007	0.0000	0.0000
R_IFJp	IFC Right	0.0994	0.0000	0.0000
L_IP2	IPC Left	0.0600	0.0000	0.0001
R_IP2	IPC Right	0.0314	0.0191	0.0331
L_6a	PMC Left	0.2392	0.0000	0.0000
L_FEF	PMC Left	0.0391	0.0199	0.0336
R_6a	PMC Right	0.2006	0.0000	0.0000
L_AIP	SPC Left	0.1532	0.0000	0.0000
L_LIPd	SPC Left	0.0887	0.0000	0.0001
L_MIP	SPC Left	0.0805	0.0000	0.0001
R_AIP	SPC Right	0.1374	0.0000	0.0000
R_LIPd	SPC Right	0.1100	0.0000	0.0000
R_MIP	SPC Right	0.0567	0.0027	0.0052
L_p9-46v	DLPC Left	-0.0293	0.0168	0.0297
R_p9-46v	DLPC Right	-0.0277	0.0305	0.0493
L_44	IFC Left	-0.0558	0.0000	0.0000
R_p47r	IFC Right	-0.0381	0.0006	0.0013
R_44	IFC Right	-0.1006	0.0000	0.0000
L_FOP4	IFOC Left	-0.0393	0.0001	0.0003
R_FOP4	IFOC Right	-0.0807	0.0000	0.0000
L_PF	IPC Left	-0.0441	0.0047	0.0085
L_PGs	IPC Left	-0.0485	0.0029	0.0056
L_PFm	IPC Left	-0.0574	0.0001	0.0002
R_PFm	IPC Right	-0.0867	0.0000	0.0000
L_p10p	OPFC Left	-0.0698	0.0000	0.0000
R_a10p	OPFC Right	-0.0405	0.0246	0.0406
L_55b	PMC Left	-0.0931	0.0000	0.0000
R_55b	PMC Right	-0.0673	0.0000	0.0000
L_7Pm	SPC Left	-0.0755	0.0000	0.0000
L_7Am	SPC Left	-0.0918	0.0000	0.0000
L_7PL	SPC Left	-0.0520	0.0002	0.0005
R_7Am	SPC Right	-0.0830	0.0000	0.0000
R_7Pm	SPC Right	-0.0643	0.0005	0.0011
R_Caudate	Caudate Right	-0.1153	0.0000	0.0000
L_Caudate	Caudate Left	-0.1361	0.0000	0.0000
L_Putamen	Putamen Left	-0.0573	0.0000	0.0000

R_Putamen	Putamen Right	-0.0737	0.0000	0.0000
R_Thalamus	Thalamus Right	-0.0856	0.0000	0.0000
L_Thalamus	Thalamus Left	-0.0910	0.0000	0.0000

Supplementary Table 3. Statistical results for Eigenvector centrality (EC) at the 76-node scale. Only significant results are listed. Rows are ordered by estimate sign (positive first and then negative), followed by alphabetical order of anatomical region within each group. Columns are defined as in Supplementary Table 2. The Estimate column reflects the group mean difference (mean[HC] – mean[ALL]), with positive values indicating decreased EC in ALL survivors and negative values indicating increased EC in ALL survivors. All values are rounded to four decimal places.

Node	Anatomical Region	Estimate	Raw P-value	FDR P-value
L_d32	ACMPC Left	0.0778	0.0000	0.0000
R_d32	ACMPC Right	0.0993	0.0000	0.0000
L_s6-8	DLPC Left	0.0693	0.0000	0.0000
L_9-46d	DLPC Left	0.0514	0.0000	0.0001
L_i6-8	DLPC Left	0.0497	0.0039	0.0073
R_s6-8	DLPC Right	0.0928	0.0000	0.0000
R_46	DLPC Right	0.0872	0.0000	0.0000
R_9-46d	DLPC Right	0.0500	0.0000	0.0000
L_IFJp	IFC Left	0.1007	0.0000	0.0000
R_IFJp	IFC Right	0.0994	0.0000	0.0000
L_IP2	IPC Left	0.0600	0.0000	0.0001
R_IP2	IPC Right	0.0314	0.0191	0.0331
L_6a	PMC Left	0.2392	0.0000	0.0000
L_FEF	PMC Left	0.0391	0.0199	0.0336
R_6a	PMC Right	0.2006	0.0000	0.0000
L_AIP	SPC Left	0.1532	0.0000	0.0000
L_LIPd	SPC Left	0.0887	0.0000	0.0001
L_MIP	SPC Left	0.0805	0.0000	0.0001
R_AIP	SPC Right	0.1374	0.0000	0.0000
R_LIPd	SPC Right	0.1100	0.0000	0.0000
R_MIP	SPC Right	0.0567	0.0027	0.0052
L_p9-46v	DLPC Left	-0.0293	0.0168	0.0297
R_p9-46v	DLPC Right	-0.0277	0.0305	0.0493
L_44	IFC Left	-0.0558	0.0000	0.0000
R_p47r	IFC Right	-0.0381	0.0006	0.0013
R_44	IFC Right	-0.1006	0.0000	0.0000
L_FOP4	IFOC Left	-0.0393	0.0001	0.0003
R_FOP4	IFOC Right	-0.0807	0.0000	0.0000
L_PF	IPC Left	-0.0441	0.0047	0.0085
L_PGs	IPC Left	-0.0485	0.0029	0.0056
L_PFm	IPC Left	-0.0574	0.0001	0.0002

For Review Only

R_PFm	IPC Right	-0.0867	0.0000	0.0000
L_p10p	OPFC Left	-0.0698	0.0000	0.0000
R_a10p	OPFC Right	-0.0405	0.0246	0.0406
L_55b	PMC Left	-0.0931	0.0000	0.0000
R_55b	PMC Right	-0.0673	0.0000	0.0000
L_7Pm	SPC Left	-0.0755	0.0000	0.0000
L_7Am	SPC Left	-0.0918	0.0000	0.0000
L_7PL	SPC Left	-0.0520	0.0002	0.0005
R_7Am	SPC Right	-0.0830	0.0000	0.0000
R_7Pm	SPC Right	-0.0643	0.0005	0.0011
R_Caudate	Caudate Right	-0.1153	0.0000	0.0000
L_Caudate	Caudate Left	-0.1361	0.0000	0.0000
L_Putamen	Putamen Left	-0.0573	0.0000	0.0000
R_Putamen	Putamen Right	-0.0737	0.0000	0.0000
R_Thalamus	Thalamus Right	-0.0856	0.0000	0.0000
L_Thalamus	Thalamus Left	-0.0910	0.0000	0.0000

Supplementary Table 4. Statistical results for local assortativity (LA) at the 76-node scale. Only significant results are listed. Rows are ordered by estimate sign (positive first and then negative), followed by alphabetical order of anatomical region within each group. Columns are defined as in Supplementary Table 2. The Estimate column reflects the group mean difference (mean[HC] – mean[ALL]), with positive values indicating decreased LA in ALL survivors and negative values indicating increased LA in ALL survivors. All values are rounded to four decimal places.

Node	Anatomical Region	Estimate	Raw P-value	FDR P-value
L_d32	ACMPC Left	2.7314	0.0000	0.0000
R_d32	ACMPC Right	2.5325	0.0000	0.0000
R_46	DLPC Right	0.8395	0.0169	0.0389
L_IP1	IPC Left	1.1179	0.0025	0.0077
L_PGs	IPC Left	0.9274	0.0044	0.0121
R_IP1	IPC Right	1.0372	0.0047	0.0124
R_PF	IPC Right	1.4845	0.0012	0.0037
R_PGs	IPC Right	1.2661	0.0004	0.0015
L_a10p	OPFC Left	1.0657	0.0036	0.0101
R_11l	OPFC Right	1.2423	0.0030	0.0087
R_a10p	OPFC Right	1.2444	0.0004	0.0014
L_FEF	PMC Left	1.3780	0.0002	0.0009
L_6a	PMC Left	2.0648	0.0000	0.0000
R_6a	PMC Right	1.8844	0.0000	0.0000
L_7Pm	SPC Left	2.2952	0.0000	0.0000
L_7Am	SPC Left	2.8365	0.0000	0.0000
L_7PL	SPC Left	1.8294	0.0000	0.0002
L_AIP	SPC Left	0.9877	0.0063	0.0158
R_7PL	SPC Right	3.0937	0.0000	0.0000
R_AIP	SPC Right	1.3851	0.0002	0.0007
R_7Pm	SPC Right	3.2465	0.0000	0.0000

For Review Only

R_MIP	SPC Right	1.2174	0.0005	0.0018
R_LIPd	SPC Right	1.5559	0.0004	0.0015
R_7Am	SPC Right	3.5112	0.0000	0.0000
L_44	IFC Left	-0.8328	0.0131	0.0321
R_44	IFC Right	-1.1148	0.0003	0.0014
R_55b	PMC Right	-0.8749	0.0154	0.0366
L_Caudate	Caudate Left	-2.5725	0.0000	0.0000
R_Caudate	Caudate Right	-2.5022	0.0000	0.0000
L_Putamen	Putamen Left	-1.6060	0.0000	0.0000
R_Putamen	Putamen Right	-1.3175	0.0000	0.0001
R_Thalamus	Thalamus Right	-2.0584	0.0000	0.0000
L_Thalamus	Thalamus Left	-1.4466	0.0000	0.0000

Supplementary Table 5. Statistical results for participation coefficient (PC) at the 76-node scale. Only significant results are listed. Rows are ordered by estimate sign (positive first and then negative), followed by alphabetical order of anatomical region within each group. Columns are defined as in Supplementary Table 2. The Estimate column reflects the group mean difference (mean[HC] – mean[ALL]), with positive values indicating decreased PC in ALL survivors and negative values indicating increased PC in ALL survivors. All values are rounded to four decimal places.

Node	Anatomical Region	Estimate	Raw P-value	FDR P-value
R_8C	DLPC Right	0.0188	0.0259	0.0438
R_p9-46v	DLPC Right	0.0255	0.0151	0.0273
L_44	IFC Left	0.0279	0.0032	0.0067
R_p47r	IFC Right	0.0177	0.0078	0.0157
R_44	IFC Right	0.0606	0.0000	0.0000
L_FOP4	IFOC Left	0.0217	0.0202	0.0349
R_FOP5	IFOC Right	0.0273	0.0099	0.0192
R_FOP4	IFOC Right	0.0453	0.0000	0.0000
L_55b	PMC Left	0.0349	0.0001	0.0002
R_6r	PMC Right	0.0308	0.0001	0.0003
R_55b	PMC Right	0.0199	0.0128	0.0244
L_Caudate	Caudate Left	0. 1910	0.0000	0.0000
R_Caudate	Caudate Right	0. 1848	0.0000	0.0000
L_Putamen	Putamen Left	0.0637	0.0000	0.0000
R_Putamen	Putamen Right	0.0829	0.0000	0.0000
L_Thalamus	Thalamus Left	0.0863	0.0000	0.0000
R_Thalamus	Thalamus Right	0.0931	0.0000	0.0000
L_8BM	ACMPC Left	-0.0358	0.0023	0.0053
L_d32	ACMPC Left	-0.0754	0.0000	0.0000
R_d32	ACMPC Right	-0.0791	0.0000	0.0000
L_46	DLPC Left	-0.0233	0.0057	0.0116
L_9-46d	DLPC Left	-0.0457	0.0000	0.0000
R_s6-8	DLPC Right	-0.0261	0.0028	0.0062
R_9-46d	DLPC Right	-0.0219	0.0185	0.0327
R_46	DLPC Right	-0.0302	0.0024	0.0054
L_p47r	IFC Left	-0.0241	0.0014	0.0034
L_IFJp	IFC Left	-0.0330	0.0002	0.0006
R_IFJp	IFC Right	-0.0298	0.0005	0.0012

For Review Only

L_AVI	IFOC Left	-0.0225	0.0017	0.0039
L_IP1	IPC Left	-0.0218	0.0006	0.0016
L_IP2	IPC Left	-0.0433	0.0000	0.0000
R_IP2	IPC Right	-0.0315	0.0000	0.0000
R_IP1	IPC Right	-0.0285	0.0001	0.0003
L_a10p	OPFC Left	-0.0184	0.0007	0.0018
L_POS2	PCC Left	-0.0391	0.0000	0.0000
R_7m	PCC Right	-0.0164	0.0141	0.0261
R_POS2	PCC Right	-0.0338	0.0000	0.0000
L_6a	PMC Left	-0.1146	0.0000	0.0000
R_6a	PMC Right	-0.1006	0.0000	0.0000
L_MIP	SPC Left	-0.0495	0.0000	0.0000
L_LIPd	SPC Left	-0.0515	0.0000	0.0000
L_AIP	SPC Left	-0.0720	0.0000	0.0000
R_MIP	SPC Right	-0.0376	0.0000	0.0000
R_LIPd	SPC Right	-0.0446	0.0000	0.0000
R_AIP	SPC Right	-0.0674	0.0000	0.0000

Supplementary Table 6. Statistical results for clustering coefficient (CC) at the 24-node scale. Only significant results are listed. Rows are ordered by estimate sign (positive first and then negative), followed by alphabetical order of anatomical region within each group. Columns are defined as in Supplementary Table 2. The Estimate column reflects the group mean difference (mean[HC] – mean[ALL]), with positive values indicating decreased CC in ALL survivors and negative values indicating increased CC in ALL survivors. All values are rounded to four decimal places.

Node	Estimate	Raw P-value	FDR P-value
IFOC Left	0.0656	0.0000	0.0000
IFOC Right	0.1405	0.0000	0.0000
OPFC Left	0.0796	0.0000	0.0000
OPFC Right	0.1214	0.0000	0.0000
PCC Left	0.0496	0.0000	0.0000
PMC Left	0.0228	0.0220	0.0440
Thalamus Right	0.0559	0.0050	0.0108
IFC Left	-0.0678	0.0000	0.0000
IPC Left	-0.0452	0.0000	0.0000
PMC Right	-0.0387	0.0004	0.0010
Caudate Right	-0.0683	0.0000	0.0000
Putamen Left	-0.0319	0.0025	0.0059

Supplementary Table 7. Statistical results for eigenvector centrality (EC) at the 24-node scale. Only significant results are listed. Rows are ordered by estimate sign (positive first and then negative), followed by alphabetical order of anatomical region within each group. Columns are defined as in Supplementary Table 2. The Estimate column reflects the group mean difference (mean[HC] – mean[ALL]), with positive values indicating decreased EC in ALL survivors and negative values indicating increased EC in ALL survivors. All values are rounded to four decimal places.

Node	Estimate	Raw P-value	FDR P-value
IFC Left	0.0317	0.0000	0.0000
IFC Right	0.0070	0.0242	0.0323
IPC Left	0.0264	0.0000	0.0000

For Review Only

IPC Right	0.0107	0.0031	0.0057
PCC Right	0.0084	0.0063	0.0094
PMC Right	0.0239	0.0000	0.0000
Caudate Right	0.0301	0.0000	0.0000
Putamen Left	0.0109	0.0039	0.0067
Putamen Right	0.0176	0.0000	0.0001
Thalamus Left	0.0110	0.0156	0.0220
ACMPC Left	-0.0112	0.0024	0.0048
DLPC Left	-0.0106	0.0044	0.0070
DLPC Right	-0.0144	0.0015	0.0033
IFOC Left	-0.0283	0.0000	0.0000
IFOC Right	-0.0477	0.0000	0.0000
OPFC Left	-0.0281	0.0000	0.0000
OPFC Right	-0.0255	0.0000	0.0000
PCC Left	-0.0155	0.0000	0.0001

Supplementary Table 8. Statistical results for local assortativity (LA) at the 24-node scale. Only significant results are listed. Rows are ordered by estimate sign (positive first and then negative), followed by alphabetical order of anatomical region within each group. Columns are defined as in Supplementary Table 2. The Estimate column reflects the group mean difference (mean[HC] – mean[ALL]), with positive values indicating decreased LA in ALL survivors. All values are rounded to four decimal places.

Node	Estimate	Raw P-value	FDR P-value
ACMPC Left	0.5405	0.0018	0.0047
ACMPC Right	0.3842	0.0191	0.0328
DLPC Left	0.4755	0.0030	0.0060
DLPC Right	0.7121	0.0000	0.0001
IFC Right	0.8798	0.0000	0.0000
IFOC Left	0.3323	0.0273	0.0437
IFOC Right	0.5133	0.0018	0.0047
IPC Right	0.7559	0.0001	0.0006
OPFC Left	0.4260	0.0042	0.0078
OPFC Right	0.5225	0.0023	0.0050
PCC Left	0.5603	0.0012	0.0041
PMC Left	0.5881	0.0007	0.0028
PMC Right	0.3748	0.0297	0.0446
SPC Right	0.8694	0.0000	0.0000
Thalamus Left	0.5417	0.0019	0.0047
Thalamus Right	1.0753	0.0000	0.0000

Supplementary Table 9. Statistical results for participation coefficient (PC) at the 24-node scale. Only significant results are listed. Rows are ordered by estimate sign (positive first and then negative), followed by alphabetical order of anatomical region within each group. Columns are defined as in Supplementary Table 2. The Estimate column reflects the group mean difference (mean[HC] – mean[ALL]), with positive values indicating decreased PC in ALL survivors and negative values indicating increased PC in ALL survivors. All values are rounded to four decimal places.

Node	Estimate	Raw P-value	FDR P-value
ACMPC Left	0.0421	0.0028	0.0085

For Review Only

IFC Left	0.1283	0.0000	0.0000
IPC Left	0.0667	0.0005	0.0020
Caudate Right	0.1085	0.0000	0.0000
Putamen Right	0.0545	0.0010	0.0034
IFOC Right	-0.0788	0.0000	0.0000
OPFC Left	-0.0778	0.0000	0.0001
OPFC Right	-0.0596	0.0002	0.0010

For Review Only

Supplementary Figures

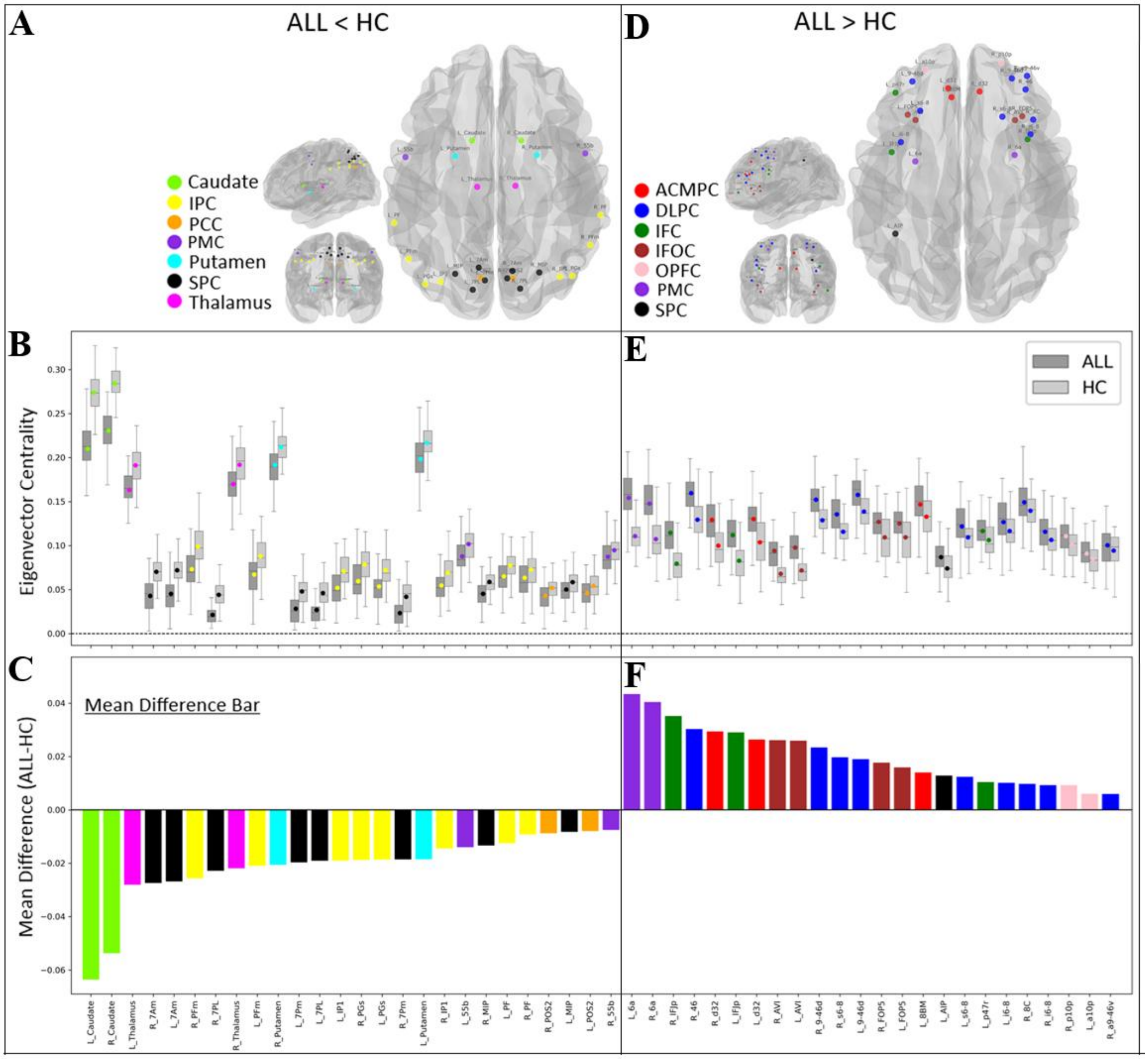

Supplementary Figure 1. Group differences in Eigenvector centrality (EC) across the 76-node working memory (WM) structural network. A-C show significant results where EC was lower in ALL survivors compared to healthy controls (HC) (ALL < HC), and D-F show significant results where EC was higher in ALL survivors (ALL > HC). A and D show glass brain plots highlighting significant nodes, with legend indicating color coded cortical and subcortical regions. B and E show boxplots illustrating groupwise distribution of EC values for each significant node for ALL and HC groups. C and F show bar plots displaying the mean difference (ALL − HC) in EC for each node, illustrating direction and magnitude of group effects. ACMPC – anterior cingulate medial prefrontal cortex, DLPC – dorsolateral prefrontal cortex, IFC – inferior frontal cortex, IFOC – inferior frontal occipital cortex, IPC – inferior parietal cortex, OPFC – orbitofrontal cortex, PCC – posterior cingulate cortex, PMC – premotor cortex, SPC – superior parietal cortex.

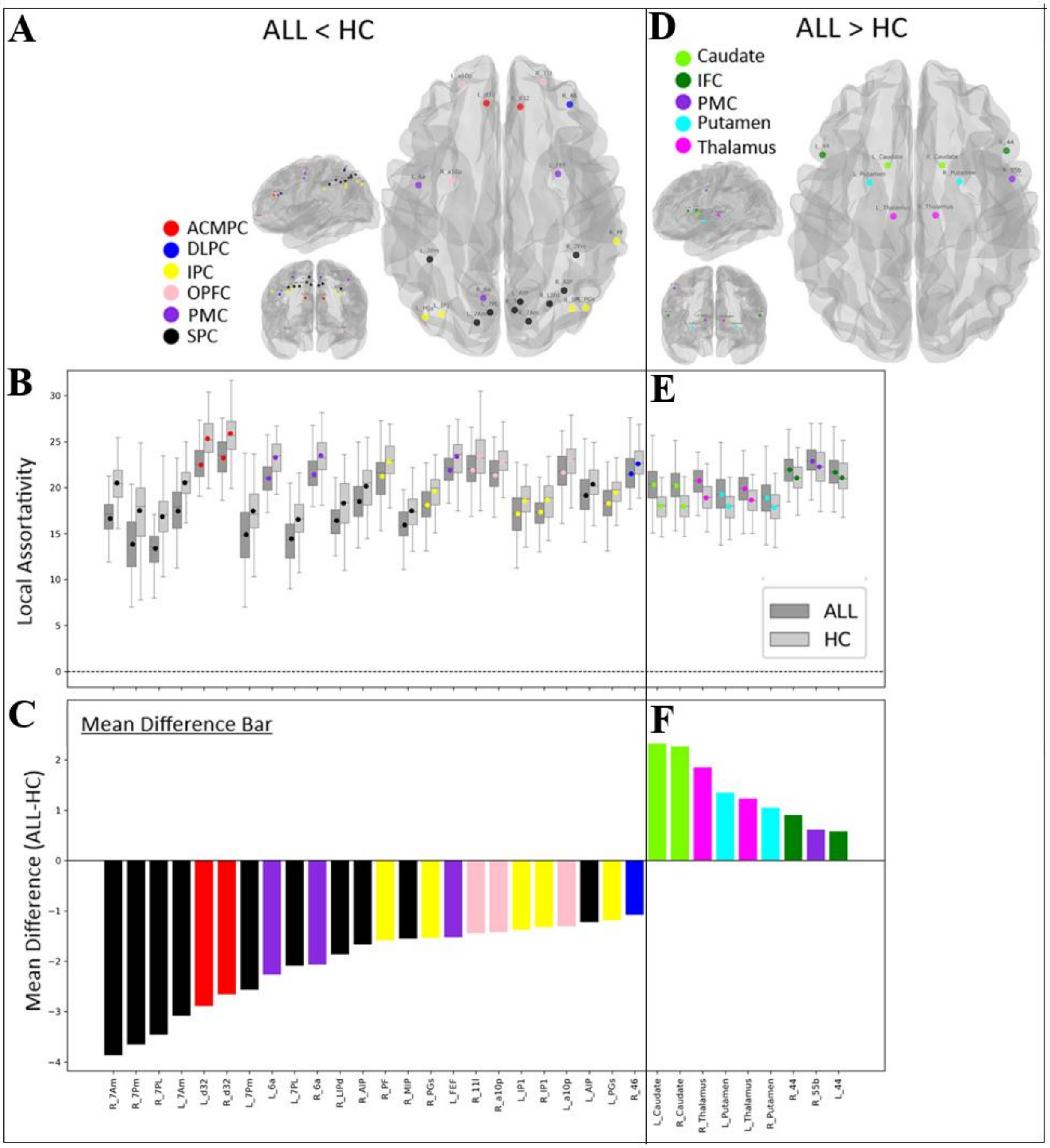

Supplementary Figure 2. Group differences in local assortativity (LA) across the 76-node working memory (WM) structural network. A-C show significant results where LA was lower in ALL survivors compared to healthy controls (HC) (ALL < HC), and D-F show significant results where LA was higher in ALL survivors (ALL > HC). A and D show glass brain plots highlighting significant nodes, with legend indicating color coded cortical and subcortical regions. B and E show boxplots illustrating groupwise distribution of LA values for each significant node in the ALL and HC groups. C and F show bar plots displaying the mean difference (ALL − HC) in LA for each node, illustrating the direction and magnitude of group effects. ACMPC – anterior cingulate medial prefrontal cortex, DLPC – dorsolateral prefrontal cortex, IFC – inferior frontal cortex, IPC – inferior parietal cortex, OPFC – orbitofrontal cortex, PMC – premotor cortex, SPC – superior parietal cortex.

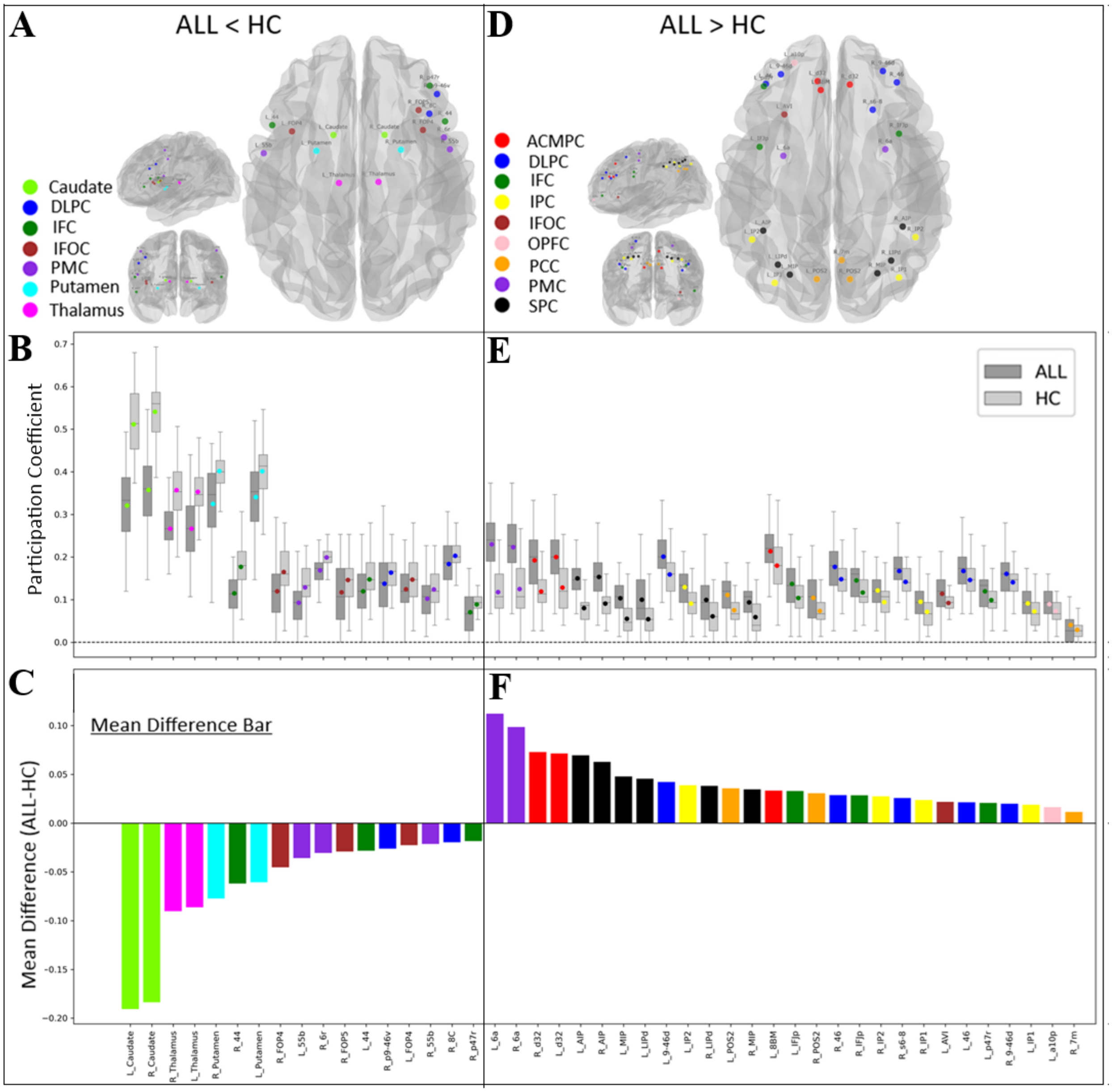

Supplementary Figure 3. Group differences in participation coefficient (PC) across the 76-node working memory (WM) structural network. A-C show significant results where PC was lower in ALL survivors compared to healthy controls (HC) (ALL < HC), and D-F show significant results where PC was higher in ALL survivors (ALL > HC). A and D show glass brain plots highlighting significant nodes, with legend indicating color coded cortical and subcortical regions. B and E show boxplots illustrating groupwise distribution of PC values for each significant node in the ALL and HC groups. C and F show bar plots displaying the mean difference (ALL − HC) in PC for each node, illustrating the direction and magnitude of group effects. ACMPC – anterior cingulate medial prefrontal cortex, DLPC – dorsolateral prefrontal cortex, IFC – inferior frontal cortex, IFOC – inferior frontal occipital cortex, IPC – inferior parietal cortex, OPFC – orbitofrontal cortex, PCC – posterior cingulate cortex, PMC – premotor cortex, SPC – superior parietal cortex.

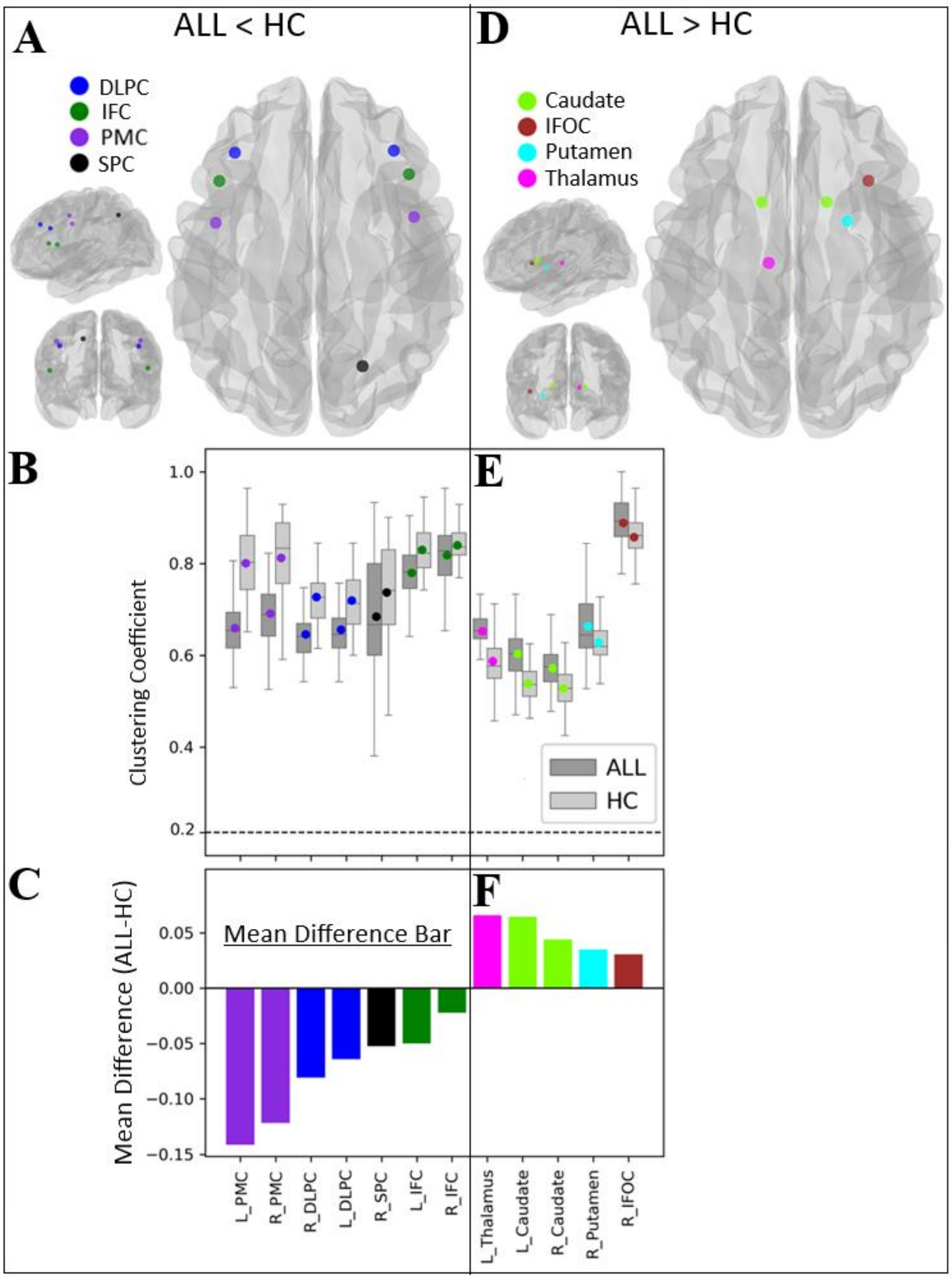

Supplementary Figure 4. Group differences in clustering coefficient (CC) across the 24-node working memory (WM) structural network. A-C show significant results where CC was lower in ALL survivors compared to healthy controls (HC) (ALL < HC), and D-F show significant results where CC was higher in ALL survivors (ALL > HC). A and D show glass brain plots highlighting significant nodes, with legend indicating color coded cortical and subcortical regions. B and E show boxplots illustrating groupwise distribution of CC values for each significant node in the ALL and HC groups. C and F show bar plots displaying the mean difference (ALL − HC) in CC for each node, illustrating the direction and magnitude of group effects. DLPC – dorsolateral prefrontal cortex, IFC – inferior frontal cortex, IFOC – inferior fronto occipital cortex, PMC – premotor cortex, SPC – superior parietal cortex.

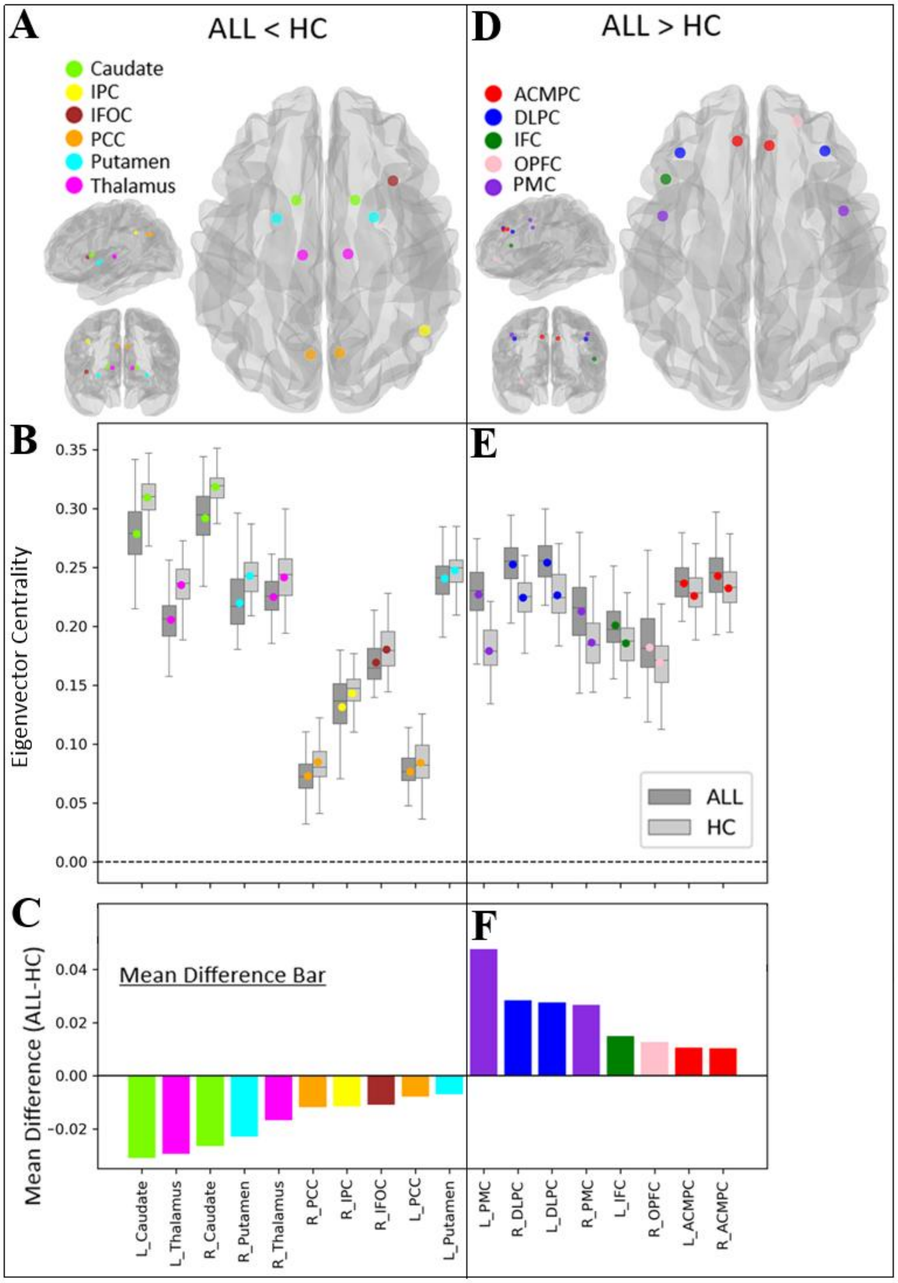

Supplementary Figure 5. Group differences in Eigenvector centrality (EC) across the 24-node working memory (WM) structural network. A-C show significant results where EC was lower in ALL survivors compared to healthy controls (HC) (ALL < HC), and D-F show regions where EC was higher in ALL survivors (ALL > HC). A and D show glass brain plots highlighting significant nodes, with legend indicating color coded cortical and subcortical regions. B and E show boxplots illustrating groupwise distribution of EC values for each significant node in the ALL and HC groups. C and F show bar plots displaying the mean difference (ALL − HC) in EC for each node, illustrating the direction and magnitude of group effects. ACMPC – anterior cingulate medial prefrontal cortex, DLPC – dorsolateral prefrontal cortex, IFC – inferior frontal cortex, IFOC – inferior fronto occipital cortex, IPC – inferior parietal cortex, OPFC – orbitofrontal cortex, PMC – premotor cortex, PCC – posterior cingulate cortex.

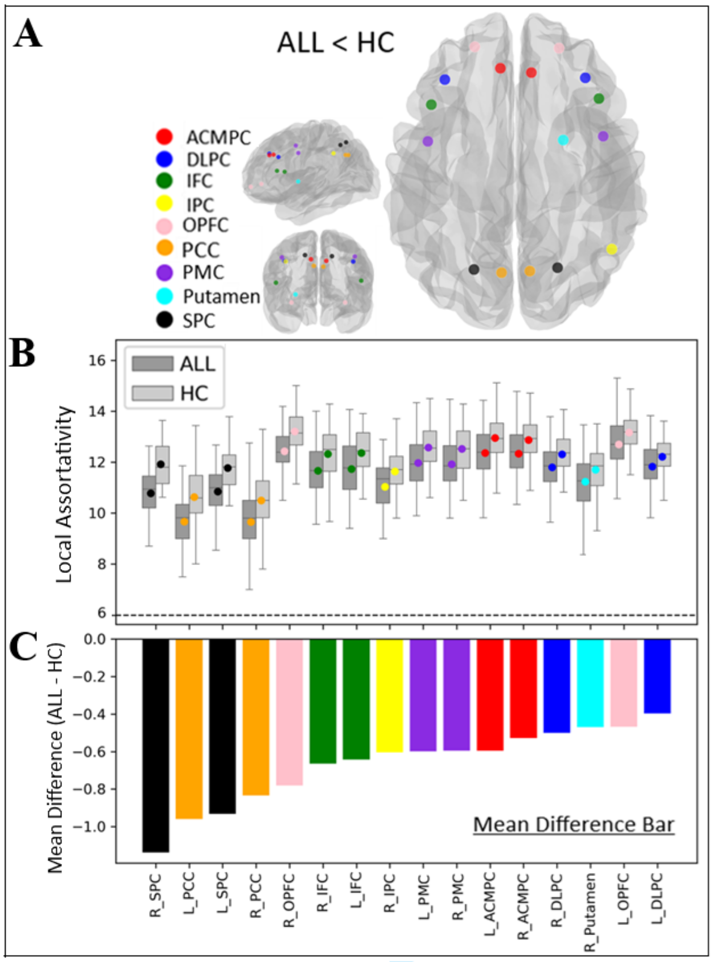

Supplementary Figure 6. Group differences in local assortativity (LA) across the 24-node working memory (WM) structural network. Figure shows significant results where LA was reduced in ALL survivors compared to healthy controls (HC) (ALL < HC); no regions exhibited significantly increased LA in the ALL group. A. Glass brain plots highlight significant nodes, with legend indicating color coded cortical and subcortical regions. B. Boxplots show groupwise distributions of LA values for each significant node in the ALL and HC groups. C. Bar plots display the mean difference (ALL − HC) in LA, illustrating the direction and magnitude of group effects. ACMPC – anterior cingulate medial prefrontal cortex, DLPC – dorsolateral prefrontal cortex, IFC – inferior frontal cortex, IPC – inferior parietal cortex, OPFC – orbitofrontal cortex, PCC – posterior cingulate cortex, PMC – premotor cortex, SPC – superior parietal cortex.

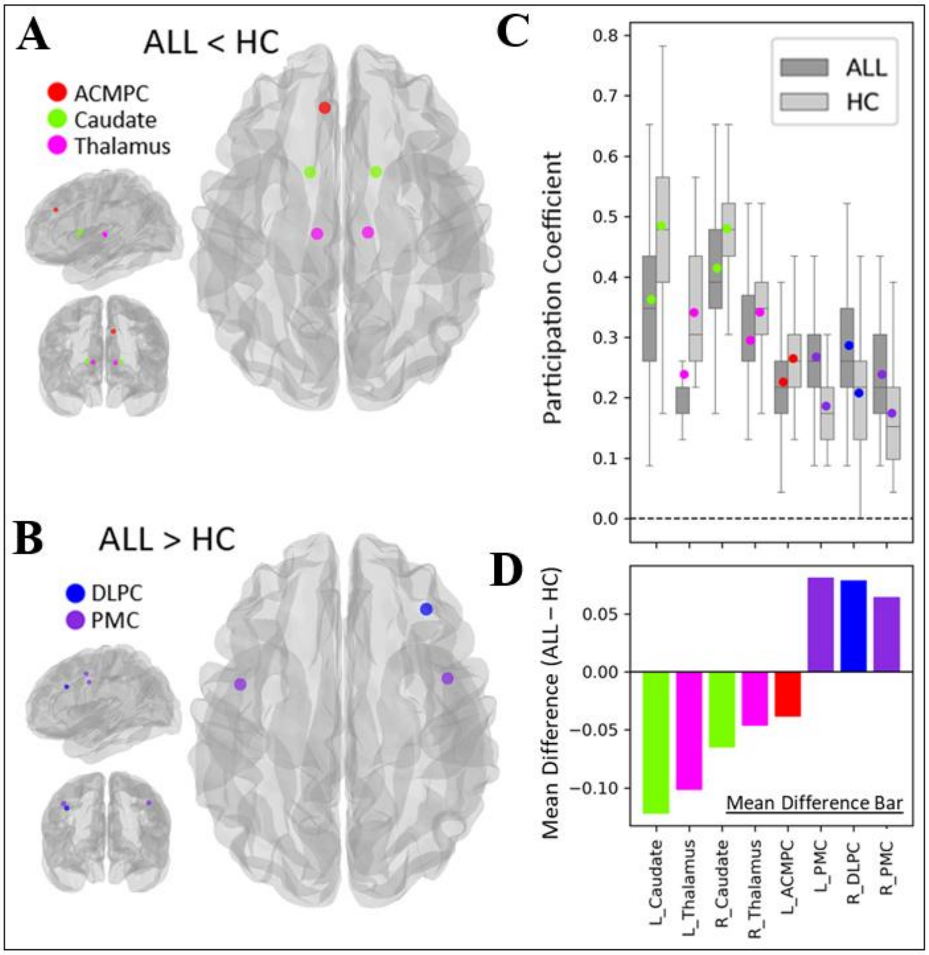

Supplementary Figure 7. Group differences in participation coefficient (PC) across the 24-node working memory (WM) structural network. A. Glass brain plot shows significant regions where PC was reduced in ALL survivors compared to healthy controls (HC) (ALL < HC). B. Glass brain plot shows regions where PC was increased in the ALL group (ALL > HC). C. Boxplots display the groupwise distribution of PC values for each significant node in the ALL and HC groups. D. Bar plots show the mean difference (ALL − HC) in PC for each node, illustrating the direction and magnitude of group effects. ACMPC – anterior cingulate medial prefrontal cortex, DLPC – dorsolateral prefrontal cortex, PMC – premotor cortex.

References

1. Rubinov M, Sporns O. Complex network measures of brain connectivity: Uses and interpretations. *Neuroimage*. 2010;52(3):1059-1069. doi:10.1016/j.neuroimage.2009.10.003

2. Hagberg AA, Schult DA, Swart PJ. Exploring Network Structure, Dynamics, and Function using NetworkX. In: *Proceedings of the 7th Python in Science Conference*. 2008. doi:10.25080/tcwv9851

3. Lohmann G, Margulies DS, Horstmann A, et al. Eigenvector centrality mapping for analyzing connectivity patterns in fMRI data of the human brain. *PLoS One*. 2010;5(4). doi:10.1371/journal.pone.0010232

4. Newman M. *Networks: An Introduction*. Oxford University Press; 2010. doi:10.1093/acprof:oso/9780199206650.001.0001

5. Newman MEJ. Assortative Mixing in Networks. *Phys Rev Lett*. 2002;89(20). doi:10.1103/PhysRevLett.89.208701

6. Watts DJ, Strogatz SH. Collective dynamics of 'small-world9 networks. *Nature*. 1998;393(6684). doi:10.1038/30918

7. Guimerà R, Amaral LAN. Functional cartography of complex metabolic networks. *Nature*. 2005;433(7028). doi:10.1038/nature03288

8. Bullmore E, Sporns O. Complex brain networks: graph theoretical analysis of structural and functional systems. *Nat Rev Neurosci*. 2009;10(3):186-198. doi:10.1038/nrn2575

9. Stam CJ, Jones BF, Nolte G, Breakspear M, Scheltens P. Small-world networks and functional connectivity in Alzheimer's disease. *Cerebral Cortex*. 2007;17(1). doi:10.1093/cercor/bhj127

10. Zuo XN, Ehmke R, Mennes M, et al. Network centrality in the human functional connectome. *Cerebral Cortex*. 2012;22(8). doi:10.1093/cercor/bhr269

11. Crossley NA, Mechelli A, Scott J, et al. The hubs of the human connectome are generally implicated in the anatomy of brain disorders. *Brain*. 2014;137(8). doi:10.1093/brain/awu132

12. Alexander-Bloch A, Lambiotte R, Roberts B, Giedd J, Gogtay N, Bullmore E. The discovery of population differences in network community structure: New methods and applications to brain functional networks in schizophrenia. *Neuroimage*. 2012;59(4). doi:10.1016/j.neuroimage.2011.11.035

13. Bertolero MA, Yeo BTT, D'Esposito M. The diverse club. *Nat Commun*. 2017;8(1). doi:10.1038/s41467-017-01189-w

14. van den Heuvel MP, Sporns O. Network hubs in the human brain. *Trends Cogn Sci*. 2013;17(12):683-696. doi:10.1016/j.tics.2013.09.012

For Review Only
