# Supplementary material for: Multiscale connectivity framework for working memory network in paediatric acute lymphoblastic leukaemia survivors
Source: Brain Commun. 2026 Apr 17;8(2):fcag137. doi: 10.1093/braincomms/fcag137 (PMC13126661; doi:10.1093/braincomms/fcag137)
Supplement: fcag137_Supplementary_Data [file fcag137_supplementary_data.zip › Supplementary_material.docx]

**Supplementary Materials**

**Multiscale Connectivity Framework for Working Memory Network in Pediatric Acute Lymphoblastic Leukemia Survivors**

**Supplementary Tables**

**Supplementary Table 1. Local network metrics used in the analysis.** Definitions are based on Brain Connectivity Toolbox^1^ and NetworkX,^2^ with interpretations informed by^3–7^

| Metric | Definition | Integration or Segregation | Impact of Higher Values in Human Brain Structural Connectivity | Impact of Lower Values in Human Brain Structural Connectivity |
| --- | --- | --- | --- | --- |
| Clustering Coefficient (CC) | Fraction of a node’s neighbors that are interconnected, indicating tightly knit local clusters.^1,6^ | Segregation | Indicates stronger local specialization and redundant short-range connectivity, supporting modular processing in healthy brains.^8^ | Reflects reduced local clustering and loss of specialization, often observed in disrupted or diseased structural networks.^9^ |
| Eigenvector Centrality (EC) | Measure of a node’s influence based on connections to other highly connected nodes.^3,4^ | Integration | Node functions as a global hub, enhancing integrative communication across distributed regions and supporting complex cognition.^3,10^ | Reduced hubness, with diminished contribution to global integration, associated with cognitive deficits in clinical populations.^11^ |
| Local Assortativity (LA) | Extent to which a node links to neighbors with similar levels of connectivity.^1,5^ | Segregation | Indicates preservation of modular segregation, with nodes linking to similarly connected neighbors, consistent with intact modular structure.^1^ | Reflects heterogeneous local wiring and reduced modularity, potentially observed in network disorganization in disease.^12^ |
| Participation Coefficient (PC) | Proportion of a node’s connections distributed across different modules, reflecting its role in cross-module integration.^1,7^ | Integration | Node bridges across modules, enabling cross-network integration and flexible communication.^7,13^ | Node is confined to within-module connections, limiting integration and possibly reflecting structural disconnection.^14^ |

**Supplementary Table 2. List of whole-brain cortical and subcortical parcels used in the 379-node structural connectivity matrices.** Parcels are defined according to the Human Connectome Project Multimodal Parcellation (HCP-MMP1.0) atlas and are listed in the ordering used for the 379 × 379 connectivity matrices shown in Figure 2B.

| Node | Region | Region Abbreviation | Hemisphere |
| --- | --- | --- | --- |
| L_10r | Anterior Cingulate and Medial Prefrontal Cortex Left | ACMPC Left | left |
| L_10v | Anterior Cingulate and Medial Prefrontal Cortex Left | ACMPC Left | left |
| L_25 | Anterior Cingulate and Medial Prefrontal Cortex Left | ACMPC Left | left |
| L_33pr | Anterior Cingulate and Medial Prefrontal Cortex Left | ACMPC Left | left |
| L_8BM | Anterior Cingulate and Medial Prefrontal Cortex Left | ACMPC Left | left |
| L_9m | Anterior Cingulate and Medial Prefrontal Cortex Left | ACMPC Left | left |
| L_a24 | Anterior Cingulate and Medial Prefrontal Cortex Left | ACMPC Left | left |
| L_a24pr | Anterior Cingulate and Medial Prefrontal Cortex Left | ACMPC Left | left |
| L_a32pr | Anterior Cingulate and Medial Prefrontal Cortex Left | ACMPC Left | left |
| L_d32 | Anterior Cingulate and Medial Prefrontal Cortex Left | ACMPC Left | left |
| L_p24 | Anterior Cingulate and Medial Prefrontal Cortex Left | ACMPC Left | left |
| L_p24pr | Anterior Cingulate and Medial Prefrontal Cortex Left | ACMPC Left | left |
| L_p32 | Anterior Cingulate and Medial Prefrontal Cortex Left | ACMPC Left | left |
| L_p32pr | Anterior Cingulate and Medial Prefrontal Cortex Left | ACMPC Left | left |
| L_s32 | Anterior Cingulate and Medial Prefrontal Cortex Left | ACMPC Left | left |
| L_A4 | Auditory Association Cortex Left | AAC Left | left |
| L_A5 | Auditory Association Cortex Left | AAC Left | left |
| L_STGa | Auditory Association Cortex Left | AAC Left | left |
| L_STSda | Auditory Association Cortex Left | AAC Left | left |
| L_STSdp | Auditory Association Cortex Left | AAC Left | left |
| L_STSva | Auditory Association Cortex Left | AAC Left | left |
| L_STSvp | Auditory Association Cortex Left | AAC Left | left |
| L_TA2 | Auditory Association Cortex Left | AAC Left | left |
| L_IPS1 | Dorsal Stream Visual Cortex Left | DSVC Left | left |
| L_V3A | Dorsal Stream Visual Cortex Left | DSVC Left | left |
| L_V3B | Dorsal Stream Visual Cortex Left | DSVC Left | left |
| L_V6 | Dorsal Stream Visual Cortex Left | DSVC Left | left |
| L_V6A | Dorsal Stream Visual Cortex Left | DSVC Left | left |
| L_V7 | Dorsal Stream Visual Cortex Left | DSVC Left | left |
| L_46 | DorsoLateral Prefrontal Cortex Left | DLPC Left | left |
| L_8Ad | DorsoLateral Prefrontal Cortex Left | DLPC Left | left |
| L_8Av | DorsoLateral Prefrontal Cortex Left | DLPC Left | left |
| L_8BL | DorsoLateral Prefrontal Cortex Left | DLPC Left | left |
| L_8C | DorsoLateral Prefrontal Cortex Left | DLPC Left | left |
| L_9-46d | DorsoLateral Prefrontal Cortex Left | DLPC Left | left |
| L_9a | DorsoLateral Prefrontal Cortex Left | DLPC Left | left |
| L_9p | DorsoLateral Prefrontal Cortex Left | DLPC Left | left |
| L_a9-46v | DorsoLateral Prefrontal Cortex Left | DLPC Left | left |
| L_i6-8 | DorsoLateral Prefrontal Cortex Left | DLPC Left | left |
| L_p9-46v | DorsoLateral Prefrontal Cortex Left | DLPC Left | left |
| L_s6-8 | DorsoLateral Prefrontal Cortex Left | DLPC Left | left |
| L_SFL | DorsoLateral Prefrontal Cortex Left | DLPC Left | left |
| L_A1 | Early Auditory Cortex Left | EAC Left | left |
| L_LBelt | Early Auditory Cortex Left | EAC Left | left |
| L_MBelt | Early Auditory Cortex Left | EAC Left | left |
| L_PBelt | Early Auditory Cortex Left | EAC Left | left |
| L_RI | Early Auditory Cortex Left | EAC Left | left |
| L_V2 | Early Visual Cortex Left | EVC Left | left |
| L_V3 | Early Visual Cortex Left | EVC Left | left |
| L_V4 | Early Visual Cortex Left | EVC Left | left |
| L_44 | Inferior Frontal Cortex Left | IFC Left | left |
| L_45 | Inferior Frontal Cortex Left | IFC Left | left |
| L_47l | Inferior Frontal Cortex Left | IFC Left | left |
| L_IFJa | Inferior Frontal Cortex Left | IFC Left | left |
| L_IFJp | Inferior Frontal Cortex Left | IFC Left | left |
| L_IFSa | Inferior Frontal Cortex Left | IFC Left | left |
| L_IFSp | Inferior Frontal Cortex Left | IFC Left | left |
| L_p47r | Inferior Frontal Cortex Left | IFC Left | left |
| L_IP0 | Inferior Parietal Cortex Left | IPC Left | left |
| L_IP1 | Inferior Parietal Cortex Left | IPC Left | left |
| L_IP2 | Inferior Parietal Cortex Left | IPC Left | left |
| L_PF | Inferior Parietal Cortex Left | IPC Left | left |
| L_PFm | Inferior Parietal Cortex Left | IPC Left | left |
| L_PFop | Inferior Parietal Cortex Left | IPC Left | left |
| L_PFt | Inferior Parietal Cortex Left | IPC Left | left |
| L_PGi | Inferior Parietal Cortex Left | IPC Left | left |
| L_PGp | Inferior Parietal Cortex Left | IPC Left | left |
| L_PGs | Inferior Parietal Cortex Left | IPC Left | left |
| L_52 | Insular and Frontal Opercular Cortex Left | IFOC Left | left |
| L_AAIC | Insular and Frontal Opercular Cortex Left | IFOC Left | left |
| L_AVI | Insular and Frontal Opercular Cortex Left | IFOC Left | left |
| L_FOP2 | Insular and Frontal Opercular Cortex Left | IFOC Left | left |
| L_FOP3 | Insular and Frontal Opercular Cortex Left | IFOC Left | left |
| L_FOP4 | Insular and Frontal Opercular Cortex Left | IFOC Left | left |
| L_FOP5 | Insular and Frontal Opercular Cortex Left | IFOC Left | left |
| L_Ig | Insular and Frontal Opercular Cortex Left | IFOC Left | left |
| L_MI | Insular and Frontal Opercular Cortex Left | IFOC Left | left |
| L_PI | Insular and Frontal Opercular Cortex Left | IFOC Left | left |
| L_Pir | Insular and Frontal Opercular Cortex Left | IFOC Left | left |
| L_PoI1 | Insular and Frontal Opercular Cortex Left | IFOC Left | left |
| L_PoI2 | Insular and Frontal Opercular Cortex Left | IFOC Left | left |
| L_PHT | Lateral Temporal Cortex Left | LTC Left | left |
| L_TE1a | Lateral Temporal Cortex Left | LTC Left | left |
| L_TE1m | Lateral Temporal Cortex Left | LTC Left | left |
| L_TE1p | Lateral Temporal Cortex Left | LTC Left | left |
| L_TE2a | Lateral Temporal Cortex Left | LTC Left | left |
| L_TE2p | Lateral Temporal Cortex Left | LTC Left | left |
| L_TF | Lateral Temporal Cortex Left | LTC Left | left |
| L_TGd | Lateral Temporal Cortex Left | LTC Left | left |
| L_TGv | Lateral Temporal Cortex Left | LTC Left | left |
| L_EC | Medial Temporal Cortex Left | MTC Left | left |
| L_H | Medial Temporal Cortex Left | MTC Left | left |
| L_Hippocampus | Medial Temporal Cortex Left | MTC Left | left |
| L_PeEc | Medial Temporal Cortex Left | MTC Left | left |
| L_PHA1 | Medial Temporal Cortex Left | MTC Left | left |
| L_PHA2 | Medial Temporal Cortex Left | MTC Left | left |
| L_PHA3 | Medial Temporal Cortex Left | MTC Left | left |
| L_PreS | Medial Temporal Cortex Left | MTC Left | left |
| L_FST | MT+ Complex and Neighboring Visual Areas Left | MTCNVA Left | left |
| L_LO1 | MT+ Complex and Neighboring Visual Areas Left | MTCNVA Left | left |
| L_LO2 | MT+ Complex and Neighboring Visual Areas Left | MTCNVA Left | left |
| L_LO3 | MT+ Complex and Neighboring Visual Areas Left | MTCNVA Left | left |
| L_MST | MT+ Complex and Neighboring Visual Areas Left | MTCNVA Left | left |
| L_MT | MT+ Complex and Neighboring Visual Areas Left | MTCNVA Left | left |
| L_PH | MT+ Complex and Neighboring Visual Areas Left | MTCNVA Left | left |
| L_V3CD | MT+ Complex and Neighboring Visual Areas Left | MTCNVA Left | left |
| L_V4t | MT+ Complex and Neighboring Visual Areas Left | MTCNVA Left | left |
| L_10d | Orbital and Polar Frontal Cortex Left | OPFC Left | left |
| L_10pp | Orbital and Polar Frontal Cortex Left | OPFC Left | left |
| L_11l | Orbital and Polar Frontal Cortex Left | OPFC Left | left |
| L_13l | Orbital and Polar Frontal Cortex Left | OPFC Left | left |
| L_47m | Orbital and Polar Frontal Cortex Left | OPFC Left | left |
| L_47s | Orbital and Polar Frontal Cortex Left | OPFC Left | left |
| L_a10p | Orbital and Polar Frontal Cortex Left | OPFC Left | left |
| L_a47r | Orbital and Polar Frontal Cortex Left | OPFC Left | left |
| L_OFC | Orbital and Polar Frontal Cortex Left | OPFC Left | left |
| L_p10p | Orbital and Polar Frontal Cortex Left | OPFC Left | left |
| L_pOFC | Orbital and Polar Frontal Cortex Left | OPFC Left | left |
| L_24dd | Paracentral Lobular and Mid Cingulate Cortex Left | PLMCC Left | left |
| L_24dv | Paracentral Lobular and Mid Cingulate Cortex Left | PLMCC Left | left |
| L_5L | Paracentral Lobular and Mid Cingulate Cortex Left | PLMCC Left | left |
| L_5m | Paracentral Lobular and Mid Cingulate Cortex Left | PLMCC Left | left |
| L_5mv | Paracentral Lobular and Mid Cingulate Cortex Left | PLMCC Left | left |
| L_6ma | Paracentral Lobular and Mid Cingulate Cortex Left | PLMCC Left | left |
| L_6mp | Paracentral Lobular and Mid Cingulate Cortex Left | PLMCC Left | left |
| L_SCEF | Paracentral Lobular and Mid Cingulate Cortex Left | PLMCC Left | left |
| L_23c | Posterior Cingulate Cortex Left | PCC Left | left |
| L_23d | Posterior Cingulate Cortex Left | PCC Left | left |
| L_31a | Posterior Cingulate Cortex Left | PCC Left | left |
| L_31pd | Posterior Cingulate Cortex Left | PCC Left | left |
| L_31pv | Posterior Cingulate Cortex Left | PCC Left | left |
| L_7m | Posterior Cingulate Cortex Left | PCC Left | left |
| L_d23ab | Posterior Cingulate Cortex Left | PCC Left | left |
| L_DVT | Posterior Cingulate Cortex Left | PCC Left | left |
| L_PCV | Posterior Cingulate Cortex Left | PCC Left | left |
| L_POS1 | Posterior Cingulate Cortex Left | PCC Left | left |
| L_POS2 | Posterior Cingulate Cortex Left | PCC Left | left |
| L_ProS | Posterior Cingulate Cortex Left | PCC Left | left |
| L_RSC | Posterior Cingulate Cortex Left | PCC Left | left |
| L_v23ab | Posterior Cingulate Cortex Left | PCC Left | left |
| L_43 | Posterior Opercular Cortex Left | POC Left | left |
| L_FOP1 | Posterior Opercular Cortex Left | POC Left | left |
| L_OP1 | Posterior Opercular Cortex Left | POC Left | left |
| L_OP2-3 | Posterior Opercular Cortex Left | POC Left | left |
| L_OP4 | Posterior Opercular Cortex Left | POC Left | left |
| L_PFcm | Posterior Opercular Cortex Left | POC Left | left |
| L_55b | Premotor Cortex Left | PMC Left | left |
| L_6a | Premotor Cortex Left | PMC Left | left |
| L_6d | Premotor Cortex Left | PMC Left | left |
| L_6r | Premotor Cortex Left | PMC Left | left |
| L_6v | Premotor Cortex Left | PMC Left | left |
| L_FEF | Premotor Cortex Left | PMC Left | left |
| L_PEF | Premotor Cortex Left | PMC Left | left |
| L_V1 | Primary Visual Cortex Left | PVC Left | left |
| L_1 | Somatosensory and Motor Cortex Left | SMC Left | left |
| L_2 | Somatosensory and Motor Cortex Left | SMC Left | left |
| L_3a | Somatosensory and Motor Cortex Left | SMC Left | left |
| L_3b | Somatosensory and Motor Cortex Left | SMC Left | left |
| L_4 | Somatosensory and Motor Cortex Left | SMC Left | left |
| L_7AL | Superior Parietal Cortex Left | SPC Left | left |
| L_7Am | Superior Parietal Cortex Left | SPC Left | left |
| L_7PC | Superior Parietal Cortex Left | SPC Left | left |
| L_7PL | Superior Parietal Cortex Left | SPC Left | left |
| L_7Pm | Superior Parietal Cortex Left | SPC Left | left |
| L_AIP | Superior Parietal Cortex Left | SPC Left | left |
| L_LIPd | Superior Parietal Cortex Left | SPC Left | left |
| L_LIPv | Superior Parietal Cortex Left | SPC Left | left |
| L_MIP | Superior Parietal Cortex Left | SPC Left | left |
| L_VIP | Superior Parietal Cortex Left | SPC Left | left |
| L_PSL | Temporo-Parieto-Occipital Junction Left | TPOJ Left | left |
| L_STV | Temporo-Parieto-Occipital Junction Left | TPOJ Left | left |
| L_TPOJ1 | Temporo-Parieto-Occipital Junction Left | TPOJ Left | left |
| L_TPOJ2 | Temporo-Parieto-Occipital Junction Left | TPOJ Left | left |
| L_TPOJ3 | Temporo-Parieto-Occipital Junction Left | TPOJ Left | left |
| L_FFC | Ventral Stream Visual Cortex Left | VSVC Left | left |
| L_PIT | Ventral Stream Visual Cortex Left | VSVC Left | left |
| L_V8 | Ventral Stream Visual Cortex Left | VSVC Left | left |
| L_VMV1 | Ventral Stream Visual Cortex Left | VSVC Left | left |
| L_VMV2 | Ventral Stream Visual Cortex Left | VSVC Left | left |
| L_VMV3 | Ventral Stream Visual Cortex Left | VSVC Left | left |
| L_VVC | Ventral Stream Visual Cortex Left | VSVC Left | left |
| L_Accumbens | Subcortical Left | SC Left | left |
| L_Amygdala | Subcortical Left | SC Left | left |
| L_Caudate | Subcortical Left | SC Left | left |
| L_Pallidum | Subcortical Left | SC Left | left |
| L_Putamen | Subcortical Left | SC Left | left |
| L_Thalamus | Subcortical Left | SC Left | left |
| L_VentralDC | Subcortical Left | SC Left | left |
| L_Cerebellum | Cerebellum Left | CB Left | left |
| R_10r | Anterior Cingulate and Medial Prefrontal Cortex Right | ACMPC Right | right |
| R_10v | Anterior Cingulate and Medial Prefrontal Cortex Right | ACMPC Right | right |
| R_25 | Anterior Cingulate and Medial Prefrontal Cortex Right | ACMPC Right | right |
| R_33pr | Anterior Cingulate and Medial Prefrontal Cortex Right | ACMPC Right | right |
| R_8BM | Anterior Cingulate and Medial Prefrontal Cortex Right | ACMPC Right | right |
| R_9m | Anterior Cingulate and Medial Prefrontal Cortex Right | ACMPC Right | right |
| R_a24 | Anterior Cingulate and Medial Prefrontal Cortex Right | ACMPC Right | right |
| R_a24pr | Anterior Cingulate and Medial Prefrontal Cortex Right | ACMPC Right | right |
| R_a32pr | Anterior Cingulate and Medial Prefrontal Cortex Right | ACMPC Right | right |
| R_d32 | Anterior Cingulate and Medial Prefrontal Cortex Right | ACMPC Right | right |
| R_p24 | Anterior Cingulate and Medial Prefrontal Cortex Right | ACMPC Right | right |
| R_p24pr | Anterior Cingulate and Medial Prefrontal Cortex Right | ACMPC Right | right |
| R_p32 | Anterior Cingulate and Medial Prefrontal Cortex Right | ACMPC Right | right |
| R_p32pr | Anterior Cingulate and Medial Prefrontal Cortex Right | ACMPC Right | right |
| R_s32 | Anterior Cingulate and Medial Prefrontal Cortex Right | ACMPC Right | right |
| R_A4 | Auditory Association Cortex Right | AAC Right | right |
| R_A5 | Auditory Association Cortex Right | AAC Right | right |
| R_STGa | Auditory Association Cortex Right | AAC Right | right |
| R_STSda | Auditory Association Cortex Right | AAC Right | right |
| R_STSdp | Auditory Association Cortex Right | AAC Right | right |
| R_STSva | Auditory Association Cortex Right | AAC Right | right |
| R_STSvp | Auditory Association Cortex Right | AAC Right | right |
| R_TA2 | Auditory Association Cortex Right | AAC Right | right |
| R_IPS1 | Dorsal Stream Visual Cortex Right | DSVC Right | right |
| R_V3A | Dorsal Stream Visual Cortex Right | DSVC Right | right |
| R_V3B | Dorsal Stream Visual Cortex Right | DSVC Right | right |
| R_V6 | Dorsal Stream Visual Cortex Right | DSVC Right | right |
| R_V6A | Dorsal Stream Visual Cortex Right | DSVC Right | right |
| R_V7 | Dorsal Stream Visual Cortex Right | DSVC Right | right |
| R_46 | DorsoLateral Prefrontal Cortex Right | DLPC Right | right |
| R_8Ad | DorsoLateral Prefrontal Cortex Right | DLPC Right | right |
| R_8Av | DorsoLateral Prefrontal Cortex Right | DLPC Right | right |
| R_8BL | DorsoLateral Prefrontal Cortex Right | DLPC Right | right |
| R_8C | DorsoLateral Prefrontal Cortex Right | DLPC Right | right |
| R_9-46d | DorsoLateral Prefrontal Cortex Right | DLPC Right | right |
| R_9a | DorsoLateral Prefrontal Cortex Right | DLPC Right | right |
| R_9p | DorsoLateral Prefrontal Cortex Right | DLPC Right | right |
| R_a9-46v | DorsoLateral Prefrontal Cortex Right | DLPC Right | right |
| R_i6-8 | DorsoLateral Prefrontal Cortex Right | DLPC Right | right |
| R_p9-46v | DorsoLateral Prefrontal Cortex Right | DLPC Right | right |
| R_s6-8 | DorsoLateral Prefrontal Cortex Right | DLPC Right | right |
| R_SFL | DorsoLateral Prefrontal Cortex Right | DLPC Right | right |
| R_A1 | Early Auditory Cortex Right | EAC Right | right |
| R_LBelt | Early Auditory Cortex Right | EAC Right | right |
| R_MBelt | Early Auditory Cortex Right | EAC Right | right |
| R_PBelt | Early Auditory Cortex Right | EAC Right | right |
| R_RI | Early Auditory Cortex Right | EAC Right | right |
| R_V2 | Early Visual Cortex Right | EVC Right | right |
| R_V3 | Early Visual Cortex Right | EVC Right | right |
| R_V4 | Early Visual Cortex Right | EVC Right | right |
| R_44 | Inferior Frontal Cortex Right | IFC Right | right |
| R_45 | Inferior Frontal Cortex Right | IFC Right | right |
| R_47l | Inferior Frontal Cortex Right | IFC Right | right |
| R_IFJa | Inferior Frontal Cortex Right | IFC Right | right |
| R_IFJp | Inferior Frontal Cortex Right | IFC Right | right |
| R_IFSa | Inferior Frontal Cortex Right | IFC Right | right |
| R_IFSp | Inferior Frontal Cortex Right | IFC Right | right |
| R_p47r | Inferior Frontal Cortex Right | IFC Right | right |
| R_IP0 | Inferior Parietal Cortex Right | IPC Right | right |
| R_IP1 | Inferior Parietal Cortex Right | IPC Right | right |
| R_IP2 | Inferior Parietal Cortex Right | IPC Right | right |
| R_PF | Inferior Parietal Cortex Right | IPC Right | right |
| R_PFm | Inferior Parietal Cortex Right | IPC Right | right |
| R_PFop | Inferior Parietal Cortex Right | IPC Right | right |
| R_PFt | Inferior Parietal Cortex Right | IPC Right | right |
| R_PGi | Inferior Parietal Cortex Right | IPC Right | right |
| R_PGp | Inferior Parietal Cortex Right | IPC Right | right |
| R_PGs | Inferior Parietal Cortex Right | IPC Right | right |
| R_52 | Insular and Frontal Opercular Cortex Right | IFOC Right | right |
| R_AAIC | Insular and Frontal Opercular Cortex Right | IFOC Right | right |
| R_AVI | Insular and Frontal Opercular Cortex Right | IFOC Right | right |
| R_FOP2 | Insular and Frontal Opercular Cortex Right | IFOC Right | right |
| R_FOP3 | Insular and Frontal Opercular Cortex Right | IFOC Right | right |
| R_FOP4 | Insular and Frontal Opercular Cortex Right | IFOC Right | right |
| R_FOP5 | Insular and Frontal Opercular Cortex Right | IFOC Right | right |
| R_Ig | Insular and Frontal Opercular Cortex Right | IFOC Right | right |
| R_MI | Insular and Frontal Opercular Cortex Right | IFOC Right | right |
| R_PI | Insular and Frontal Opercular Cortex Right | IFOC Right | right |
| R_Pir | Insular and Frontal Opercular Cortex Right | IFOC Right | right |
| R_PoI1 | Insular and Frontal Opercular Cortex Right | IFOC Right | right |
| R_PoI2 | Insular and Frontal Opercular Cortex Right | IFOC Right | right |
| R_PHT | Lateral Temporal Cortex Right | LTC Right | right |
| R_TE1a | Lateral Temporal Cortex Right | LTC Right | right |
| R_TE1m | Lateral Temporal Cortex Right | LTC Right | right |
| R_TE1p | Lateral Temporal Cortex Right | LTC Right | right |
| R_TE2a | Lateral Temporal Cortex Right | LTC Right | right |
| R_TE2p | Lateral Temporal Cortex Right | LTC Right | right |
| R_TF | Lateral Temporal Cortex Right | LTC Right | right |
| R_TGd | Lateral Temporal Cortex Right | LTC Right | right |
| R_TGv | Lateral Temporal Cortex Right | LTC Right | right |
| R_EC | Medial Temporal Cortex Right | MTC Right | right |
| R_H | Medial Temporal Cortex Right | MTC Right | right |
| R_Hippocampus | Medial Temporal Cortex Right | MTC Right | right |
| R_PeEc | Medial Temporal Cortex Right | MTC Right | right |
| R_PHA1 | Medial Temporal Cortex Right | MTC Right | right |
| R_PHA2 | Medial Temporal Cortex Right | MTC Right | right |
| R_PHA3 | Medial Temporal Cortex Right | MTC Right | right |
| R_PreS | Medial Temporal Cortex Right | MTC Right | right |
| R_FST | MT+ Complex and Neighboring Visual Areas Right | MTCNVA Right | right |
| R_LO1 | MT+ Complex and Neighboring Visual Areas Right | MTCNVA Right | right |
| R_LO2 | MT+ Complex and Neighboring Visual Areas Right | MTCNVA Right | right |
| R_LO3 | MT+ Complex and Neighboring Visual Areas Right | MTCNVA Right | right |
| R_MST | MT+ Complex and Neighboring Visual Areas Right | MTCNVA Right | right |
| R_MT | MT+ Complex and Neighboring Visual Areas Right | MTCNVA Right | right |
| R_PH | MT+ Complex and Neighboring Visual Areas Right | MTCNVA Right | right |
| R_V3CD | MT+ Complex and Neighboring Visual Areas Right | MTCNVA Right | right |
| R_V4t | MT+ Complex and Neighboring Visual Areas Right | MTCNVA Right | right |
| R_10d | Orbital and Polar Frontal Cortex Right | OPFC Right | right |
| R_10pp | Orbital and Polar Frontal Cortex Right | OPFC Right | right |
| R_11l | Orbital and Polar Frontal Cortex Right | OPFC Right | right |
| R_13l | Orbital and Polar Frontal Cortex Right | OPFC Right | right |
| R_47m | Orbital and Polar Frontal Cortex Right | OPFC Right | right |
| R_47s | Orbital and Polar Frontal Cortex Right | OPFC Right | right |
| R_a10p | Orbital and Polar Frontal Cortex Right | OPFC Right | right |
| R_a47r | Orbital and Polar Frontal Cortex Right | OPFC Right | right |
| R_OFC | Orbital and Polar Frontal Cortex Right | OPFC Right | right |
| R_p10p | Orbital and Polar Frontal Cortex Right | OPFC Right | right |
| R_pOFC | Orbital and Polar Frontal Cortex Right | OPFC Right | right |
| R_24dd | Paracentral Lobular and Mid Cingulate Cortex Right | PLMCC Right | right |
| R_24dv | Paracentral Lobular and Mid Cingulate Cortex Right | PLMCC Right | right |
| R_5L | Paracentral Lobular and Mid Cingulate Cortex Right | PLMCC Right | right |
| R_5m | Paracentral Lobular and Mid Cingulate Cortex Right | PLMCC Right | right |
| R_5mv | Paracentral Lobular and Mid Cingulate Cortex Right | PLMCC Right | right |
| R_6ma | Paracentral Lobular and Mid Cingulate Cortex Right | PLMCC Right | right |
| R_6mp | Paracentral Lobular and Mid Cingulate Cortex Right | PLMCC Right | right |
| R_SCEF | Paracentral Lobular and Mid Cingulate Cortex Right | PLMCC Right | right |
| R_23c | Posterior Cingulate Cortex Right | PCC Right | right |
| R_23d | Posterior Cingulate Cortex Right | PCC Right | right |
| R_31a | Posterior Cingulate Cortex Right | PCC Right | right |
| R_31pd | Posterior Cingulate Cortex Right | PCC Right | right |
| R_31pv | Posterior Cingulate Cortex Right | PCC Right | right |
| R_7m | Posterior Cingulate Cortex Right | PCC Right | right |
| R_d23ab | Posterior Cingulate Cortex Right | PCC Right | right |
| R_DVT | Posterior Cingulate Cortex Right | PCC Right | right |
| R_PCV | Posterior Cingulate Cortex Right | PCC Right | right |
| R_POS1 | Posterior Cingulate Cortex Right | PCC Right | right |
| R_POS2 | Posterior Cingulate Cortex Right | PCC Right | right |
| R_ProS | Posterior Cingulate Cortex Right | PCC Right | right |
| R_RSC | Posterior Cingulate Cortex Right | PCC Right | right |
| R_v23ab | Posterior Cingulate Cortex Right | PCC Right | right |
| R_43 | Posterior Opercular Cortex Right | POC Right | right |
| R_FOP1 | Posterior Opercular Cortex Right | POC Right | right |
| R_OP1 | Posterior Opercular Cortex Right | POC Right | right |
| R_OP2-3 | Posterior Opercular Cortex Right | POC Right | right |
| R_OP4 | Posterior Opercular Cortex Right | POC Right | right |
| R_PFcm | Posterior Opercular Cortex Right | POC Right | right |
| R_55b | Premotor Cortex Right | PMC Right | right |
| R_6a | Premotor Cortex Right | PMC Right | right |
| R_6d | Premotor Cortex Right | PMC Right | right |
| R_6r | Premotor Cortex Right | PMC Right | right |
| R_6v | Premotor Cortex Right | PMC Right | right |
| R_FEF | Premotor Cortex Right | PMC Right | right |
| R_PEF | Premotor Cortex Right | PMC Right | right |
| R_V1 | Primary Visual Cortex Right | PVC Right | right |
| R_1 | Somatosensory and Motor Cortex Right | SMC Right | right |
| R_2 | Somatosensory and Motor Cortex Right | SMC Right | right |
| R_3a | Somatosensory and Motor Cortex Right | SMC Right | right |
| R_3b | Somatosensory and Motor Cortex Right | SMC Right | right |
| R_4 | Somatosensory and Motor Cortex Right | SMC Right | right |
| R_7AL | Superior Parietal Cortex Right | SPC Right | right |
| R_7Am | Superior Parietal Cortex Right | SPC Right | right |
| R_7PC | Superior Parietal Cortex Right | SPC Right | right |
| R_7PL | Superior Parietal Cortex Right | SPC Right | right |
| R_7Pm | Superior Parietal Cortex Right | SPC Right | right |
| R_AIP | Superior Parietal Cortex Right | SPC Right | right |
| R_LIPd | Superior Parietal Cortex Right | SPC Right | right |
| R_LIPv | Superior Parietal Cortex Right | SPC Right | right |
| R_MIP | Superior Parietal Cortex Right | SPC Right | right |
| R_VIP | Superior Parietal Cortex Right | SPC Right | right |
| R_PSL | Temporo-Parieto-Occipital Junction Right | TPOJ Right | right |
| R_STV | Temporo-Parieto-Occipital Junction Right | TPOJ Right | right |
| R_TPOJ1 | Temporo-Parieto-Occipital Junction Right | TPOJ Right | right |
| R_TPOJ2 | Temporo-Parieto-Occipital Junction Right | TPOJ Right | right |
| R_TPOJ3 | Temporo-Parieto-Occipital Junction Right | TPOJ Right | right |
| R_FFC | Ventral Stream Visual Cortex Right | VSVC Right | right |
| R_PIT | Ventral Stream Visual Cortex Right | VSVC Right | right |
| R_V8 | Ventral Stream Visual Cortex Right | VSVC Right | right |
| R_VMV1 | Ventral Stream Visual Cortex Right | VSVC Right | right |
| R_VMV2 | Ventral Stream Visual Cortex Right | VSVC Right | right |
| R_VMV3 | Ventral Stream Visual Cortex Right | VSVC Right | right |
| R_VVC | Ventral Stream Visual Cortex Right | VSVC Right | right |
| R_Accumbens | Subcortical Right | SC Right | right |
| R_Amygdala | Subcortical Right | SC Right | right |
| R_Caudate | Subcortical Right | SC Right | right |
| R_Pallidum | Subcortical Right | SC Right | right |
| R_Putamen | Subcortical Right | SC Right | right |
| R_Thalamus | Subcortical Right | SC Right | right |
| R_VentralDC | Subcortical Right | SC Right | right |
| R_Cerebellum | Cerebellum Right | CB Right | right |
| Brain-Stem | Brain-Stem | BS |  |

**Supplementary Table 3. List of working memory (WM) associated parcels included in the fine-scale 76-node structural connectivity analysis.** Parcels are defined using the Human Connectome Project Multimodal Parcellation (HCP-MMP1.0) atlas and are listed in the ordering used for the 76 × 76 connectivity matrices shown in Figure 2D.

| Node | Region | Region Abbreviation | Hemisphere |
| --- | --- | --- | --- |
| L_8BM | Anterior Cingulate and Medial Prefrontal Cortex Left | ACMPC Left | left |
| L_d32 | Anterior Cingulate and Medial Prefrontal Cortex Left | ACMPC Left | left |
| L_46 | DorsoLateral Prefrontal Cortex Left | DLPC Left | left |
| L_8C | DorsoLateral Prefrontal Cortex Left | DLPC Left | left |
| L_9-46d | DorsoLateral Prefrontal Cortex Left | DLPC Left | left |
| L_a9-46v | DorsoLateral Prefrontal Cortex Left | DLPC Left | left |
| L_i6-8 | DorsoLateral Prefrontal Cortex Left | DLPC Left | left |
| L_p9-46v | DorsoLateral Prefrontal Cortex Left | DLPC Left | left |
| L_s6-8 | DorsoLateral Prefrontal Cortex Left | DLPC Left | left |
| L_44 | Inferior Frontal Cortex Left | IFC Left | left |
| L_IFJp | Inferior Frontal Cortex Left | IFC Left | left |
| L_p47r | Inferior Frontal Cortex Left | IFC Left | left |
| L_IP1 | Inferior Parietal Cortex Left | IPC Left | left |
| L_IP2 | Inferior Parietal Cortex Left | IPC Left | left |
| L_PF | Inferior Parietal Cortex Left | IPC Left | left |
| L_PFm | Inferior Parietal Cortex Left | IPC Left | left |
| L_PGs | Inferior Parietal Cortex Left | IPC Left | left |
| L_AVI | Insular and Frontal Opercular Cortex Left | IFOC Left | left |
| L_FOP4 | Insular and Frontal Opercular Cortex Left | IFOC Left | left |
| L_FOP5 | Insular and Frontal Opercular Cortex Left | IFOC Left | left |
| L_11l | Orbital and Polar Frontal Cortex Left | OPFC Left | left |
| L_a10p | Orbital and Polar Frontal Cortex Left | OPFC Left | left |
| L_p10p | Orbital and Polar Frontal Cortex Left | OPFC Left | left |
| L_7m | Posterior Cingulate Cortex Left | PCC Left | left |
| L_POS2 | Posterior Cingulate Cortex Left | PCC Left | left |
| L_55b | Premotor Cortex Left | PMC Left | left |
| L_6a | Premotor Cortex Left | PMC Left | left |
| L_6r | Premotor Cortex Left | PMC Left | left |
| L_FEF | Premotor Cortex Left | PMC Left | left |
| L_7Am | Superior Parietal Cortex Left | SPC Left | left |
| L_7PL | Superior Parietal Cortex Left | SPC Left | left |
| L_7Pm | Superior Parietal Cortex Left | SPC Left | left |
| L_AIP | Superior Parietal Cortex Left | SPC Left | left |
| L_LIPd | Superior Parietal Cortex Left | SPC Left | left |
| L_MIP | Superior Parietal Cortex Left | SPC Left | left |
| L_Thalamus | Thalamus Left | Thalamus Left | left |
| L_Caudate | Caudate Left | Caudate Left | left |
| L_Putamen | Putamen Left | Putamen Left | left |
| R_d32 | Anterior Cingulate and Medial Prefrontal Cortex Right | ACMPC Right | right |
| R_8BM | Anterior Cingulate and Medial Prefrontal Cortex Right | ACMPC Right | right |
| R_8C | DorsoLateral Prefrontal Cortex Right | DLPC Right | right |
| R_p9-46v | DorsoLateral Prefrontal Cortex Right | DLPC Right | right |
| R_46 | DorsoLateral Prefrontal Cortex Right | DLPC Right | right |
| R_a9-46v | DorsoLateral Prefrontal Cortex Right | DLPC Right | right |
| R_9-46d | DorsoLateral Prefrontal Cortex Right | DLPC Right | right |
| R_i6-8 | DorsoLateral Prefrontal Cortex Right | DLPC Right | right |
| R_s6-8 | DorsoLateral Prefrontal Cortex Right | DLPC Right | right |
| R_44 | Inferior Frontal Cortex Right | IFC Right | right |
| R_IFJp | Inferior Frontal Cortex Right | IFC Right | right |
| R_p47r | Inferior Frontal Cortex Right | IFC Right | right |
| R_IP2 | Inferior Parietal Cortex Right | IPC Right | right |
| R_IP1 | Inferior Parietal Cortex Right | IPC Right | right |
| R_PF | Inferior Parietal Cortex Right | IPC Right | right |
| R_PFm | Inferior Parietal Cortex Right | IPC Right | right |
| R_PGs | Inferior Parietal Cortex Right | IPC Right | right |
| R_FOP4 | Insular and Frontal Opercular Cortex Right | IFOC Right | right |
| R_AVI | Insular and Frontal Opercular Cortex Right | IFOC Right | right |
| R_FOP5 | Insular and Frontal Opercular Cortex Right | IFOC Right | right |
| R_a10p | Orbital and Polar Frontal Cortex Right | OPFC Right | right |
| R_11l | Orbital and Polar Frontal Cortex Right | OPFC Right | right |
| R_p10p | Orbital and Polar Frontal Cortex Right | OPFC Right | right |
| R_POS2 | Posterior Cingulate Cortex Right | PCC Right | right |
| R_7m | Posterior Cingulate Cortex Right | PCC Right | right |
| R_FEF | Premotor Cortex Right | PMC Right | right |
| R_55b | Premotor Cortex Right | PMC Right | right |
| R_6r | Premotor Cortex Right | PMC Right | right |
| R_6a | Premotor Cortex Right | PMC Right | right |
| R_7Pm | Superior Parietal Cortex Right | SPC Right | right |
| R_7Am | Superior Parietal Cortex Right | SPC Right | right |
| R_7PL | Superior Parietal Cortex Right | SPC Right | right |
| R_MIP | Superior Parietal Cortex Right | SPC Right | right |
| R_LIPd | Superior Parietal Cortex Right | SPC Right | right |
| R_AIP | Superior Parietal Cortex Right | SPC Right | right |
| R_Thalamus | Thalamus Right | Thalamus Right | right |
| R_Caudate | Caudate Right | Caudate Right | right |
| R_Putamen | Putamen Right | Putamen Right | right |

**Supplementary Table 4. List of working memory (WM) associated parcels included in the coarse-scale 24-node structural connectivity analysis.** Parcels are defined using the Human Connectome Project Multimodal Parcellation (HCP-MMP1.0) atlas and are listed in the ordering used for the 24 × 24 connectivity matrices shown in Figure 2F.

| Region | Region Abbreviation | Hemisphere |
| --- | --- | --- |
| Anterior Cingulate and Medial Prefrontal Cortex Left | ACMPC Left | left |
| DorsoLateral Prefrontal Cortex Left | DLPC Left | left |
| Inferior Frontal Cortex Left | IFC Left | left |
| Inferior Parietal Cortex Left | IPC Left | left |
| Insular and Frontal Opercular Cortex Left | IFOC Left | left |
| Orbital and Polar Frontal Cortex Left | OPFC Left | left |
| Posterior Cingulate Cortex Left | PCC Left | left |
| Premotor Cortex Left | PMC Left | left |
| Superior Parietal Cortex Left | SPC Left | left |
| Thalamus Left | Thalamus Left | left |
| Caudate Left | Caudate Left | left |
| Putamen Left | Putamen Left | left |
| Anterior Cingulate and Medial Prefrontal Cortex Right | ACMPC Right | right |
| DorsoLateral Prefrontal Cortex Right | DLPC Right | right |
| Inferior Frontal Cortex Right | IFC Right | right |
| Inferior Parietal Cortex Right | IPC Right | right |
| Insular and Frontal Opercular Cortex Right | IFOC Right | right |
| Orbital and Polar Frontal Cortex Right | OPFC Right | right |
| Posterior Cingulate Cortex Right | PCC Right | right |
| Premotor Cortex Right | PMC Right | right |
| Superior Parietal Cortex Right | SPC Right | right |
| Thalamus Right | Thalamus Right | right |
| Caudate Right | Caudate Right | right |
| Putamen Right | Putamen Right | right |

**Supplementary Table 5. Statistical results for clustering coefficient (CC) at the 76-node scale, corresponding to the significant group differences shown in Figure 3 and underlying the summary pattern shown in Figure 6A.** Only significant results are listed. Rows are ordered by estimate sign (positive first and then negative), followed by alphabetical order of anatomical region within each group. Columns are defined as follows: Node – fine-scale parcellation label, with prefixes L_ and R_ indicating left and right hemisphere nodes, respectively; Anatomical Region – broader cortical or subcortical grouping; Estimate – group mean difference (contrast: mean[HC] – mean[ALL]), where positive values indicate decreased CC in ALL survivors (ALL < HC) and negative values indicate increased CC in ALL survivors (ALL > HC); Raw P-value – uncorrected p-value; FDR P-value – false discovery rate adjusted P-value. All values are rounded to four decimal places.

| **Node** | **Anatomical Region** | **Estimate** | **Raw P-value** | **FDR P-value** |
| --- | --- | --- | --- | --- |
| L_d32 | ACMPC Left | 0.0778 | 0.0000 | 0.0000 |
| R_d32 | ACMPC Right | 0.0993 | 0.0000 | 0.0000 |
| L_s6-8 | DLPC Left | 0.0693 | 0.0000 | 0.0000 |
| L_9-46d | DLPC Left | 0.0514 | 0.0000 | 0.0001 |
| L_i6-8 | DLPC Left | 0.0497 | 0.0039 | 0.0073 |
| R_s6-8 | DLPC Right | 0.0928 | 0.0000 | 0.0000 |
| R_46 | DLPC Right | 0.0872 | 0.0000 | 0.0000 |
| R_9-46d | DLPC Right | 0.0500 | 0.0000 | 0.0000 |
| L_IFJp | IFC Left | 0.1007 | 0.0000 | 0.0000 |
| R_IFJp | IFC Right | 0.0994 | 0.0000 | 0.0000 |
| L_IP2 | IPC Left | 0.0600 | 0.0000 | 0.0001 |
| R_IP2 | IPC Right | 0.0314 | 0.0191 | 0.0331 |
| L_6a | PMC Left | 0.2392 | 0.0000 | 0.0000 |
| L_FEF | PMC Left | 0.0391 | 0.0199 | 0.0336 |
| R_6a | PMC Right | 0.2006 | 0.0000 | 0.0000 |
| L_AIP | SPC Left | 0.1532 | 0.0000 | 0.0000 |
| L_LIPd | SPC Left | 0.0887 | 0.0000 | 0.0001 |
| L_MIP | SPC Left | 0.0805 | 0.0000 | 0.0001 |
| R_AIP | SPC Right | 0.1374 | 0.0000 | 0.0000 |
| R_LIPd | SPC Right | 0.1100 | 0.0000 | 0.0000 |
| R_MIP | SPC Right | 0.0567 | 0.0027 | 0.0052 |
| L_p9-46v | DLPC Left | -0.0293 | 0.0168 | 0.0297 |
| R_p9-46v | DLPC Right | -0.0277 | 0.0305 | 0.0493 |
| L_44 | IFC Left | -0.0558 | 0.0000 | 0.0000 |
| R_p47r | IFC Right | -0.0381 | 0.0006 | 0.0013 |
| R_44 | IFC Right | -0.1006 | 0.0000 | 0.0000 |
| L_FOP4 | IFOC Left | -0.0393 | 0.0001 | 0.0003 |
| R_FOP4 | IFOC Right | -0.0807 | 0.0000 | 0.0000 |
| L_PF | IPC Left | -0.0441 | 0.0047 | 0.0085 |
| L_PGs | IPC Left | -0.0485 | 0.0029 | 0.0056 |
| L_PFm | IPC Left | -0.0574 | 0.0001 | 0.0002 |
| R_PFm | IPC Right | -0.0867 | 0.0000 | 0.0000 |
| L_p10p | OPFC Left | -0.0698 | 0.0000 | 0.0000 |
| R_a10p | OPFC Right | -0.0405 | 0.0246 | 0.0406 |
| L_55b | PMC Left | -0.0931 | 0.0000 | 0.0000 |
| R_55b | PMC Right | -0.0673 | 0.0000 | 0.0000 |
| L_7Pm | SPC Left | -0.0755 | 0.0000 | 0.0000 |
| L_7Am | SPC Left | -0.0918 | 0.0000 | 0.0000 |
| L_7PL | SPC Left | -0.0520 | 0.0002 | 0.0005 |
| R_7Am | SPC Right | -0.0830 | 0.0000 | 0.0000 |
| R_7Pm | SPC Right | -0.0643 | 0.0005 | 0.0011 |
| R_Caudate | Caudate Right | -0.1153 | 0.0000 | 0.0000 |
| L_Caudate | Caudate Left | -0.1361 | 0.0000 | 0.0000 |
| L_Putamen | Putamen Left | -0.0573 | 0.0000 | 0.0000 |
| R_Putamen | Putamen Right | -0.0737 | 0.0000 | 0.0000 |
| R_Thalamus | Thalamus Right | -0.0856 | 0.0000 | 0.0000 |
| L_Thalamus | Thalamus Left | -0.0910 | 0.0000 | 0.0000 |

**Supplementary Table 6. Statistical results for Eigenvector centrality (EC) at the 76-node scale, corresponding to the significant group differences shown in Figure 4A-B and underlying the summary pattern shown in Figure 6B.** Only significant results are listed. Rows are ordered by estimate sign (positive first and then negative), followed by alphabetical order of anatomical region within each group. Columns are defined as in Supplementary Table 2. The Estimate column reflects the group mean difference (mean[HC] – mean[ALL]), with positive values indicating decreased EC in ALL survivors and negative values indicating increased EC in ALL survivors. All values are rounded to four decimal places.

| **Node** | **Anatomical Region** | **Estimate** | **Raw P-value** | **FDR P-value** |
| --- | --- | --- | --- | --- |
| L_IP1 | IPC Left | 0.0182 | 0.0000 | 0.0000 |
| L_PF | IPC Left | 0.0123 | 0.0003 | 0.0007 |
| L_PFm | IPC Left | 0.0203 | 0.0000 | 0.0000 |
| L_PGs | IPC Left | 0.0185 | 0.0000 | 0.0000 |
| R_IP1 | IPC Right | 0.0128 | 0.0001 | 0.0003 |
| R_PF | IPC Right | 0.0087 | 0.0188 | 0.0301 |
| R_PFm | IPC Right | 0.0254 | 0.0000 | 0.0000 |
| R_PGs | IPC Right | 0.0176 | 0.0000 | 0.0000 |
| L_POS2 | PCC Left | 0.0071 | 0.0041 | 0.0075 |
| R_POS2 | PCC Right | 0.0077 | 0.0030 | 0.0058 |
| L_55b | PMC Left | 0.0144 | 0.0000 | 0.0000 |
| R_55b | PMC Right | 0.0080 | 0.0141 | 0.0238 |
| L_7Am | SPC Left | 0.0267 | 0.0000 | 0.0000 |
| L_7PL | SPC Left | 0.0192 | 0.0000 | 0.0000 |
| L_7Pm | SPC Left | 0.0195 | 0.0000 | 0.0000 |
| L_MIP | SPC Left | 0.0079 | 0.0109 | 0.0189 |
| R_7Am | SPC Right | 0.0267 | 0.0000 | 0.0000 |
| R_7PL | SPC Right | 0.0221 | 0.0000 | 0.0000 |
| R_7Pm | SPC Right | 0.0172 | 0.0000 | 0.0000 |
| R_MIP | SPC Right | 0.0123 | 0.0000 | 0.0000 |
| L_Caudate | Caudate Left | 0.0647 | 0.0000 | 0.0000 |
| R_Caudate | Caudate Right | 0.0545 | 0.0000 | 0.0000 |
| L_Putamen | Putamen Left | 0.0199 | 0.0000 | 0.0000 |
| R_Putamen | Putamen Right | 0.0222 | 0.0000 | 0.0000 |
| L_Thalamus | Thalamus Left | 0.0283 | 0.0000 | 0.0000 |
| R_Thalamus | Thalamus Right | 0.0226 | 0.0000 | 0.0000 |
| L_8BM | ACMPC Left | -0.0156 | 0.0024 | 0.0048 |
| L_d32 | ACMPC Left | -0.0282 | 0.0000 | 0.0000 |
| R_d32 | ACMPC Right | -0.0307 | 0.0000 | 0.0000 |
| L_i6-8 | DLPC Left | -0.0100 | 0.0190 | 0.0301 |
| L_s6-8 | DLPC Left | -0.0122 | 0.0015 | 0.0030 |
| L_9-46d | DLPC Left | -0.0191 | 0.0000 | 0.0000 |
| R_a9-46v | DLPC Right | -0.0072 | 0.0291 | 0.0452 |
| R_i6-8 | DLPC Right | -0.0085 | 0.0098 | 0.0174 |
| R_8C | DLPC Right | -0.0092 | 0.0165 | 0.0272 |
| R_s6-8 | DLPC Right | -0.0186 | 0.0000 | 0.0000 |
| R_9-46d | DLPC Right | -0.0231 | 0.0000 | 0.0000 |
| R_46 | DLPC Right | -0.0295 | 0.0000 | 0.0000 |
| L_p47r | IFC Left | -0.0112 | 0.0039 | 0.0075 |
| L_IFJp | IFC Left | -0.0290 | 0.0000 | 0.0000 |
| R_IFJp | IFC Right | -0.0348 | 0.0000 | 0.0000 |
| L_FOP5 | IFOC Left | -0.0169 | 0.0004 | 0.0008 |
| L_AVI | IFOC Left | -0.0260 | 0.0000 | 0.0000 |
| R_FOP5 | IFOC Right | -0.0177 | 0.0002 | 0.0004 |
| R_AVI | IFOC Right | -0.0261 | 0.0000 | 0.0000 |
| L_a10p | OPFC Left | -0.0068 | 0.0303 | 0.0461 |
| R_p10p | OPFC Right | -0.0097 | 0.0042 | 0.0075 |
| L_6a | PC Left | -0.0439 | 0.0000 | 0.0000 |
| R_6a | PC Right | -0.0395 | 0.0000 | 0.0000 |
| L_AIP | SPC Left | -0.0133 | 0.0001 | 0.0003 |

**Supplementary Table 7. Statistical results for local assortativity (LA) at the 76-node scale, corresponding to the significant group differences shown in Figure 4C-D and underlying the summary pattern shown in Figure 6C.** Only significant results are listed. Rows are ordered by estimate sign (positive first and then negative), followed by alphabetical order of anatomical region within each group. Columns are defined as in Supplementary Table 2. The Estimate column reflects the group mean difference (mean[HC] – mean[ALL]), with positive values indicating decreased LA in ALL survivors and negative values indicating increased LA in ALL survivors. All values are rounded to four decimal places.

| **Node** | **Anatomical Region** | **Estimate** | **Raw P-value** | **FDR P-value** |
| --- | --- | --- | --- | --- |
| L_d32 | ACMPC Left | 2.7314 | 0.0000 | 0.0000 |
| R_d32 | ACMPC Right | 2.5325 | 0.0000 | 0.0000 |
| R_46 | DLPC Right | 0.8395 | 0.0169 | 0.0389 |
| L_IP1 | IPC Left | 1.1179 | 0.0025 | 0.0077 |
| L_PGs | IPC Left | 0.9274 | 0.0044 | 0.0121 |
| R_IP1 | IPC Right | 1.0372 | 0.0047 | 0.0124 |
| R_PF | IPC Right | 1.4845 | 0.0012 | 0.0037 |
| R_PGs | IPC Right | 1.2661 | 0.0004 | 0.0015 |
| L_a10p | OPFC Left | 1.0657 | 0.0036 | 0.0101 |
| R_11l | OPFC Right | 1.2423 | 0.0030 | 0.0087 |
| R_a10p | OPFC Right | 1.2444 | 0.0004 | 0.0014 |
| L_FEF | PMC Left | 1.3780 | 0.0002 | 0.0009 |
| L_6a | PMC Left | 2.0648 | 0.0000 | 0.0000 |
| R_6a | PMC Right | 1.8844 | 0.0000 | 0.0000 |
| L_7Pm | SPC Left | 2.2952 | 0.0000 | 0.0000 |
| L_7Am | SPC Left | 2.8365 | 0.0000 | 0.0000 |
| L_7PL | SPC Left | 1.8294 | 0.0000 | 0.0002 |
| L_AIP | SPC Left | 0.9877 | 0.0063 | 0.0158 |
| R_7PL | SPC Right | 3.0937 | 0.0000 | 0.0000 |
| R_AIP | SPC Right | 1.3851 | 0.0002 | 0.0007 |
| R_7Pm | SPC Right | 3.2465 | 0.0000 | 0.0000 |
| R_MIP | SPC Right | 1.2174 | 0.0005 | 0.0018 |
| R_LIPd | SPC Right | 1.5559 | 0.0004 | 0.0015 |
| R_7Am | SPC Right | 3.5112 | 0.0000 | 0.0000 |
| L_44 | IFC Left | -0.8328 | 0.0131 | 0.0321 |
| R_44 | IFC Right | -1.1148 | 0.0003 | 0.0014 |
| R_55b | PMC Right | -0.8749 | 0.0154 | 0.0366 |
| L_Caudate | Caudate Left | -2.5725 | 0.0000 | 0.0000 |
| R_Caudate | Caudate Right | -2.5022 | 0.0000 | 0.0000 |
| L_Putamen | Putamen Left | -1.6060 | 0.0000 | 0.0000 |
| R_Putamen | Putamen Right | -1.3175 | 0.0000 | 0.0001 |
| R_Thalamus | Thalamus Right | -2.0584 | 0.0000 | 0.0000 |
| L_Thalamus | Thalamus Left | -1.4466 | 0.0000 | 0.0000 |

**Supplementary Table 8. Statistical results for participation coefficient (PC) at the 76-node scale, corresponding to the significant group differences shown in Figure 4E-F and underlying the summary pattern shown in Figure 6D.** Only significant results are listed. Rows are ordered by estimate sign (positive first and then negative), followed by alphabetical order of anatomical region within each group. Columns are defined as in Supplementary Table 2. The Estimate column reflects the group mean difference (mean[HC] – mean[ALL]), with positive values indicating decreased PC in ALL survivors and negative values indicating increased PC in ALL survivors. All values are rounded to four decimal places.

| **Node** | **Anatomical Region** | **Estimate** | **Raw P-value** | **FDR P-value** |
| --- | --- | --- | --- | --- |
| R_8C | DLPC Right | 0.0188 | 0.0259 | 0.0438 |
| R_p9-46v | DLPC Right | 0.0255 | 0.0151 | 0.0273 |
| L_44 | IFC Left | 0.0279 | 0.0032 | 0.0067 |
| R_p47r | IFC Right | 0.0177 | 0.0078 | 0.0157 |
| R_44 | IFC Right | 0.0606 | 0.0000 | 0.0000 |
| L_FOP4 | IFOC Left | 0.0217 | 0.0202 | 0.0349 |
| R_FOP5 | IFOC Right | 0.0273 | 0.0099 | 0.0192 |
| R_FOP4 | IFOC Right | 0.0453 | 0.0000 | 0.0000 |
| L_55b | PMC Left | 0.0349 | 0.0001 | 0.0002 |
| R_6r | PMC Right | 0.0308 | 0.0001 | 0.0003 |
| R_55b | PMC Right | 0.0199 | 0.0128 | 0.0244 |
| L_Caudate | Caudate Left | 0. 1910 | 0.0000 | 0.0000 |
| R_Caudate | Caudate Right | 0. 1848 | 0.0000 | 0.0000 |
| L_Putamen | Putamen Left | 0.0637 | 0.0000 | 0.0000 |
| R_Putamen | Putamen Right | 0.0829 | 0.0000 | 0.0000 |
| L_Thalamus | Thalamus Left | 0.0863 | 0.0000 | 0.0000 |
| R_Thalamus | Thalamus Right | 0.0931 | 0.0000 | 0.0000 |
| L_8BM | ACMPC Left | -0.0358 | 0.0023 | 0.0053 |
| L_d32 | ACMPC Left | -0.0754 | 0.0000 | 0.0000 |
| R_d32 | ACMPC Right | -0.0791 | 0.0000 | 0.0000 |
| L_46 | DLPC Left | -0.0233 | 0.0057 | 0.0116 |
| L_9-46d | DLPC Left | -0.0457 | 0.0000 | 0.0000 |
| R_s6-8 | DLPC Right | -0.0261 | 0.0028 | 0.0062 |
| R_9-46d | DLPC Right | -0.0219 | 0.0185 | 0.0327 |
| R_46 | DLPC Right | -0.0302 | 0.0024 | 0.0054 |
| L_p47r | IFC Left | -0.0241 | 0.0014 | 0.0034 |
| L_IFJp | IFC Left | -0.0330 | 0.0002 | 0.0006 |
| R_IFJp | IFC Right | -0.0298 | 0.0005 | 0.0012 |
| L_AVI | IFOC Left | -0.0225 | 0.0017 | 0.0039 |
| L_IP1 | IPC Left | -0.0218 | 0.0006 | 0.0016 |
| L_IP2 | IPC Left | -0.0433 | 0.0000 | 0.0000 |
| R_IP2 | IPC Right | -0.0315 | 0.0000 | 0.0000 |
| R_IP1 | IPC Right | -0.0285 | 0.0001 | 0.0003 |
| L_a10p | OPFC Left | -0.0184 | 0.0007 | 0.0018 |
| L_POS2 | PCC Left | -0.0391 | 0.0000 | 0.0000 |
| R_7m | PCC Right | -0.0164 | 0.0141 | 0.0261 |
| R_POS2 | PCC Right | -0.0338 | 0.0000 | 0.0000 |
| L_6a | PMC Left | -0.1146 | 0.0000 | 0.0000 |
| R_6a | PMC Right | -0.1006 | 0.0000 | 0.0000 |
| L_MIP | SPC Left | -0.0495 | 0.0000 | 0.0000 |
| L_LIPd | SPC Left | -0.0515 | 0.0000 | 0.0000 |
| L_AIP | SPC Left | -0.0720 | 0.0000 | 0.0000 |
| R_MIP | SPC Right | -0.0376 | 0.0000 | 0.0000 |
| R_LIPd | SPC Right | -0.0446 | 0.0000 | 0.0000 |
| R_AIP | SPC Right | -0.0674 | 0.0000 | 0.0000 |

**Supplementary Table 9. Statistical results for clustering coefficient (CC) at the 24-node scale, corresponding to the significant group differences shown in Figure 5A-B and underlying the summary pattern shown in Figure 6A.** Only significant results are listed. Rows are ordered by estimate sign (positive first and then negative), followed by alphabetical order of anatomical region within each group. Columns are defined as in Supplementary Table 2. The Estimate column reflects the group mean difference (mean[HC] – mean[ALL]), with positive values indicating decreased CC in ALL survivors and negative values indicating increased CC in ALL survivors. All values are rounded to four decimal places.

| **Node** | **Estimate** | **Raw P-value** | **FDR P-value** |
| --- | --- | --- | --- |
| DLPC Left | 0.0656 | 0.0000 | 0.0000 |
| DLPC Right | 0.0796 | 0.0000 | 0.0000 |
| IFC Left | 0.0496 | 0.0000 | 0.0000 |
| IFC Right | 0.0228 | 0.0220 | 0.0440 |
| PMC Left | 0.1405 | 0.0000 | 0.0000 |
| PMC Right | 0.1214 | 0.0000 | 0.0000 |
| SPC Right | 0.0559 | 0.0050 | 0.0108 |
| IFOC Right | -0.0319 | 0.0025 | 0.0059 |
| Caudate Left | -0.0678 | 0.0000 | 0.0000 |
| Caudate Right | -0.0452 | 0.0000 | 0.0000 |
| Putamen Right | -0.0387 | 0.0004 | 0.0010 |
| Thalamus Left | -0.0683 | 0.0000 | 0.0000 |

**Supplementary Table 10. Statistical results for eigenvector centrality (EC) at the 24-node scale, corresponding to the significant group differences shown in Figure 5C-D and underlying the summary pattern shown in Figure 6B.** Only significant results are listed. Rows are ordered by estimate sign (positive first and then negative), followed by alphabetical order of anatomical region within each group. Columns are defined as in Supplementary Table 2. The Estimate column reflects the group mean difference (mean[HC] – mean[ALL]), with positive values indicating decreased EC in ALL survivors and negative values indicating increased EC in ALL survivors. All values are rounded to four decimal places.

| **Node** | **Estimate** | **Raw P-value** | **FDR P-value** |
| --- | --- | --- | --- |
| IPC Right | 0.0110 | 0.0156 | 0.0220 |
| IFOC Right | 0.0109 | 0.0039 | 0.0067 |
| PCC Left | 0.0070 | 0.0242 | 0.0323 |
| PCC Right | 0.0107 | 0.0031 | 0.0057 |
| Caudate Left | 0.0317 | 0.0000 | 0.0000 |
| Caudate Right | 0.0264 | 0.0000 | 0.0000 |
| Putamen Left | 0.0084 | 0.0063 | 0.0094 |
| Putamen Right | 0.0239 | 0.0000 | 0.0000 |
| Thalamus Left | 0.0301 | 0.0000 | 0.0000 |
| Thalamus Right | 0.0176 | 0.0000 | 0.0001 |
| ACMPC Left | -0.0112 | 0.0024 | 0.0048 |
| ACMPC Right | -0.0106 | 0.0044 | 0.0070 |
| DLPC Left | -0.0283 | 0.0000 | 0.0000 |
| DLPC Right | -0.0281 | 0.0000 | 0.0000 |
| IFC Left | -0.0155 | 0.0000 | 0.0001 |
| OPFC Right | -0.0144 | 0.0015 | 0.0033 |
| PMC Left | -0.0477 | 0.0000 | 0.0000 |
| PMC Right | -0.0255 | 0.0000 | 0.0000 |

**Supplementary Table 11. Statistical results for local assortativity (LA) at the 24-node scale, corresponding to the significant group differences shown in Figure 5E and underlying the summary pattern shown in Figure 6C.** Only significant results are listed. Rows are ordered by estimate sign (positive first and then negative), followed by alphabetical order of anatomical region within each group. Columns are defined as in Supplementary Table 2. The Estimate column reflects the group mean difference (mean[HC] – mean[ALL]), with positive values indicating decreased LA in ALL survivors. All values are rounded to four decimal places.

| **Node** | **Estimate** | **Raw P-value** | **FDR P-value** |
| --- | --- | --- | --- |
| ACMPC Left | 0.5405 | 0.0018 | 0.0047 |
| ACMPC Right | 0.4755 | 0.0030 | 0.0060 |
| DLPC Left | 0.3323 | 0.0273 | 0.0437 |
| DLPC Right | 0.4260 | 0.0042 | 0.0078 |
| IFC Left | 0.5603 | 0.0012 | 0.0041 |
| IFC Right | 0.5881 | 0.0007 | 0.0028 |
| IPC Right | 0.5417 | 0.0019 | 0.0047 |
| OPFC Left | 0.3842 | 0.0191 | 0.0328 |
| OPFC Right | 0.7121 | 0.0000 | 0.0001 |
| PCC Left | 0.8798 | 0.0000 | 0.0000 |
| PCC Right | 0.7559 | 0.0001 | 0.0006 |
| PMC Left | 0.5133 | 0.0018 | 0.0047 |
| PMC Right | 0.5225 | 0.0023 | 0.0050 |
| SPC Left | 0.8694 | 0.0000 | 0.0000 |
| SPC Right | 1.0753 | 0.0000 | 0.0000 |
| Putamen Right | 0.3748 | 0.0297 | 0.0446 |

**Supplementary Table 12. Statistical results for participation coefficient (PC) at the 24-node scale, corresponding to the significant group differences shown in Figure 5F-G and underlying the summary pattern shown in Figure 6D.** Only significant results are listed. Rows are ordered by estimate sign (positive first and then negative), followed by alphabetical order of anatomical region within each group. Columns are defined as in Supplementary Table 2. The Estimate column reflects the group mean difference (mean[HC] – mean[ALL]), with positive values indicating decreased PC in ALL survivors and negative values indicating increased PC in ALL survivors. All values are rounded to four decimal places.

| **Node** | **Estimate** | **Raw P-value** | **FDR P-value** |
| --- | --- | --- | --- |
| ACMPC Left | 0.0412 | 0.0034 | 0.0102 |
| Caudate Left | 0.1274 | 0.0000 | 0.0000 |
| Caudate Right | 0.0675 | 0.0004 | 0.0017 |
| Thalamus Left | 0.1081 | 0.0000 | 0.0000 |
| Thalamus Right | 0.0550 | 0.0009 | 0.0032 |
| DLPC Right | -0.0769 | 0.0000 | 0.0001 |
| PMC Left | -0.0793 | 0.0000 | 0.0000 |
| PMC Right | -0.0596 | 0.0002 | 0.0010 |

**Supplementary Figures**


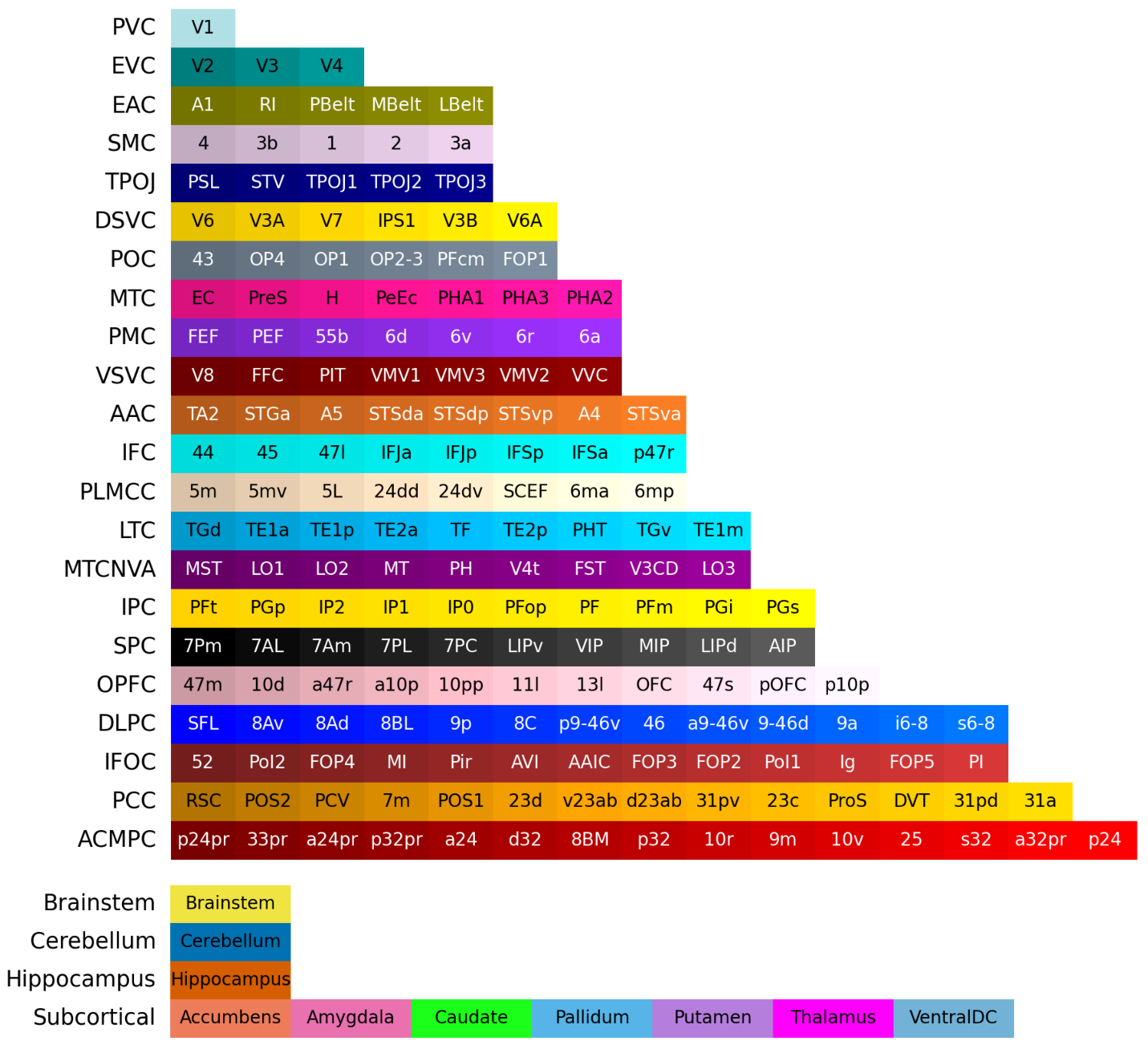


**Supplementary Figure 1. Labels for whole-brain cortical and subcortical parcels defined by the Human Connectome Project Multimodal Parcellation (HCP-MMP1.0), grouped by major anatomical divisions.** This figure provides a reference for parcel names and color coding corresponding to the whole-brain parcel visualizations shown in Figure 1, which are used to define network nodes and interpret connectivity analyses throughout the study. Abbreviations: ACMPC – anterior cingulate medial prefrontal cortex, AAC – auditory association cortex, DLPC – dorsolateral prefrontal cortex, DSVC – dorsal stream visual cortex, EAC – early auditory cortex, EVC – early visual cortex, IFC – inferior frontal cortex, IFOC – insular and frontal opercular cortex, IPC – inferior parietal cortex, LTC – lateral temporal cortex, MTC – medial temporal cortex, MTCNVA – MT+ complex and neighboring visual areas, OPFC – orbitofrontal cortex, PCC – posterior cingulate cortex, PLMCC – paracentral lobular and mid cingulate cortex, PMC – premotor cortex, POC – posterior opercular cortex, PVC – primary visual cortex, SMC – somatosensory and motor cortex, SPC – superior parietal cortex, TPOJ – temporo-parieto-occipital junction, VSVC – ventral stream visual cortex.


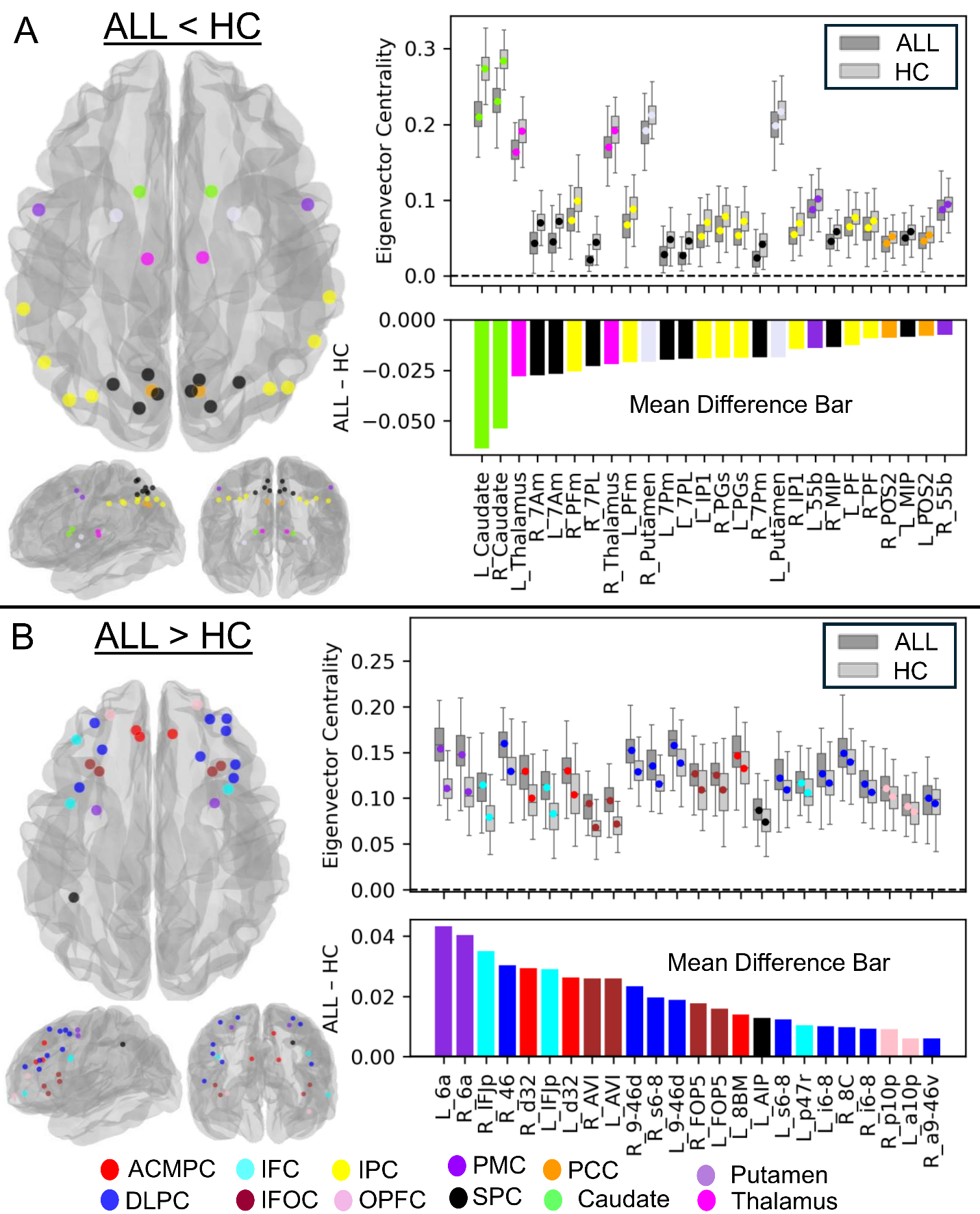


**Supplementary Figure 2. Detailed results for Eigenvector centrality (EC) at the 76-node working memory (WM) structural network scale, corresponding to the EC findings summarized in Figure 4A-B.** (A) Panel shows regions where EC was lower in acute lymphoblastic leukemia (ALL) survivors compared with healthy controls (HC) (ALL < HC), and (B) panel shows regions where EC was higher in ALL survivors (ALL > HC). In each panel, the left subpanel displays glass brain plots highlighting significant cortical and subcortical nodes. The upper right subpanel shows boxplots illustrating the distribution of EC values for ALL and HC groups at each significant node. The lower right subpanel presents bar plots of the mean group difference (ALL − HC) in EC, indicating the direction and magnitude of effects. X-axis labels are shared between boxplots and bar plots. Color coding denotes cortical and subcortical regions as indicated in the legend at the bottom of the figure. Group differences were assessed using multivariable linear regression with group (ALL vs. HC) as the primary predictor and age and sex as covariates, with false discovery rate (FDR) correction applied for multiple comparisons (p < 0.05). The full set of regression estimates, raw p-values, and FDR-corrected p-values for all nodes is provided in Supplementary Table 6. The final sample included N = 70 ALL survivors and N = 70 healthy controls. Abbreviations: ACMPC – anterior cingulate medial prefrontal cortex, DLPC – dorsolateral prefrontal cortex, IFC – inferior frontal cortex, IFOC – insular and frontal opercular cortex, IPC – inferior parietal cortex, OPFC – orbitofrontal cortex, PCC – posterior cingulate cortex, PMC – premotor cortex, SPC – superior parietal cortex.


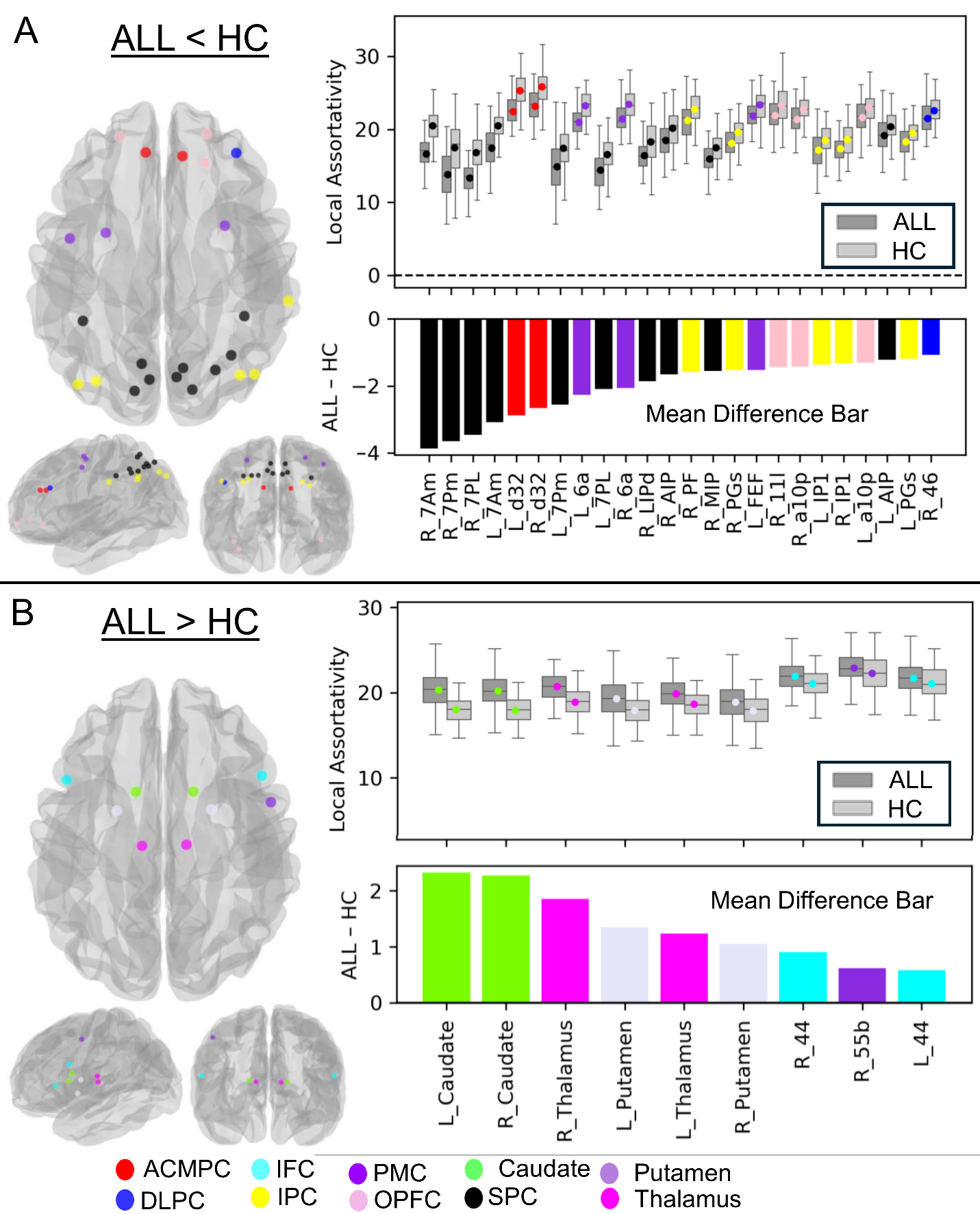


**Supplementary Figure 3.** **Detailed results for local assortativity (LA) at the 76-node working memory (WM) structural network scale, corresponding to the LA findings summarized in Figure 4C-D.** (A) Panel shows regions where LA was lower in acute lymphoblastic leukemia (ALL) survivors compared with healthy controls (HC) (ALL < HC), and (B) panel shows regions where LA was higher in ALL survivors (ALL > HC). In each panel, the left subpanel displays glass brain plots highlighting significant cortical and subcortical nodes. The upper right subpanel shows boxplots illustrating the distribution of LA values for ALL and HC groups at each significant node. The lower right subpanel presents bar plots of the mean group difference (ALL − HC) in LA, indicating the direction and magnitude of effects. X-axis labels are shared between boxplots and bar plots. Color coding denotes cortical and subcortical regions as indicated in the legend at the bottom of the figure. Group differences were assessed using multivariable linear regression with group (ALL vs. HC) as the primary predictor and age and sex as covariates, with false discovery rate (FDR) correction applied for multiple comparisons (p < 0.05). The full set of regression estimates, raw p-values, and FDR-corrected p-values for all nodes is provided in Supplementary Table 7. The final sample included N = 70 ALL survivors and N = 70 healthy controls. Abbreviations: ACMPC – anterior cingulate medial prefrontal cortex, DLPC – dorsolateral prefrontal cortex, IFC – inferior frontal cortex, IPC – inferior parietal cortex, OPFC – orbitofrontal cortex, PMC – premotor cortex, SPC – superior parietal cortex.


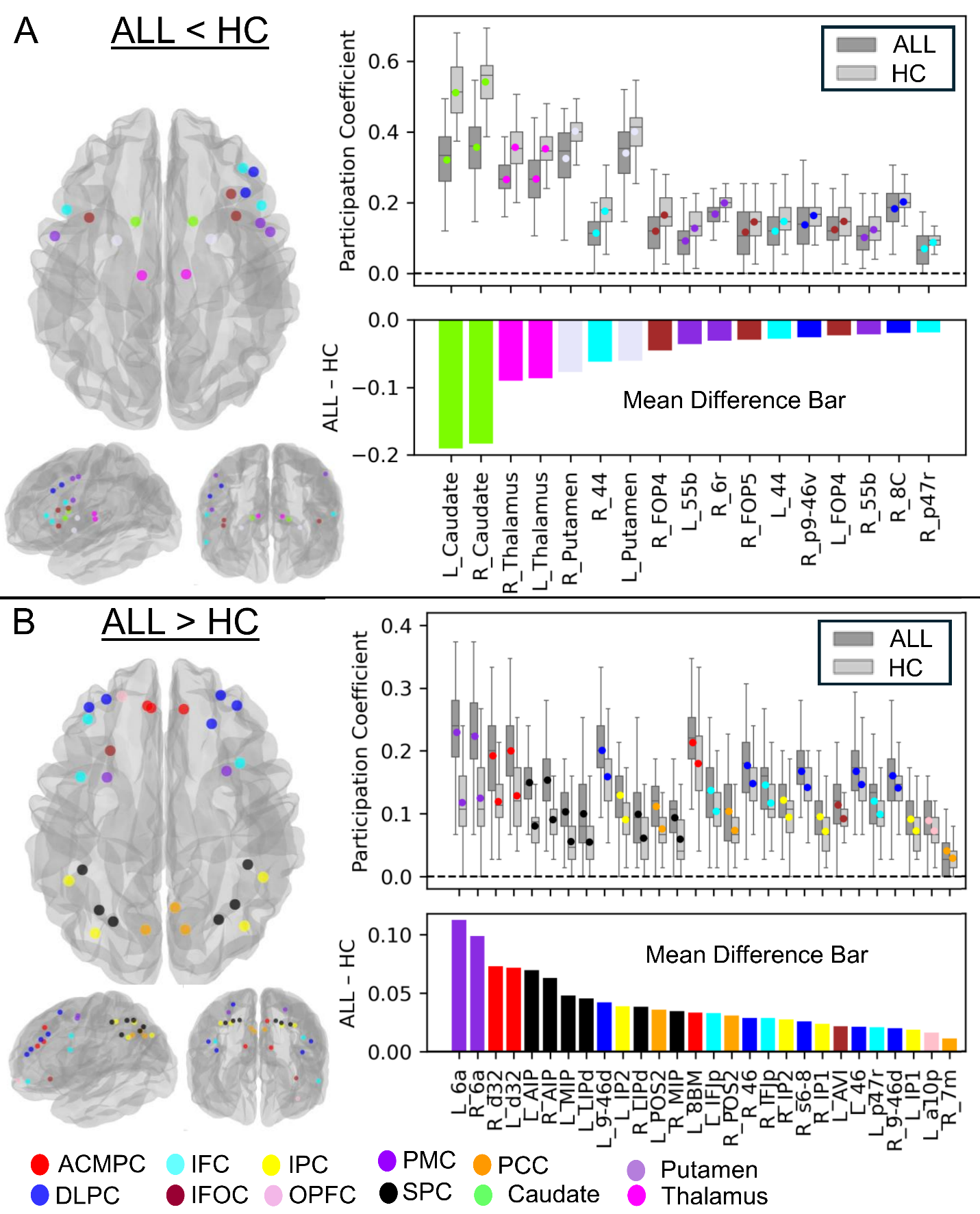


**Supplementary Figure 4.** **Detailed results for participation coefficient (PC) at the 76-node working memory (WM) structural network scale, corresponding to the PC findings summarized in Figure 4E-F.** (A) Panel shows regions where PC was lower in acute lymphoblastic leukemia (ALL) survivors compared with healthy controls (HC) (ALL < HC), and (B) panel shows regions where PC was higher in ALL survivors (ALL > HC). In each panel, the left subpanel displays glass brain plots highlighting significant cortical and subcortical nodes. The upper right subpanel shows boxplots illustrating the distribution of PC values for ALL and HC groups at each significant node. The lower right subpanel presents bar plots of the mean group difference (ALL − HC) in PC, indicating the direction and magnitude of effects. X-axis labels are shared between boxplots and bar plots. Color coding denotes cortical and subcortical regions as indicated in the legend at the bottom of the figure. Group differences were assessed using multivariable linear regression with group (ALL vs. HC) as the primary predictor and age and sex as covariates, with false discovery rate (FDR) correction applied for multiple comparisons (p < 0.05). The full set of regression estimates, raw p-values, and FDR-corrected p-values for all nodes is provided in Supplementary Table 8. The final sample included N = 70 ALL survivors and N = 70 healthy controls. Abbreviations: ACMPC – anterior cingulate medial prefrontal cortex, DLPC – dorsolateral prefrontal cortex, IFC – inferior frontal cortex, IFOC – insular and frontal opercular cortex, IPC – inferior parietal cortex, OPFC – orbitofrontal cortex, PCC – posterior cingulate cortex, PMC – premotor cortex, SPC – superior parietal cortex.


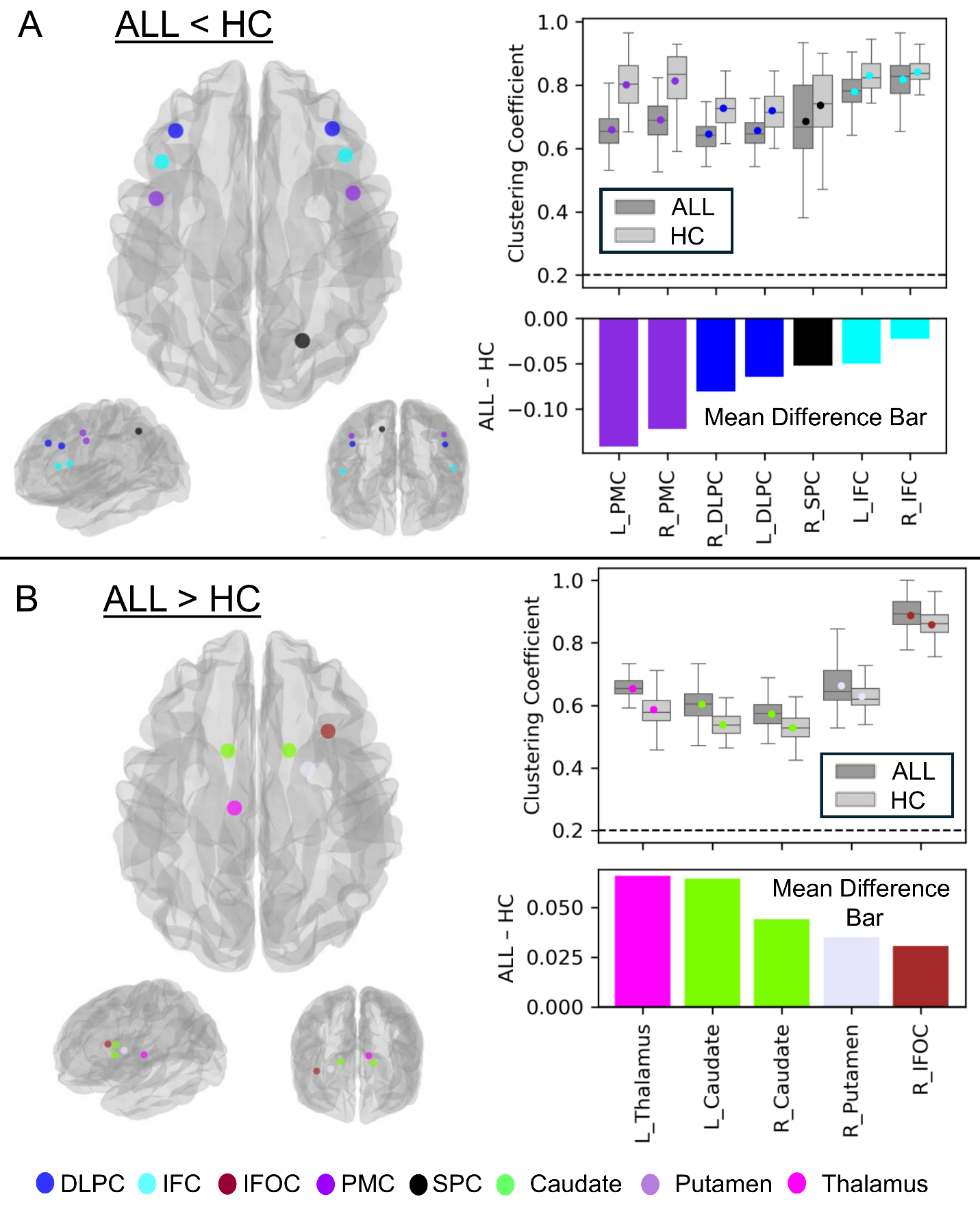


**Supplementary Figure 5.** **Detailed results for clustering coefficient (CC) at the 24-node working memory (WM) structural network scale, corresponding to the CC findings summarized in Figure 5A-B.** (A) Panel shows regions where CC was lower in acute lymphoblastic leukemia (ALL) survivors compared with healthy controls (HC) (ALL < HC), and (B) panel shows regions where CC was higher in ALL survivors (ALL > HC). In each panel, the left subpanel displays glass brain plots highlighting significant cortical and subcortical nodes. The upper right subpanel shows boxplots illustrating the distribution of CC values for ALL and HC groups at each significant node. The lower right subpanel presents bar plots of the mean group difference (ALL − HC) in CC, indicating the direction and magnitude of effects. X-axis labels are shared between boxplots and bar plots. Color coding denotes cortical and subcortical regions as indicated in the legend at the bottom of the figure. Group differences were assessed using multivariable linear regression with group (ALL vs. HC) as the primary predictor and age and sex as covariates, with false discovery rate (FDR) correction applied for multiple comparisons (p < 0.05). The full set of regression estimates, raw p-values, and FDR-corrected p-values for all nodes is provided in Supplementary Table 9. The final sample included N = 70 ALL survivors and N = 70 healthy controls. Abbreviations: DLPC – dorsolateral prefrontal cortex, IFC – inferior frontal cortex, IFOC – insular and frontal opercular cortex, PMC – premotor cortex, SPC – superior parietal cortex.


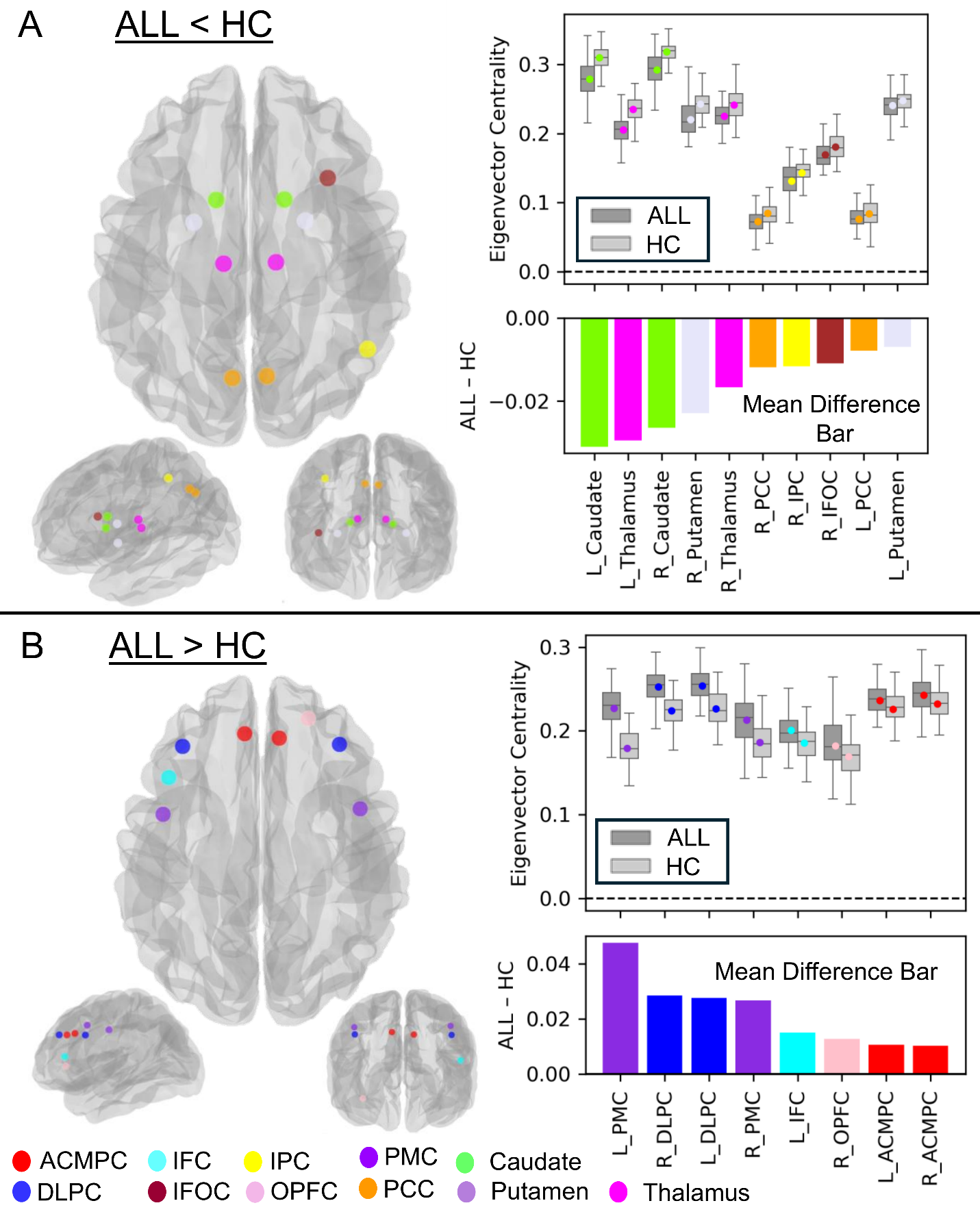


**Supplementary Figure 6.** **Detailed results for Eigenvector centrality (EC) at the 24-node working memory (WM) structural network, corresponding to the EC findings summarized in Figure 5C-D.** (A) Panel shows regions where EC was lower in acute lymphoblastic leukemia (ALL) survivors compared with healthy controls (HC) (ALL < HC), and (B) panel shows regions where EC was higher in ALL survivors (ALL > HC). In each panel, the left subpanel displays glass brain plots highlighting significant cortical and subcortical nodes. The upper right subpanel shows boxplots illustrating the distribution of EC values for ALL and HC groups at each significant node. The lower right subpanel presents bar plots of the mean group difference (ALL − HC) in EC, indicating the direction and magnitude of effects. X-axis labels are shared between boxplots and bar plots. Color coding denotes cortical and subcortical regions as indicated in the legend at the bottom of the figure. Group differences were assessed using multivariable linear regression with group (ALL vs. HC) as the primary predictor and age and sex as covariates, with false discovery rate (FDR) correction applied for multiple comparisons (p < 0.05). The full set of regression estimates, raw p-values, and FDR-corrected p-values for all nodes is provided in Supplementary Table 10. The final sample included N = 70 ALL survivors and N = 70 healthy controls. Abbreviations: ACMPC – anterior cingulate medial prefrontal cortex, DLPC – dorsolateral prefrontal cortex, IFC – inferior frontal cortex, IFOC – insular and frontal opercular cortex, IPC – inferior parietal cortex, OPFC – orbitofrontal cortex, PMC – premotor cortex, PCC – posterior cingulate cortex.


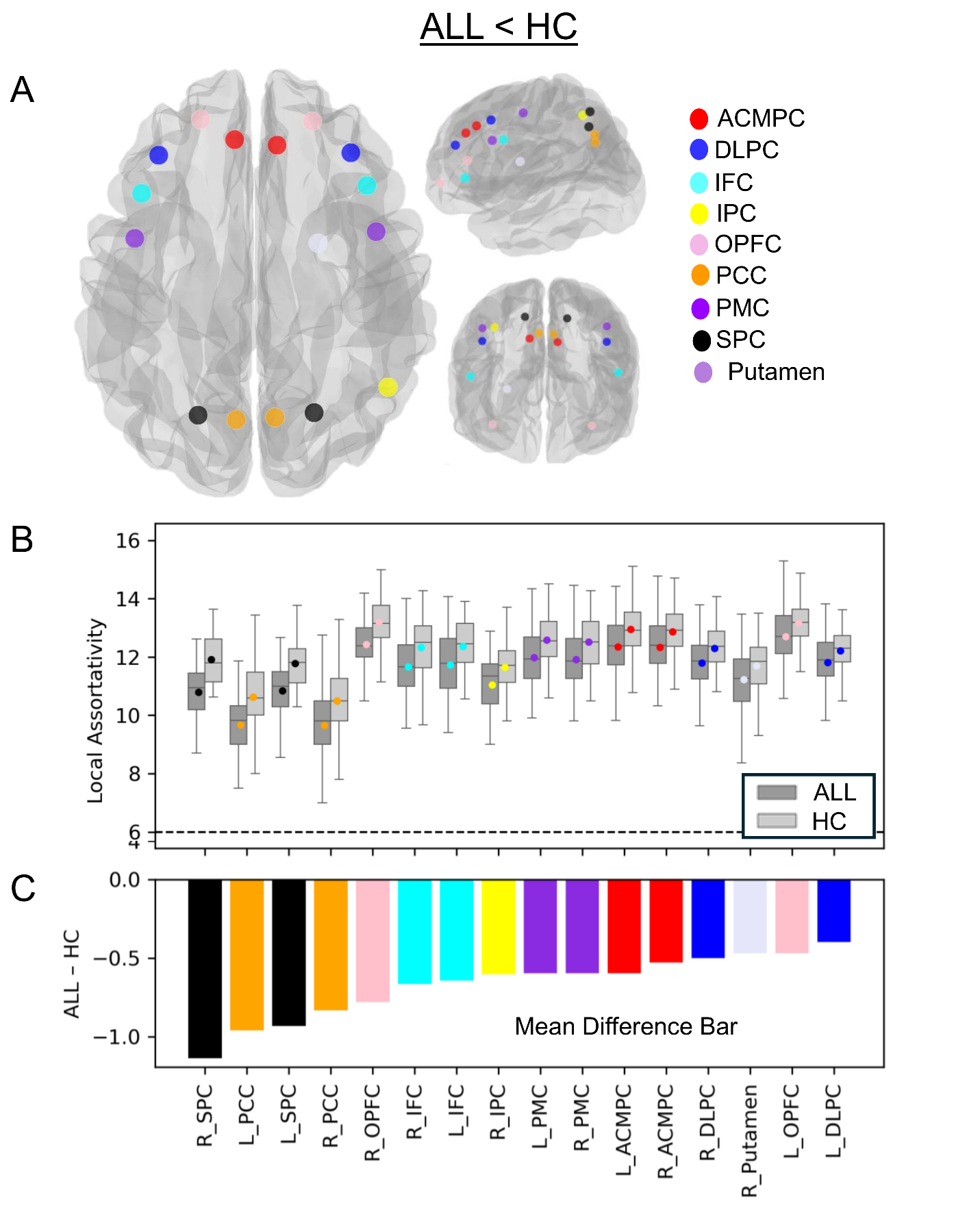
**Supplementary Figure 7.** **Detailed results for local assortativity (LA) at the 24-node working memory (WM) structural network, corresponding to the LA findings summarized in Figure 5E.** Figure shows significant results where LA was reduced in ALL survivors compared to healthy controls (HC) (ALL < HC); no regions exhibited significantly increased LA in the ALL group. (A) Glass brain plots highlight significant nodes, with legend indicating color coded cortical and subcortical regions. (B) Boxplots show groupwise distributions of LA values for each significant node in the ALL and HC groups. (C) Bar plots display the mean difference (ALL − HC) in LA, illustrating the direction and magnitude of group effects. X-axis labels are shared between boxplots and bar plots. Group differences were assessed using multivariable linear regression with group (ALL vs. HC) as the primary predictor and age and sex as covariates, with false discovery rate (FDR) correction applied for multiple comparisons (p < 0.05). The full set of regression estimates, raw p-values, and FDR-corrected p-values for all nodes is provided in Supplementary Table 11. The final sample included N = 70 ALL survivors and N = 70 healthy controls. Abbreviations: ACMPC – anterior cingulate medial prefrontal cortex, DLPC – dorsolateral prefrontal cortex, IFC – inferior frontal cortex, IPC – inferior parietal cortex, OPFC – orbitofrontal cortex, PCC – posterior cingulate cortex, PMC – premotor cortex, SPC – superior parietal cortex.


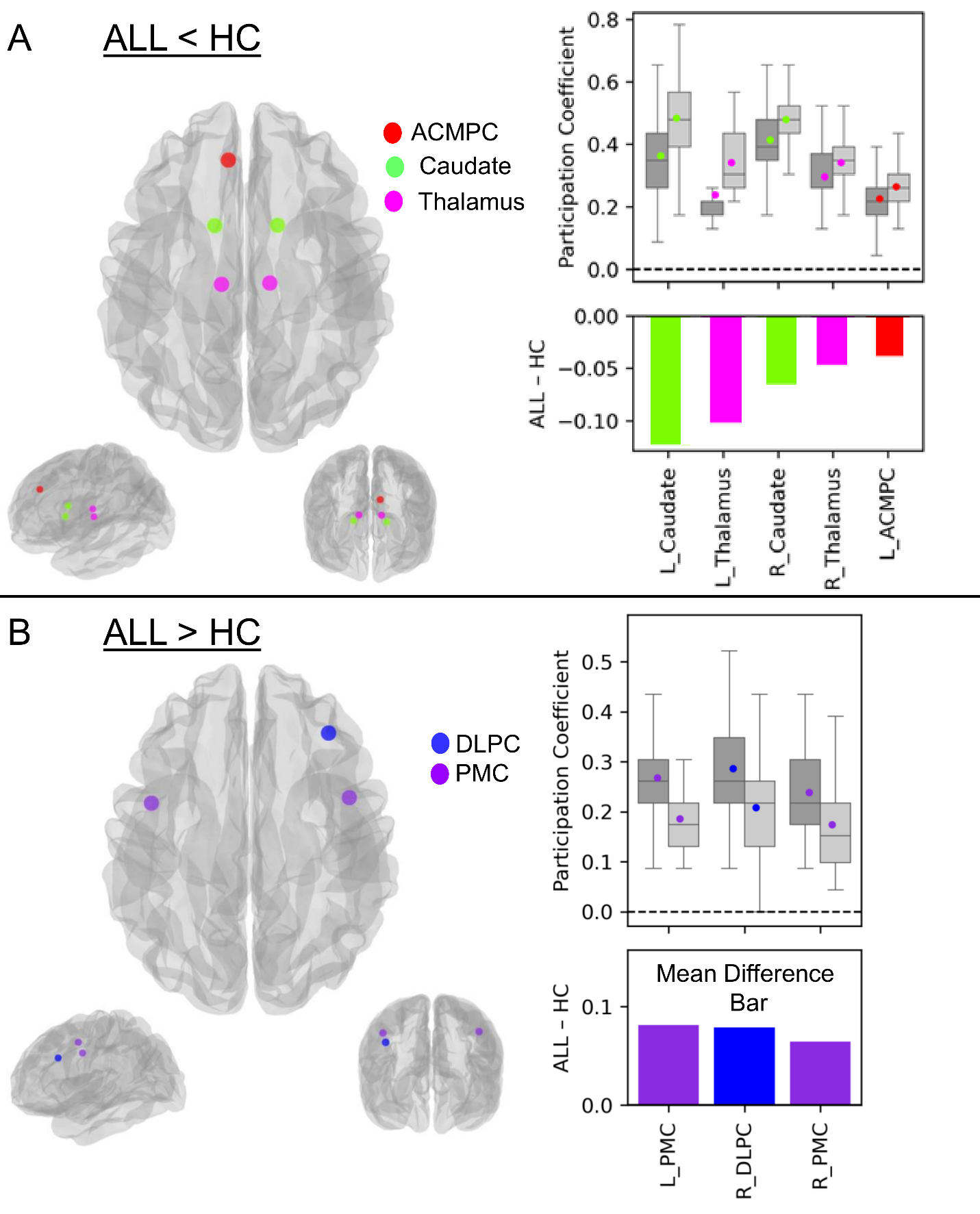


**Supplementary Figure 8.** **Detailed results for participation coefficient (PC) at the 24-node working memory (WM) structural network, corresponding to the PC findings summarized in Figure 5F-G.** (A) Panel shows regions where PC was lower in acute lymphoblastic leukemia (ALL) survivors compared with healthy controls (HC) (ALL < HC), and (B) panel shows regions where PC was higher in ALL survivors (ALL > HC). In each panel, the left subpanel displays glass brain plots highlighting significant cortical and subcortical nodes, with legend indicating color coded cortical and subcortical regions. The upper right subpanel shows boxplots illustrating the distribution of PC values for ALL and HC groups at each significant node. The lower right subpanel presents bar plots of the mean group difference (ALL − HC) in PC, indicating the direction and magnitude of effects. X-axis labels are shared between boxplots and bar plots. Group differences were assessed using multivariable linear regression with group (ALL vs. HC) as the primary predictor and age and sex as covariates, with false discovery rate (FDR) correction applied for multiple comparisons (p < 0.05). The full set of regression estimates, raw p-values, and FDR-corrected p-values for all nodes is provided in Supplementary Table 12. The final sample included N = 70 ALL survivors and N = 70 healthy controls. Abbreviations: ACMPC – anterior cingulate medial prefrontal cortex, DLPC – dorsolateral prefrontal cortex, PMC – premotor cortex.

Supplementary references

1. Rubinov M, Sporns O. Complex network measures of brain connectivity: Uses and interpretations. *Neuroimage*. 2010;52(3):1059-1069. doi:10.1016/j.neuroimage.2009.10.003

2. Hagberg AA, Schult DA, Swart PJ. Exploring Network Structure, Dynamics, and Function using NetworkX. In: *Proceedings of the 7th Python in Science Conference*. 2008. doi:10.25080/tcwv9851

3. Lohmann G, Margulies DS, Horstmann A, et al. Eigenvector centrality mapping for analyzing connectivity patterns in fMRI data of the human brain. *PLoS One*. 2010;5(4). doi:10.1371/journal.pone.0010232

4. Newman M. *Networks: An Introduction*. Oxford University Press; 2010. doi:10.1093/acprof:oso/9780199206650.001.0001

5. Newman MEJ. Assortative Mixing in Networks. *Phys Rev Lett*. 2002;89(20). doi:10.1103/PhysRevLett.89.208701

6. Watts DJ, Strogatz SH. Collective dynamics of ’small-world9 networks. *Nature*. 1998;393(6684). doi:10.1038/30918

7. Guimerà R, Amaral LAN. Functional cartography of complex metabolic networks. *Nature*. 2005;433(7028). doi:10.1038/nature03288

8. Bullmore E, Sporns O. Complex brain networks: graph theoretical analysis of structural and functional systems. *Nat Rev Neurosci*. 2009;10(3):186-198. doi:10.1038/nrn2575

9. Stam CJ, Jones BF, Nolte G, Breakspear M, Scheltens P. Small-world networks and functional connectivity in Alzheimer’s disease. *Cerebral Cortex*. 2007;17(1). doi:10.1093/cercor/bhj127

10. Zuo XN, Ehmke R, Mennes M, et al. Network centrality in the human functional connectome. *Cerebral Cortex*. 2012;22(8). doi:10.1093/cercor/bhr269

11. Crossley NA, Mechelli A, Scott J, et al. The hubs of the human connectome are generally implicated in the anatomy of brain disorders. *Brain*. 2014;137(8). doi:10.1093/brain/awu132

12. Alexander-Bloch A, Lambiotte R, Roberts B, Giedd J, Gogtay N, Bullmore E. The discovery of population differences in network community structure: New methods and applications to brain functional networks in schizophrenia. *Neuroimage*. 2012;59(4). doi:10.1016/j.neuroimage.2011.11.035

13. Bertolero MA, Yeo BTT, D’Esposito M. The diverse club. *Nat Commun*. 2017;8(1). doi:10.1038/s41467-017-01189-w

14. van den Heuvel MP, Sporns O. Network hubs in the human brain. *Trends Cogn Sci*. 2013;17(12):683-696. doi:10.1016/j.tics.2013.09.012
