# Supplementary material for: Multiscale connectivity framework for working memory network in paediatric acute lymphoblastic leukaemia survivors
Source: Brain Commun. 2026 Apr 17;8(2):fcag137. doi: 10.1093/braincomms/fcag137 (PMC13126661; doi:10.1093/braincomms/fcag137)
Supplement: fcag137_Supplementary_Data [file Original_submission_decision_letter.pdf › Original_submission_decision_letter.pdf]

From: brcoms.editorialoffice@paeditorial.co.uk
To:
CC:
BCC:
Subject: Brain Communications - Decision on Manuscript ID BRAINCOM-2025-1002
Body: 26-Nov-2025

Dear Dr. Raja,

Thank you for your recent submission BRAINCOM-2025-1002 entitled "Multiscale Connectivity Framework for Working Memory Network in Pediatric Acute Lymphoblastic Leukemia Survivors" to Brain Communications.

Your manuscript has been reviewed, and while the reviewers and editorial team appreciate many aspects of the paper, they raise some concerns that you will need to address in order for the paper to be considered for publication.

If you can address these concerns, I invite you to submit a revised manuscript and a response to each of the reviewers' comments. The reviewer(s)' comments are included at the bottom of this message.

In addition to the reviewers' comments, please also address any comments from our Scientific Editor added below to comply with our requirements for presentation of scientific data at Brain Communications. Please note we will not accept the paper unless you comply with our policies on data analysis and presentation as noted in the comments from the Scientific Editor.

Please format your revised manuscript according to our Instructions to Authors
https://academic.oup.com/braincomms/pages/General_Instructions
A summary of key points is attached to help with this. Please note that there can be substantial delays to publication if you don't resubmit in line with our requirements.

To revise your manuscript, click the link below or log into https://mc.manuscriptcentral.com/braincom and enter your Author Centre, where you will find your manuscript title listed under "Manuscripts with Decisions." Under "Actions," click on "Create a Revision." Your manuscript number will be appended to denote a revision.

*** PLEASE NOTE: This is a two-step process. After clicking on the link, you will be directed to a webpage to confirm. ***

https://mc.manuscriptcentral.com/braincom?URL_MASK=d20771e6f09b46c3a4c6121e9eebed3c

Please upload a revised manuscript with changes tracked with track changes or in coloured text.

When submitting your revised manuscript, you will be asked to respond to the reviewer(s) comments. Please be as specific as possible in your response to all of the comments.

The editorial team at Brain Communications aims to increase transparency in scientific publishing and to demonstrate the value that peer review brings to the scientific process. Please therefore consider whether you are willing to have reviewer(s)' comments on your paper and your response published as supplementary data with the paper if it is accepted. This is entirely optional and will only be done if both the authors and reviewers agree. While submitting your revision there will be an opportunity to review or update the answer you gave previously, if necessary.

IMPORTANT: Your original files are available to you when you upload your revised manuscript. Please delete any redundant files before completing the submission.

Because we are trying to facilitate timely publication of manuscripts submitted to Brain Communications, we would appreciate your revised manuscript as soon as possible. If it is not possible for you to resubmit within 3 months, the option to submit a revision will expire, and we would then consider your revised paper as a new submission. However, if you need a little more time, please get in touch and we can extend this deadline for you.

Please note that Brain Communications is a fully open access journal, and if your article is accepted for publication, an open access charge will be payable (this does not apply to letters and scientific commentaries). Details can be found in our Instructions for Authors: https://academic.oup.com/braincomms/pages/General_Instructions

Once again, thank you for submitting your manuscript to Brain Communications and I look forward to receiving your revision.

Best wishes

Professor Tara Spires-Jones
Founding Editor, Brain Communications

Reviewer(s)' Comments to Author:

Reviewer: 1

Comments to the Author
This study presents a novel multiscale structural connectivity framework to investigate working memory (WM) networks in survivors of childhood acute lymphoblastic leukemia (ALL), a population known to experience WM impairments as a late effect of treatment. Using diffusion MRI and graph theoretical analysis, the authors compared WM-specific network topology between 70 ALL survivors and matched healthy controls across fine and coarse spatial scales. The findings reveal consistent topological differences, characterized by increased subcortical clustering and segregation alongside lowered integration. This is an interesting study with a

relatively large sample size for a clinical population, and the study presents a robust structural connectivity framework.
However, there are some concerns that warrant attention.
• First, the definition of the working memory network relies on reverse inference from a single adult fMRI study (Glasser et al., 2016), which used an n-back task to localize WM-related regions. This approach risks conflating WM-specific activation with broader cognitive functions, as many of the implicated regions are involved in multiple domains. Moreover, the Glasser atlas is derived from adult data and does not incorporate developmental samples such as the HCP-D sample, limiting its applicability to pediatric populations where working memory circuitry and activation patterns change with age. The specificity of the working memory network should be validated against behavioral measures or meta-analytic functional data from paediatric cohorts (e.g., via NeuroSynth or NeuroVault). If working memory behavioral data were collected in this sample, relating network topology to performance would strengthen the authors claims of functional relevance. The authors note this as a limitation, so this might mean no such cognitive data exists. Without such validation, any claims that there are examining WM networks are highly dubious.
• While the diffusion MRI processing is rigorous, the matching procedure for age and biological sex lacks detail. It remains unclear whether matching was one-to-one or based on broader criteria, and the marginal age difference between groups. Including age and sex as covariates post-matching introduces redundancy, as matching is typically intended to mitigate the need for such statistical controls.
• The rationale for using both 76-node and 24-node scales is not clearly explained.
• I found the Discussion section difficult to follow at times.
• The literature shows that differences in network topology are evident across the whole brain in pediatric cancer patients. What is the evidence that differences the authors observed are specific working memory networks and not simply reflecting a broader disruption of brain connectivity?
• The physiological meaning of these topological differences between ALL and HC is not discussed clearly; The authors suggest links between graph metrics and cellular processes — i.e., "effects that appeared only at the 76-node scale suggest highly focal or subregional changes such as micro-circuit pruning, early demyelination, or small-scale compensatory sprouting that are too spatially constrained to sway the coarser parcellation"—
such interpretation is speculative, especially given that diffusion MRI (dMRI) lacks the resolution to detect such cellular level differences.

Comments from the Scientific Editorial team:

-Please add an Abbreviated Summary (up to 50 words) of your paper that captures the main purpose and conclusions of the work. The summary should state the main results written in the third person perspective ('[1st author surname] et al. report that ...') and conclusion. This summary will be used in the contents list online and should not contain any abbreviations other than those on the accepted abbreviations list and accepted gene/protein names, making it accessible to the non-specialist. Please upload it as a Word document. Allowed abbreviations are: AIDS; ANOVA; AMPA; ATP; A,T,C,G; CNS; CSF; CT; DNA; ECG; EEG; EMG; GABA; HIV; MRI; NMDA; PET; PCR; RNA.

-Please provide a Graphical Abstract. A graphical abstract is a visual representation of the

central finding or methodology of the paper. It will be displayed in the table of contents. Authors should strive to make the graphical abstract informative, interesting, visually appealing, and straightforward (no caption needed, no abbreviations except gene names). The graphical abstract must not contain any elements for which you do not hold the copyright, such as figures or images from other articles or websites etc. The technical requirements for the graphical abstracts are:
- Size: 120 x 120 mm (300-600dpi) or 3000 x 3000 pixels
- Font: Arial 12-16 pt
- File type: tiff or PDF
- Aspect ratio: 1:1 preferred

-Data availability: Thank you for providing a data availability statement. Please also provide any codes generated and used in this work within the supplementary material or through a repository, whose link has to be made available within the data availability statement.

-Figures 1-4: Please increase the font size of the text in the figures. Please also consider increasing the sizes of the subfigure panels. This is to ensure that the figures are easily viewable when fitted on a typeset journal page.

-Figures 1 and 2: If not already spelt out in the main text, please ensure all abbreviations used in the figure and figure legend are defined within the figure legend so that they may be easily interpreted as stand-alone figures.

-Figures 1, 2A, 2C, 2E, 3, 4 and 5, Supplementary Figures 1-3, 5-7: Please revisit your colour choice in this figure as the green-red combination is not colour-blind friendly.

-Figures 1 and 6: Please use upper case letters (A, B, C, etc.) to label panels/ subfigures in the top left corner (no brackets or full stops) and then in the figure legend describe using the letters what is shown in each panel, unless it is self-explanatory.

-Figures 2, 3, 4, 5 and 6, Supplementary Figures 1-7: For each statistical analysis part of a figure, please report in the figure legend the statistical test used and relevant test statistic values alongside p values (for example F or t values for ANOVAs and t-tests). Also, please provide details of the sample size (N=), and if the number is <10 show the single data points. For individual data points shown, please describe what each data point represents.

-Figure 2: Please ensure that all heatmap scale bars are provided and labelled with titles and their appropriate units.

-Please note our policy on code availability. If any codes were generated for data analysis for this manuscript (e.g. using R, MATLAB, or similar), please make this code available either via an online repository (and provide a link within the data availability statement), or within the manuscript.

-As a reminder at this revision stage, if you plan to edit the current figures and tables, or to make new figures and tables, please ensure that these items fit a typeset journal page (the area available for a figure or table is 170 mm x 210 mm), or are uploaded as supplementary material. Please view your figures at this size and make sure that all lettering and text are large enough to

read.

-Kindly note that if you have used BioRender software to create images in this manuscript, please provide the associated license for each image. If it is used in the graphical abstract, please add the phrase 'created in BioRender' within the image, and the link created after making the figure publicly available on the BioRender website. If it is used in a figure within the paper, please add the phrase 'created in Biorender' within the figure legend, and the link created after making the figure publicly available on the BioRender website.
