# Supplementary material for: Multiscale connectivity framework for working memory network in paediatric acute lymphoblastic leukaemia survivors
Source: Brain Commun. 2026 Apr 17;8(2):fcag137. doi: 10.1093/braincomms/fcag137 (PMC13126661; doi:10.1093/braincomms/fcag137)
Supplement: fcag137_Supplementary_Data [file Original_submission_Response_to_reviewers_comments.pdf › Original_submission_Response_to_reviewers_comments.pdf]

Response to Reviewers

We thank the Editor and Reviewers for their thoughtful and constructive feedback. Below we respond point-by-point. Reviewer comments are italicized, our responses in plain text.

Reviewer 1:

1. *This study presents a novel multiscale structural connectivity framework to investigate working memory (WM) networks in survivors of childhood acute lymphoblastic leukemia (ALL), a population known to experience WM impairments as a late effect of treatment. Using diffusion MRI and graph theoretical analysis, the authors compared WM-specific network topology between 70 ALL survivors and matched healthy controls across fine and coarse spatial scales. The findings reveal consistent topological differences, characterized by increased subcortical clustering and segregation alongside lowered integration. This is an interesting study with a relatively large sample size for a clinical population, and the study presents a robust structural connectivity framework. However, there are some concerns that warrant attention.*

   We thank the reviewer for the clear and thoughtful summary of our work and for recognizing the novelty of our multiscale framework, the strength of our clinical sample, and the consistency of our findings. We also appreciate the positive comments regarding the rigor of our diffusion MRI and graph-theoretical methods. We are grateful for this feedback and have carefully addressed each concern. Below, we provide point-by-point responses and have revised the manuscript accordingly.

2. *First, the definition of the working memory network relies on reverse inference from a single adult fMRI study (Glasser et al., 2016), which used an n-back task to localize WM-related regions. This approach risks conflating WM-specific activation with broader cognitive functions, as many of the implicated regions are involved in multiple domains. Moreover, the Glasser atlas is derived from adult data and does not incorporate developmental samples such as the HCP-D sample, limiting its applicability to pediatric populations where working memory circuitry and activation patterns change with age. The specificity of the working memory network should be validated against behavioral measures or meta-analytic functional*

data from pediatric cohorts (e.g., via NeuroSynth or NeuroVault). If working memory behavioral data were collected in this sample, relating network topology to performance would strengthen the authors claims of functional relevance. The authors note this as a limitation, so this might mean no such cognitive data exists. Without such validation, any claims that there are examining WM networks are highly dubious.

We thank the reviewer for raising this important feedback. This comment raises three related concerns regarding (i) the specificity of our WM definition, (ii) the applicability of the HCP-MMP1 atlas to pediatric populations, and (iii) the absence of behavioral WM data. We respond to each point below.

WM specificity and terminology:

We agree that relying on a single adult fMRI contrast introduces limitations and that regions activated during an n-back task may support broader cognitive control functions beyond WM. We have revised the manuscript to reflect this. Throughout the manuscript, we have replaced the term "WM specific" with "WM associated" to more accurately reflect that the selected regions are activated during WM tasks but may also support broader executive functions.

Applicability of the HCP-MMP1 atlas in pediatric population:

Pediatric-specific multimodal diffusion MRI parcellations remain less established, with substantial developmental variability in cortical boundaries and no broadly accepted multimodal atlas optimized for children.[1,2] However, the HCP-MMP1 parcellation provides multimodal, neuroanatomically precise boundaries that are widely used in both adult and developmental datasets, including large pediatric studies such as ABCD and HCP-D, demonstrating its stability and suitability for mixed-age structural analyses.[3,4] We have expanded the Limitations and future directions subsection to clarify this.

Lack of behavioral WM measures:

As the reviewer noted, WM behavioral data is not available for this cohort. We have revised the Limitations and future directions subsection of Discussion section to explicitly state that direct structure-function associations cannot be assessed in the present dataset. We agree that

future work incorporating harmonized neurocognitive assessments will be essential for linking WM associated network topology to behavioral outcomes.

3. *While the diffusion MRI processing is rigorous, the matching procedure for age and biological sex lacks detail. It remains unclear whether matching was one-to-one or based on broader criteria, and the marginal age difference between groups. Including age and sex as covariates post-matching introduces redundancy, as matching is typically intended to mitigate the need for such statistical controls.*

We thank the reviewer for pointing out the need for additional clarity regarding the age and sex matching procedure. We have revised the manuscript to more explicitly describe how matching was performed and to explain our rationale for including age and sex as covariates in the statistical analyses.

Specifically, healthy controls were selected from the HCP-D dataset using group-level frequency matching on age and biological sex. Matching was based on:

- Age, using ±1-year tolerance bins, and
- Biological sex, ensuring identical male-to-female ratios in both groups.

This approach was designed to align the overall demographic distributions of ALL survivors and healthy controls, rather than to create explicit case–control pairs. From the available 97 ALL survivors and 176 controls, this procedure resulted in a final sample of 70 ALL survivors and 70 healthy controls with comparable age distributions and sex ratios.

Because frequency matching does not guarantee exact balance at the individual level, particularly for continuous variables such as age, a small residual age difference remained between groups ($p = 0.09$). In this context, including age and sex as covariates is standard practice and serves to control residual variability and improve statistical precision, rather than introducing redundancy.[5,6]

4. *The rationale for using both 76-node and 24-node scales is not clearly explained.*

We thank the reviewer for noting the need to clarify our rationale for using both the 76-node and 24-node WM-associated parcellation scales.

To capture the full spatial complexity of WM associated organization, we evaluated the network at two complementary parcellation scales. The 76-node representation preserves the fine-grained anatomical detail of the HCP-MMP1 atlas and enables detection of subtle, subregional variations in WM associated connectivity showing patterns that may be heterogeneous or highly localized and are often diluted when parcels are spatially aggregated. In contrast, the 24-node scale consolidates spatially contiguous and functionally coherent parcels into broader anatomical units, yielding a more interpretable meso-scale summary that reduces analytic complexity and the multiple comparison burden. Prior work has shown that graph theoretical metrics are sensitive to the choice of parcellation granularity, and multiscale analyses can reveal effects that are either consistent across levels or visible only at specific resolutions.[7–9] Accordingly, using both scales allows us to test whether group differences in WM associated topology are focal and region-specific or reflect robust, convergent patterns across hierarchical spatial organization. This multiscale design provides a principled framework for examining the stability, specificity, and interpretive richness of WM associated network alterations in ALL survivors. We have updated the Introduction section with this explanation.

5. *I found the Discussion section difficult to follow at times.*

We thank the reviewer for this helpful feedback. In response, we have substantially revised and reorganized the Discussion to improve clarity, readability, and logical flow with the following revisions:

- Enhanced the introductory paragraph of the Discussion. This includes briefly outlining the three multiscale scenarios, consistent, scale-specific, and heterogeneous patterns up front to guide the reader before the detailed descriptions.
- Refined the overall structure by reorganizing the section into clearer subsections. This includes separating one subsection into multiple parts to better distinguish between the different multiscale scenarios.
- Simplified several complex sentences and improved transitions to make the narrative easier to follow.

- Removed speculative cellular-level language and reframed interpretations in network-topological rather than physiological terms.
- Updated the Limitations section to clarify methodological constraints and improve readability, reflecting related revisions made in response to other comments.

6. *The literature shows that differences in network topology are evident across the whole-brain in pediatric cancer patients. What is the evidence that differences the authors observed are specific working memory networks and not simply reflecting a broader disruption of brain connectivity?*

   We thank the reviewer for raising this important question regarding the specificity of our findings to WM associated regions versus broader whole-brain disruption. We agree that previous studies have reported widespread network alterations in pediatric cancer survivors, and the observed effects may not be exclusive to WM circuitry. We have therefore replaced the term "WM specific" with "WM associated" throughout the manuscript to avoid implying functional exclusivity. Our goal in this study was to examine topological organization within regions activated during WM tasks, rather than to conclude that the alterations occur only within WM pathways.

   In the revised manuscript, we have updated the Limitations section to address issues related to WM specificity. Specifically, we now note that confirming WM specificity would require behavioral data, which were not available for this cohort. Therefore, future work incorporating cognitive measures will be essential to determine whether the observed differences relate uniquely to WM function. We also clarify that our analysis was restricted to WM associated regions and did not include whole-brain comparisons. Because broader whole-brain disruptions may coexist, the Limitations now explicitly highlight this point and emphasize that future whole-brain multiscale analyses will be important for evaluating the relative specificity of WM associated effects.

7. *The physiological meaning of these topological differences between ALL and HC is not discussed clearly; The authors suggest links between graph metrics and cellular processes — i.e., "effects that appeared only at the 76-node scale suggest highly focal or subregional*

changes such as micro-circuit pruning, early demyelination, or small-scale compensatory sprouting that are too spatially constrained to sway the coarser parcellation"—such interpretation is speculative, especially given that diffusion MRI (dMRI) lacks the resolution to detect such cellular level differences.

We thank the reviewer for this important observation. We agree that although diffusion MRI is sensitive to microstructural tissue properties such as axonal organization and myelination, it does not allow direct inference about specific cellular processes and that interpretations referring to micro-circuit pruning, demyelination, or sprouting are speculative. Our intention was to convey that some effects appear highly localized at fine spatial scales, but we agree that the previous language implied biological mechanisms that are not directly measurable with diffusion MRI. In response, we have removed cellular-level speculation and modified the corresponding line in Discussion section as below.

"The first scenario involves effects that appeared only at the 76-node scale, suggesting highly focal or subregional topological differences that are too spatially localized to influence the coarser parcellation level."

Comments from the Scientific Editorial team:

1. *Please add an Abbreviated Summary (up to 50 words) of your paper that captures the main purpose and conclusions of the work. The summary should state the main results written in the third person perspective ('[1st author surname] et al. report that …') and conclusion. This summary will be used in the contents list online and should not contain any abbreviations other than those on the accepted abbreviations list and accepted gene/protein names, making it accessible to the non-specialist. Please upload it as a Word document. Allowed abbreviations are: AIDS; ANOVA; AMPA; ATP; A, T, C,G; CNS; CSF; CT; DNA; ECG; EEG; EMG; GABA; HIV; MRI; NMDA; PET; PCR; RNA.*

   We have prepared the required Abbreviated Summary and have uploaded it as a separate Word document through the journal's submission system, in accordance with the guidelines.

2. *Please provide a Graphical Abstract. A graphical abstract is a visual representation of the central finding or methodology of the paper. It will be displayed in the table of contents. Authors should strive to make the graphical abstract informative, interesting, visually appealing, and straightforward (no caption needed, no abbreviations except gene names). The graphical abstract must not contain any elements for which you do not hold copyright, such as figures or images from other articles or websites etc. The technical requirements for the graphical abstracts are: - Size: 120 x 120 mm (300-600dpi) or 3000 x 3000 pixels - Font: Arial 12-16 pt - File type: tiff or PDF - Aspect ratio: 1:1 preferred*

   We have prepared the required Graphical Abstract and have uploaded it as a separate document through the journal's submission system, in accordance with the guidelines.

3. *Data availability: Thank you for providing a data availability statement. Please also provide any codes generated and used in this work within the supplementary material or through a repository, whose link has to be made available within the data availability statement.*

We have revised the Data Availability statement to include additional details on the tools and software used at each stage of the processing pipeline. Custom analysis code is now available in a public Github repository. The Data Availability statement has been updated with the GitHub link.

4. *Figures 1-4: Please increase the font size of the text in the figures. Please also consider increasing the sizes of the subfigure panels. This is to ensure that the figures are easily viewable when fitted on a typeset journal page.*

   Font sizes and panel dimensions have been increased throughout Figures 1–4 to ensure readability at the journal's typeset size (170 mm × 210 mm). All text remains clear when viewed at the required physical dimensions.

5. *Figures 1 and 2: If not already spelt out in the main text, please ensure all abbreviations used in the figure and figure legend are defined within the figure legend so that they may be easily interpreted as stand-alone figures.*

   All abbreviations appearing in Figures 1 and 2 have now been fully defined within each figure legend.

6. *Figures 1, 2A, 2C, 2E, 3, 4 and 5, Supplementary Figures 1-3, 5-7: Please revisit your color choice in this figure as the green-red combination is not colour-blind friendly.*

   All figures listed have been recolored. Red-green combinations have been removed by replacing green with cyan.

7. *Figures 1 and 6: Please use upper case letters (A, B, C, etc.) to label panels/ subfigures in the top left corner (no brackets or full stops) and then in the figure legend describe using the letters what is shown in each panel, unless it is self-explanatory.*

Panel labels have been updated to uppercase letters placed in the top-left corner of each subpanel. The figure legends modified to describe each panel using these labels.

8. *Figures 2, 3, 4, 5 and 6, Supplementary Figures 1-7: For each statistical analysis part of a figure, please report in the figure legend the statistical test used and relevant test statistic values alongside p values (for example F or t values for ANOVAs and t-tests). Also, please provide details of the sample size (N=), and if the number is <10 show the single data points. For individual data points shown, please describe what each data point represents.*

   We thank the editors for this guidance. The figure legends for Figures 2–6 and the relevant Supplementary Figures have been updated to report the statistical test used, and sample size. Because each figure contains numerous significant results, listing all statistical values directly in the legends would compromise readability. Instead, the legends now refer to the existing Supplementary Tables, which provide the complete set of regression estimates, raw p-values, and FDR-corrected p-values.

9. *Figure 2: Please ensure that all heatmap scale bars are provided and labelled with titles and their appropriate units.*

   All heatmap panels in Figure 2 now include clearly labeled scale bars with visible tick labels. The displayed connectivity values represent normalized measures and therefore do not carry physical units.

10. *Please note our policy on code availability. If any codes were generated for data analysis for this manuscript (e.g. using R, MATLAB, or similar), please make this code available either via an online repository (and provide a link within the data availability statement), or within the manuscript.*

    We have revised the Data Availability statement to include additional details on the tools and software used at each stage of the processing pipeline. We have also provided a GitHub link containing the analysis scripts relevant to the framework described in this study.

11. *As a reminder at this revision stage, if you plan to edit the current figures and tables, or to make new figures and tables, please ensure that these items fit a typeset journal page (the area available for a figure or table is 170 mm x 210 mm), or are uploaded as supplementary material. Please view your figures at this size and make sure that all lettering and text are large enough to read.*

All revised figures and tables have been reformatted to fit within the journal's typeset area of 170 mm × 210 mm. Each figure was checked at 100% zoom at these dimensions to confirm that all text and graphic elements remain clearly legible. Larger items have been moved to Supplementary Material where necessary. This include moving labels for whole brain parcellation in Figure 1 to Supplementary Figure 1.

12. *Kindly note that if you have used BioRender software to create images in this manuscript, please provide the associated license for each image. If it is used in the graphical abstract, please add the phrase 'created in BioRender' within the image, and the link created after making the figure publicly available on the BioRender website. If it is used in a figure within the paper, please add the phrase' created in Biorender' within the figure legend, and the link created after making the figure publicly available on the BioRender website.*

No figures in this manuscript or the graphical abstract were created using BioRender. Therefore, no BioRender licenses or attributions are required.

References

1. Dickie EW, Anticevic A, Smith DE, et al. Ciftify: A framework for surface-based analysis of legacy MR acquisitions. *Neuroimage*. 2019;197. doi:10.1016/j.neuroimage.2019.04.078

2. Grayson DS, Fair DA. Development of large-scale functional networks from birth to adulthood: A guide to the neuroimaging literature. *Neuroimage*. 2017;160. doi:10.1016/j.neuroimage.2017.01.079

3. Marek S, Tervo-Clemmens B, Calabro FJ, et al. Reproducible brain-wide association studies require thousands of individuals. *Nature*. 2022;603(7902). doi:10.1038/s41586-022-04492-9

4. Hagler DJ, Hatton SN, Cornejo MD, et al. Image processing and analysis methods for the Adolescent Brain Cognitive Development Study. *Neuroimage*. 2019;202. doi:10.1016/j.neuroimage.2019.116091

5. Stuart EA. Matching methods for causal inference: A review and a look forward. *Statistical Science*. 2010;25(1). doi:10.1214/09-STS313

6. Rosenbaum PR, Rubin DB. Constructing a control group using multivariate matched sampling methods that incorporate the propensity score. *American Statistician*. 1985;39(1). doi:10.1080/00031305.1985.10479383

7. Fornito A, Zalesky A, Bullmore ET. Network scaling effects in graph analytic studies of human resting-state fMRI data. *Front Syst Neurosci*. 2010;4. doi:10.3389/fnsys.2010.00022

8. Zalesky A, Fornito A, Harding IH, et al. Whole-brain anatomical networks: Does the choice of nodes matter? *Neuroimage*. 2010;50(3). doi:10.1016/j.neuroimage.2009.12.027

9. Cammoun L, Gigandet X, Meskaldji D, et al. Mapping the human connectome at multiple scales with diffusion spectrum MRI. *J Neurosci Methods*. 2012;203(2). doi:10.1016/j.jneumeth.2011.09.031
