# Supplementary material for: Multiscale connectivity framework for working memory network in paediatric acute lymphoblastic leukaemia survivors
Source: Brain Commun. 2026 Apr 17;8(2):fcag137. doi: 10.1093/braincomms/fcag137 (PMC13126661; doi:10.1093/braincomms/fcag137)
Supplement: fcag137_Supplementary_Data [file Revision_1_ Response_to_reviewers_comments.pdf › Revision_1_ Response_to_reviewers_comments.pdf]

Dear Editor,

I checked the submitted files. The main manuscript file is the revised version of the original manuscript. The revised manuscript is with Track Changes on (a tracked version) so you can see all edits that I made to the original manuscript, the figures are deleted in the revised manuscript.

Regards,
Rajikha
