# Supplementary material for: Multiscale connectivity framework for working memory network in paediatric acute lymphoblastic leukaemia survivors
Source: Brain Commun. 2026 Apr 17;8(2):fcag137. doi: 10.1093/braincomms/fcag137 (PMC13126661; doi:10.1093/braincomms/fcag137)
Supplement: fcag137_Supplementary_Data [file Revision_1_decision_letter.pdf › Revision_1_decision_letter.pdf]

From: brcoms.editorialoffice@paeditorial.co.uk
To:
CC:
BCC:
Subject: Brain Communications - Decision on Manuscript ID BRAINCOM-2025-1002.R1
Body: 04-Feb-2026

Dear Dr. Raja,

Thank you for your recent submission BRAINCOM-2025-1002.R1
entitled "Multiscale Connectivity Framework for Working Memory Network in Pediatric Acute Lymphoblastic Leukemia Survivors" to Brain Communications.

I am pleased to let you know that we would like to provisionally accept your manuscript entitled "Multiscale Connectivity Framework for Working Memory Network in Pediatric Acute Lymphoblastic Leukemia Survivors" for publication in Brain Communications. There are still some minor points for you to address before final acceptance, but further peer-review will not be necessary. However, please note that we will not accept the paper unless you comply with our policies on data analysis and presentation as noted in the comments from the Scientific Editor.

Please respond to the comments shown below from reviewers and/or our Scientific Editor and submit a revised version.

Please format your revised manuscript according to our Instructions to Authors
https://academic.oup.com/braincomms/pages/General_Instructions
A summary of key points is attached to help with this. Please note that there can be substantial delays to publication if you don't resubmit in line with our requirements.

To revise your manuscript, click the link below or log into https://mc.manuscriptcentral.com/braincom and enter your Author Centre, where you will find your manuscript title listed under "Manuscripts with Decisions." Under "Actions," click on "Create a Revision." Your manuscript number will be appended to denote a revision.

*** PLEASE NOTE: This is a two-step process. After clicking on the link, you will be directed to a webpage to confirm. ***

https://mc.manuscriptcentral.com/braincom?URL_MASK=52495c6338cd4f7baae66371684ed4e1

Please upload a revised manuscript with the changes you have made shown either with "track changes" or in coloured text.

When submitting your revised manuscript, you will be asked to respond to the reviewer(s) and editor's comments. Please be as specific as possible in your response to all of the comments.

The editorial team at Brain Communications also aims to increase transparency in scientific publishing and to demonstrate the value that peer review brings to the scientific process. Please therefore consider whether you are willing to have reviewer comments on your paper and your response published as supplementary data with the paper if it is accepted. This is entirely optional and will only be done if both the authors and reviewers agree. When submitting your revision you will have the opportunity to update the answer you entered previously.

IMPORTANT: Your original files are available to you when you upload your revised manuscript. Please delete any redundant files before completing the submission.

Because we are trying to facilitate timely publication of manuscripts submitted to Brain Communications, we would appreciate your revised manuscript as soon as possible. If it is not possible for you to submit your revision within 3 months, the option to submit a revision will expire, and we will consider your revised paper as a new submission. However, if you need a little more time, please get in touch and we can extend this deadline for you.

Please note that Brain Communications is a fully open access journal, and if your article is accepted for publication, an open access charge will be payable (this does not apply to letters and scientific commentaries). Details can be found in our Instructions for Authors: https://academic.oup.com/braincomms/pages/General_Instructions

Once again, thank you for submitting your manuscript to Brain Communications and I look forward to receiving your revision.

Best wishes

Ernie Ho
Scientific Editor Team, Brain Communications

Professor Tara Spires-Jones
Founding Editor, Brain Communications

Reviewer(s)' Comments to Author:

Reviewer: 1

Comments to the Author
The authors have addressed all my concerns

Comments from Scientific Editor Team:
- Thank you for providing a graphical abstract, however, we may still need you to make some changes to it to make the main research question(s) and the main finding(s) of the study clearer. Authors should strive to make the graphical abstract informative, interesting, and visually appealing (no separate caption is needed; abbreviations are allowed only for gene

names).

- Supp figure 1: could you please define the abbreviations that have not been defined yet, including but not limited to PVC, EVC, EAC etc
